# A Benziodoxole-Based Hypervalent Iodine(III) Compound Functioning as a Peptide Coupling Reagent

**DOI:** 10.3389/fchem.2020.00183

**Published:** 2020-03-18

**Authors:** Li-Jun Qiu, Dan Liu, Ke Zheng, Ming-Tao Zhang, Chi Zhang

**Affiliations:** ^1^State Key Laboratory of Elemento-Organic Chemistry, Collaborative Innovation Center of Chemical Science and Engineering (Tianjin), College of Chemistry, Nankai University, Tianjin, China; ^2^Computational Center for Molecular Science, Nankai University, Tianjin, China

**Keywords:** peptide, IBA-OBz, hypervalent iodine(III) reagent, solid-phase peptide synthesis (SPPS), DFT calculations

## Abstract

1-Benzoyloxy-1,2-benziodoxol-3-(1H)-one (IBA-OBz), a readily available and bench stable benziodoxole-based iodine(III) reagent, can be employed for the synthesis of dipeptides from various standard and sterically hindered amino acids in the presence of (4-MeOC_6_H_4_)_3_P. The combined system of IBA-OBz/(4-MeOC_6_H_4_)_3_P is also successfully applied to the solid-phase peptide synthesis and a pentapeptide leu-enkephalin in unprotected form has been synthesized. Density functional theory calculations reveal that the rate-limiting step is nucleophilic attack of 4-dimethylaminopyridine (DMAP) onto IBA-OBz.

## Introduction

**Scheme 1:**
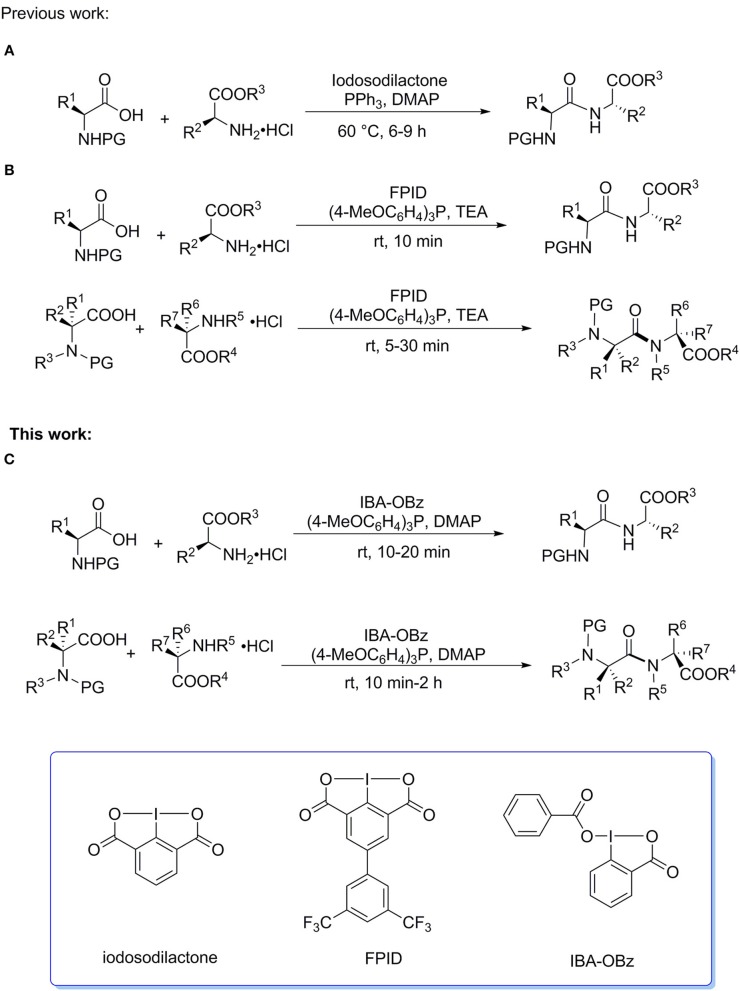
Peptide Synthesis Mediated by Hypervalent lodine(lll) Reagents. **(A)** Peptide coupling of standard amino acids mediated by iodosodilactone. **(B)** Peptide coupling of standard and sterically hindered amino acids mediated by FPID. **(C)** Peptide coupling of standard and sterically hindered amino acids mediated by IBA-OBz.

Hypervalent iodine reagents have attracted considerable attention of synthetic chemists due to their rich reactivities, low toxicity, ready, availability, and recyclability (Varvoglis, 1997; Zhdankin and Stang, 2002, 2008; Wirth, 2005; Brown et al., 2013; Singh and Wirth, 2014; Zhdankin, 2014; Duan et al., 2016; Li et al., 2016; Yoshimura and Zhdankin, 2016; Han and Zhang, 2018; Liu et al., 2019). In 2012, we first reported that iodosodilactone, a bicyclic benziodoxole compound, could function as a coupling reagent to promote the efficient syntheses of esters, macrocyclic lactones, amides, and peptides in the presence of PPh_3_ at 60^o^C (Scheme 1) (Tian et al., 2012). We subsequently designed and synthesized a new powerful analog of iodosodilactone, FPID, which could mediate the peptide coupling reactions of standard amino acids and sterically hindered amino acids in good to high yields within 30 min in the presence of (4-MeOC_6_H_4_)_3_P at room temperature (Scheme 1B) (Zhang et al., 2015). Recently, we successfully applied this system to the solid-phase peptide synthesis and cyclic peptide synthesis (Liu et al., 2018). Notably, FPID can be readily regenerated after reaction, and iodosodilactone shares the same feature, which is not provided in the existing peptide coupling reagents (Constable et al., 2007; EI-Faham and Albericio, 2011). Although FPID has the advantages of high efficiency, wide range of substrates ranging from standard amino acids to sterically hindered amino acids, and recyclability in peptide synthesis reactions, it should be prepared after four steps reaction of Suzuki coupling, diazotization/iodination, oxidation by potassium permanganate, and oxidation by aqueous sodium hypochlorite solution using commercially available 4-bromo-2,6-dimethylaniline and 3,5-bis(trifluoromethyl)-phenyl boronic acid as starting materials. Apparently, FPID is not readily available, which would have an adverse effect on its practical use. Thus, it is necessary to develop readily available and efficient iodine(III)-based peptide coupling reagents to promote their practical application in oligopeptide synthesis. IBA-OBz is a benziodoxole-based iodine(III) compound which can be prepared by one-step reaction from a commercial available I(III) compound IBA-OAc. We speculated that the structure of IBA-OBz would have similarity to FPID when taking account of two benzoyloxy ligands attached to central iodine(III) atom, furthermore, we assumed that it would exhibit a similar reactivity to FPID in peptide coupling reaction. Herein, we reported that an efficient preparation of IBA-OBz and its synthetic utility as an efficient coupling reagent to promote the synthesis of dipeptides and the solid-phase peptide synthesis of a pentapeptide leu-enkephalin. (Scheme 1C).

## Results and Discussion

**Scheme 2:**
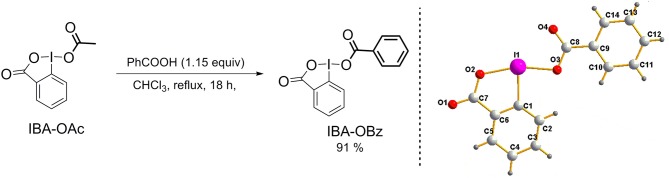
Synthesis and X-ray crystal structure of IBA-OBz.

IBA-OBz was readily prepared via a single-step process (Scheme 2). On the basis of the previous method (Mocci et al., 2007), a ligand exchange reaction between commercially available IBA-OAc and benzoic acid (1.15 equiv) in anhydrous CHCl_3_ [0.5 M of IBA-OAc] furnished IBA-OBz in 91% yield as a colorless solid. Notably, IBA-OBz could be prepared on a large scale (20 mmol, 5.9 g) in 81% yield and stored for 1 year at room temperature without detectable decomposition.

The structure of IBA-OBz was elucidated by means of NMR spectroscopy and X-ray crystallography (for details, see Figure S1 and Table S1). The ^13^C NMR signal for the carbon atom connected to the iodine(III) central atom was found at 118.8 ppm, which was in the typical region for hypervalent iodine(III) compounds. Moreover, the single crystal structure of IBA-OBz was firstly obtained from chloroform/diethyl ether. The X-ray crystallography revealed that the O2-I1-O3 bond angles (163.95°) was <180° and the O3-I1-C1 and O2-I1-C1 bond angles (84.40° and 79.68°, respectively) deviated from 90°, which demonstrated IBA-OBz had the distorted T-shaped structure.

We firstly chose Boc-_L_-Phe-OH and H-_L_-Leu-OMe·HCl as two model substrates to test whether IBA-OBz can mediate peptide synthesis (Table 1). To our delight, the desired dipeptide Boc-Phe-Leu-OMe was obtained in 66% yield within 10 min upon treatment with 1.2 equiv of IBA-OBz, 1.0 equiv of (4-MeOC_6_H_4_)_3_P, and 3.0 equiv of TEA in DCE at room temperature (Table 1, entry 1). Subsequently various organic solvents including DCM, CHCl_3_, THF, EtOAc, acetone, CH_3_CN, toluene, and DMF were screened. However, none of these solvents gave better yields than that in DCE (entries 2–9). Thereafter, DCE was selected as the solvent of choice, and other organic bases were accordingly screened in DCE. The results showed that 1,8-diazabicyclo[5.4.0]undec-7-ene (DBU), DMAP, *N,N*-diisopropylethylamine (DIPEA), 4-methylmorpholine (NMM), *N*-methylimidazole (NMI), and 4-pyrrolidinopyridine gave the product in 30–80% yields, in which DMAP was the best one (entries 10–15). When 1.5 equiv of both IBA-OBz and (4-MeOC_6_H_4_)_3_P were used, the yield could be improved to 86% (entry 16). Therefore, we obtained the optimal reaction conditions in which 1.5 equiv of both IBA-OBz and (4-MeOC_6_H_4_)_3_P and 3.0 equiv of DMAP were used in DCE (0.04 M) at room temperature.

**Table 1:** Optimization of reaction conditions for Boc-Phe-Leu-OMe synthesis*^a^*.

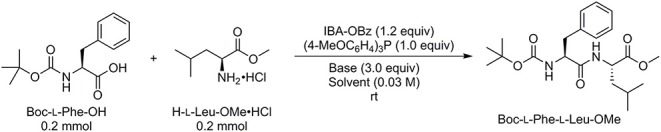				
**Entry**	**Solvent**	**Base**	**Time (min)**	**Yield (%)^b^**
1	DCE	TEA	10	66
2	CHCl_3_	TEA	10	48
3	DCM	TEA	10	48
4	THF	TEA	10	31
5	EtOAc	TEA	10	61
6	Acetone	TEA	10	27
7	CH3CN	TEA	10	39
8	Toluene	TEA	300	43
9	DMF	TEA	10	56
10	DCE	DBU	10	30
11	DCE	DMAP	10	80
12	DCE	DIPEA	10	62
13	DCE	NMM	20	60
14	DCE	NMI	10	47
15	DCE	4-Pyrrolidinopyridine	10	68
16*^c^*	DCE	DMAP	10	86

a *Reaction conditions: Boc-_L_-Phe-OH (0.2 mmol), H-_L_-Leu-OMe·HCl (0.2 mmol), IBA-OBz (0.24 mmol), (4-MeOC_6_H_4_)_3_P (0.2 mmol), Base (0.6 mmol), Solvent (5 mL)*.

b *Isolated yield*.

c *The optimal reaction conditions: Boc-_L_-Phe-OH (0.2 mmol), H-_L_-Leu-OMe·HCl (0.2 mmol), IBA-OBz (0.3 mmol), (4-MeOC_6_H_4_)_3_P (0.3 mmol), DMAP (0.6 mmol), DCE (5 mL)*.

With the optimal conditions in hand, we first evaluated the performance of IBA-OBz for the synthesis of dipeptides from various standard amino acids (Table 2). All dipeptides were obtained in moderate to excellent yields within 10–20 min. This protocol was successfully applied to different amino protecting groups including Boc and Cbz, without detriment to yields (Table 2, entries 4, 5). Nevertheless, TEA was used instead of DMAP to avoid deprotection when amino protecting group was Fmoc (entry 6). When Cbz-Ala-OH was used as the carboxylic acid partner, moderate to high yields were obtained (entries 8–10). It is unnecessary to protect the indole ring of tryptophan in the peptide coupling reaction mediated by IBA-OBz (entry 13). Notably, for amino acids containing unprotected hydroxyl groups, such as serine (entry 17), threonine (entry 18) and tyrosine (entry 19), the use of TEA instead of DMAP could improve the coupling efficiency. Remarkably, reactions of sterically hindered amino acids such as valine (entries 20–22) and proline (entries 10, 22–26) also worked well, providing the corresponding dipeptides in yields up to 93%. Compared to peptide coupling reaction mediated by FPID, the reaction yields of Cbz-_L_-Phe-_L_-Thr-OMe (entry 18) and Boc-_L_-Val-_L_-Pro-OMe (entry 22) still needed to be improved.

**Table 2:** IBA-OBz mediated synthesis of dipeptides from standard amino acids*^a^*.

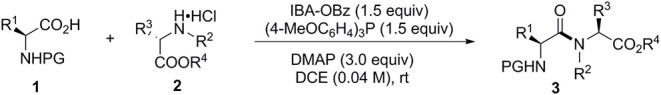			
**Entry**	**Dipeptide**	**Time (min)**	**Yield (%)^b^**
1	Boc-_L_-Phe-_L_-Leu-OMe (**3-1**)	10	86
2	Boc-Gly-Gly-OMe (**3-2**)	10	81
3	Boc-_L_-Leu-_L_-Lys(Z)-OMe (**3-3**)	10	84
4	Boc-_L_-Leu-_L_-Ala-OMe (**3-4**)	10	85
5	Cbz-_L_-Leu-_L_-Ala-OMe (**3-5**)	10	78
6^c^	Fmoc-_L_-Leu-_L_-Ala-OMe (**3-6**)	10	84
7	Cbz-_L_-Leu-_L_-Lys(Z)-OMe (**3-7**)	10	91
8	Cbz-_L_-Ala-_L_-His(Trt)-OMe (**3-8**)	10	88
9	Cbz-_L_-Ala-_L_-Cys(Trt)-OMe (**3-9**)	20	75
10	Cbz-_L_-Ala-_L_-Pro-OMe (**3-10**)	10	59
11	Cbz-_L_-Met-Gly-OMe (**3-11**)	10	77
12	Cbz-_L_-Met-Gly-OEt (**3-12**)	10	89
13	Cbz-_L_-Trp-_L_-Leu-OMe (**3-13**)	10	80
14	Cbz-_L_-Asn(Trt)-_L_-Leu-OMe (**3-14**)	10	75
15	Cbz-_L_-Phe-_L_-Ile-OMe (**3-15**)	10	84
16	Cbz-_L_-Phe-_L_-Tyr(Bzl)-OMe (**3-16**)	10	73
17*^c^*	Cbz-_L_-Phe-_L_-Ser-OMe (**3-17**)	10	60
18*^c^*	Cbz-_L_-Phe-_L_-Thr-OMe (**3-18**)	10	42
19*^c^*	Cbz-_L_-Phe-_L_-Tyr-OMe (**3-19**)	10	84
20	Boc-_L_-Val-_L_-Val-OMe (**3-20**)	10	75
21	Cbz-_L_-Val-_L_-Glu(OEt)-OEt (**3-21**)	20	70
22	Boc-_L_-Val-_L_-Pro-OMe (**3-22**)	10	46
23	Boc-_L_-Pro-_L_-Ala-OMe (**3-23**)	10	84
24	Boc-_L_-Pro-_L_-Leu-OMe **(3-24**)	10	93
25	Boc-_L_-Leu-_L_-Pro-OMe (**3-25**)	10	63
26	Cbz-Gly-_L_-Pro-OMe (**3-26**)	10	83

a *Conditions: N-PG-AA_1_-OH (0.2 mmol), H-AA_2_-OMe·HCl (0.2 mmol), IBA-OBz (0.3 mmol), (4-MeOC_6_H_4_)_3_P (0.3 mmol), DMAP (0.6 mmol), DCE (5 mL)*.

b *Isolated yield*.

c *TEA was used instead of DMAP*.

Sterically hindered amino acids such as *N*-methylamino acids and α,α-disubstituted amino acids are widely present in a variety of naturally occurring peptides and pharmaceuticals (the antihypertension drug lisinopril) (Slomczynska et al., 1992; Wenschuh et al., 1995; Humphrey and Chamberlin, 1997). However, the synthesis of peptides containing sterically hindered amino acids has limitation due to poor yields using conventional coupling reagents (i.e., DCC/HOBT) (Balasubramanian et al., 1981; Leibfritz et al., 1982). The existing processes generally require the preparation of activated amino acid derivatives to achieve the desired results (Katritzky et al., 2007; Brown and Schafmeister, 2008). Hence, we evaluated the IBA-OBz/(4-MeOC_6_H_4_)_3_P system with several sterically hindered amino acids (Table 3). Most dipeptides were obtained in moderate to high yields within 10–60 min. It was worth noting that widely used amino protecting group Boc, which decomposed readily using BOP system, was compatible with the present reaction system (Table 3, entries 1–5). Coupling reactions between α,α-disubstituted amino acid (H-Aib-OMe) and a series of standard amino acids provided the corresponding dipeptides in 59–77% yields (entries 6–10). Notably, the coupling reaction proceeded smoothly even when both amino acids were sterically hindered, affording dipeptides in moderate to good yields (entries 11–13). Reactions of *N*-methylamino acids (Cbz-*N*(Me)-Phe-OH) with various standard amino acids produced the corresponding dipeptides in yields up to 91% (entries 14–22). Furthermore, coupling efficiency was improved by using TEA instead of DMAP when tyrosine was a component of dipeptides (entries 4, 22).

**Table 3:** IBA-OBz mediated synthesis of dipeptides from sterically hindered amino acids*^a^*.

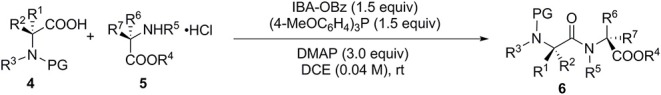			
**Entry**	**Dipeptide**	**Time (min)**	**Yield (%)^b^**
1	Boc-_L_-Ala-Aib-OMe (**6-1**)	40	79
2	Boc-Gly-Aib-OMe (**6-2**)	10	72
3	Boc-_L_-Leu-Aib-OMe (**6-3**)	10	67
4 *^c^*	Boc-_L_-Tyr-Aib-OMe (**6-4**)	10	70
5	Boc-_L_-Pro-Aib-OMe (**6-5**)	120	81
6	Cbz-_L_-Trp-Aib-OMe (**6-6**)	20	77
7	Cbz-_L_-Phe-Aib-OMe (**6-7**)	20	59
8	Cbz-_L_-Ala-Aib-OMe (**6-8**)	40	62
9	Cbz-_L_-Met-Aib-OMe (**6-9**)	40	63
10	Cbz-_L_-Ser(tBu)-Aib-OMe (**6-10**)	10	68
11	Cbz-_L_-Val-Aib-OMe (**6-11**)	50	46
12	Cbz-_L_-NMePhe-Aib-OMe (**6-12**)	20	72
13	Cbz-_L_-NMePhe-_L_-Val-OMe (**6-13**)	10	83
14	Cbz-_L_-NMePhe-Gly-OMe (**6-14**)	30	90
15	Cbz-_L_-NMePhe-_L_-Ala-OMe (**6-15**)	10	81
16	Cbz-_L_-NMePhe-_L_-Ile-OMe (**6-16**)	10	86
17	Cbz-_L_-NMePhe-_L_-His(Trt)-OMe (**6-17**)	10	81
18	Cbz-_L_-NMePhe-_L_-Tyr(Bzl)-OMe (**6-18**)	10	90
19	Cbz-_L_-NMePhe-_L_-Cys(Trt)-OMe (**6-19**)	10	80
20	Cbz-_L_-NMePhe-_L_-Lys(Z)-OMe (**6-20**)	10	88
21	Cbz-_L_-NMePhe-_L_-Glu(OEt)-OEt (**6-21**)	10	82
22*^c^*	Cbz-_L_-NMePhe-_L_-Tyr-OMe (**6-22**)	10	91

a *Conditions: N-PG-AA_1_-OH (0.2 mmol), H-AA_2_-OMe·HCl (0.2 mmol), IBA-OBz (0.3 mmol), (4-MeOC_6_H_4_)_3_P (0.3 mmol), DMAP (0.6 mmol), DCE (5 mL)*.

b *Isolated yield*.

c *TEA was used instead of DMAP*.

**Scheme 3:**
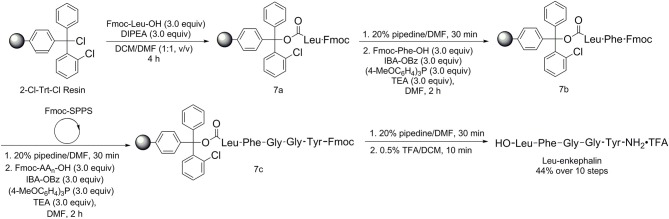
Synthesis of unprotected Leu-enkephalin by solid-phase peptide synthesis.

In order to verify the application of IBA-OBz/(4-MeOC_6_H_4_)_3_P system in the solid-phase peptide synthesis (SPPS), we chose unprotected Leu-enkephalin as the target short peptide, which was isolated from pig brains and had the effect of modifying neurotransmitters (Hughes et al., 1975; Coste et al., 1991). A commercially available 2-chlorotriphenylmethyl chloride resin (2-Cl-Trt-Cl Resin) was used as a carrier for the solid-phase peptide synthesis under standard conditions of Fmoc-solid-phase peptide synthesis (Fmoc-SPPS). The unprotected Leu-enkephalin was synthesized following the route (Scheme 3). The carboxylic group of Fmoc-Leu-OH was connected to 2-Cl-Trt-Cl resins to obtain the 2-chlorotrityl resin-bound Leu(Fmoc) (**7a**) in the presence of 3.0 equiv of *N,N*-diisopropylethylamine in DCM/*N,N*-dimethylformamide (DMF) (v/v = 1:1) within 4 h. The protecting group Fmoc was then removed from **7a** upon the treatment of 20% piperidine/DMF within 30 min. Subsequently the peptide coupling reaction of Fmoc-Phe-OH with the 2-chlorotrityl resin-bound Leu produced 2-chlorotrityl resin-bound Leu-Phe dipeptide **7b** using 3.0 equiv of IBA-OBz, 3.0 equiv of (4-MeOC_6_H_4_)_3_P, and 3.0 equiv of TEA in DMF at room temperature within 2 h. The N-terminus of **7b** was then sequentially extended with the Fmoc-Gly-OH, Fmoc-Gly-OH, Fmoc-Tyr-OH units using the standard Fmoc solid-phase peptide synthesis (SPPS) procedure to give **7c**. After Fmoc group was removed from **7c**, the pentapeptide chain was then cleaved from the resin upon the treatment of 0.5% trifluoroacetic acid/DCM to give the desired pentapeptide Leu-enkephalin. The Leu-enkephalin was purified by reversed-phase HPLC and it was finally obtained in moderate yield (44% over 10 steps). This successful synthesis of the N-,C-unprotected Leu-enkephalin demonstrated that the IBA-OBz/(4-MeOC_6_H_4_)_3_P system was suitable not only in solution phase peptide synthesis but also in solid-phase peptide synthesis.

**Figure 1:**
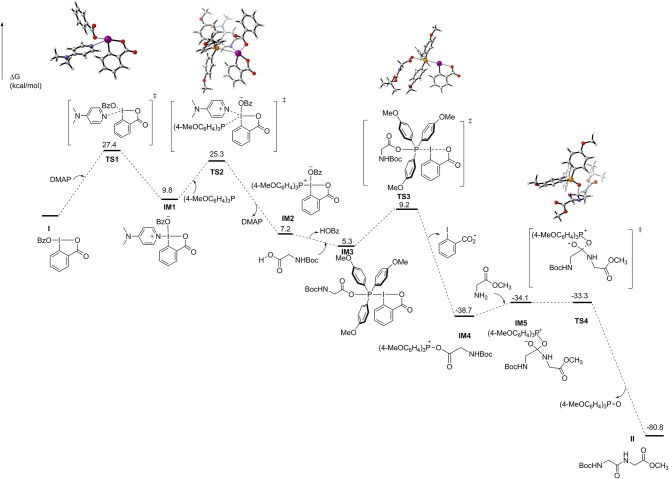
Energy diagram for IBA-OBz-mediated peptide formation calculated at the M06-2X/[6-311++G(2df,2p) + SDD(I)] (SMD)//M06-2X/[6-31+G(d) + Lanl2dz(I)] (SMD) level. Free energies are in kilocalories per mole, and bond lengths are in angstroms.

To shed light on the reaction mechanism of this peptide bond formation reaction mediated by IBA-OBz, we performed DFT computations (Liu et al., 2009; Schoenebeck and Houk, 2010; Zhou and Li, 2015) to characterize the pathways for the reaction of Boc-Gly-OH and H-Gly-OMe·HCl with IBA-OBz (for details, see Supporting Information). As illustrated in Figure 1, the reaction would proceed through substitution of the OBz group of IBA-OBz with DMAP, generating zwitterion **IM1** via **TS1** by crossing a barrier of 27.4 kcal/mol (**TS1**). ESI-mass analysis of a reaction mixture containing IBA-OBz, DMAP, and (4-MeOC_6_H_4_)_3_P in DCE under room temperature would yield a peak at m/z = 491.2082, which was assigned to be [**IM1**+H]^+^ (for details, see the Figure S2). Subsequently, the nucleophilic attack of (4-MeOC_6_H_4_)_3_P to zwitterion **IM1** resulted in zwitterion **IM2** by crossing a barrier of 25.3 kcal/mol (**TS2**). The carboxylic group of Boc-Gly-OH would attack **IM2** at the phosphorus center to give **IM3**, following with the leaving of benzoic acid anion via **TS3** to produce **IM4**, which was predicted to require an activation free energy of 3.9 kcal/mol. Then, the **IM4** was attacked by amino group of H-Gly-OMe to afford **IM5**, which had a barrier of only 4.6 kcal/mol. Subsequent (4-MeOC_6_H_4_)_3_P=O dissociation through **TS4** to generate dipeptide product **II**, which was calculated to have a barrier of only 0.8 kcal/mol. Reviewing all of the calculated energy profile, the overall reaction was exergonic by 80.8 kcal/mol and thus highly thermodynamically favorable. The formation of **IM1** via **TS1** constituted to be a rate-limiting step of the reaction with an energy barrier of 27.4 kcal/mol.

**Scheme 4:**
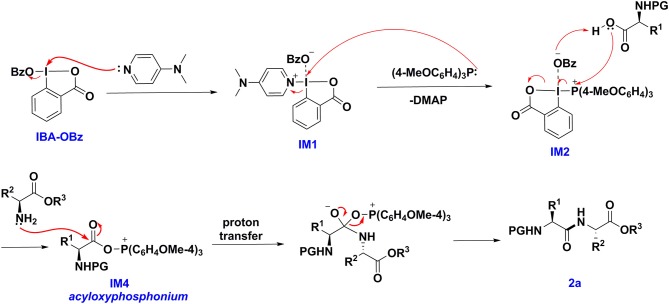
Proposed mechanism for IBA-OBz/(4-MeOC6H4)3P mediated peptide-coupling reaction.

On the basis of experimental and computational studies, we proposed a reaction mechanism (Scheme 4). IBA-OBz was initially activated by DMAP to form the zwitterion **IM1**. Zwitterion **IM1** then would undergo the ligand exchange with (4-MeOC_6_H_4_)_3_P to give the zwitterion **IM2**, which reacted with the carboxylic group of Boc-Gly-OH to form a key intermediate acyloxyphosphonium intermediate **IM4**. Subsequently intermediate **IM4** was attacked by the amino group of H-Gly-OMe to afford a dipeptide **II** and by-product tris(4-methoxyphenyl)phosphine oxide.

## Conclusion

In conclusion, we reported a peptide coupling reaction mediated by a readily available, bench stable benziodoxole-based iodine(III) reagent IBA-OBz. In combination with tris(4-methoxyphenyl)phosphine and an organic base (DMAP or TEA), the reagent system not only effectively mediated the peptide coupling reaction of standard amino acids and sterically hindered amino acids in the solution-phase peptide synthesis, but also could be used in the solid-phase peptide synthesis. DFT calculations revealed that the formation of **IM1** was a rate-limiting step of the peptide coupling reaction and the presence of **IM1** was supported by its ESI mass spectrum.

## Materials and Methods

### Experimental and Computational Details

The ^1^H NMR spectra were recorded at 400 MHz and ^13^C NMR spectra were measured at 100 MHz using a Bruker AV400 instrument with CDCl3 as the solvent. The chemical shifts (δ) were measured in ppm and with the solvents as references (For CDCl3, ^1^H: δ = 7.26 ppm, ^13^C: δ = 77.00 ppm). The multiplicities of the signals are described using the following abbreviations: s = singlet, d = doublet, t = triplet, q = quartet, m = multiplet, dd = doublet of doublets, br = broad. High resolution mass spectral analyses (HRMS) were performed on a high resolution ESI-FTICR mass spectrometer (Varian 7.0 T). High performance liquid chromatography (HPLC) analysis was conducted using Shimadzu LC-10 AD coupled diode array-detector SPD-MA-10A-VP and chiral column of Daicel CHIRALCEL OD-H (4.6 mm−25 cm), AD-H (4.6 mm−25 cm), or AS-H (4.6 mm−25 cm). Melting points were recorded on a RY-1 type apparatus. All solvents were obtained from commercial sources and were purified according to standard procedures. Petroleum ether (PE), where used, had the boiling point range 60–90°C.

The M06-2X functional in conjugation with a mixed basis set of the Lanl2dz (Hay and Wadt, 1985) pseudopotential for iodine and 6-31G(d) for all other atoms was used for optimizing the geometry of all the minima and transition states in solution. The universal solvation model (SMD) was employed to account for the effects of DCE solution (Marenich et al., 2009). All the optimized structures were confirmed by frequency calculations to be either minima or transition states at the same level of theory. To obtain more accurate electronic energies, single point energy calculations were performed at the M06-2X/[6-311++G(2df, 2p) + SDD(I)](SMD) level with the M06-2X/[6-31G(d) + Lanl2dz(I)](SMD) structures. Computed structures were illustrated with CYLView (Legault, 2009). All quantum mechanical calculations were performed using Gaussian 16 packages (Frisch et al., 2016).

## Chemical Synthesis

### Synthesis of IBA-OBz

IBA-OAc (1.53 g, 5 mmol) and benzoic acid (702.1 mg, 5.75 mmol) were added to a 50 mL round-bottom flask, then CHCl_3_ (15 mL) was added and the mixture was refluxed for 18 h. Then CHCl_3_ was concentrated in vacuo and 30 mL of petroleum ether was added. The mixture was stirred at 60°C for 1 h, and filtered to give a crude product. The crude product was dissolved in CHCl_3_, washed sequentially with NaHCO_3_, H_2_O, brine, and dried over anhydrous MgSO_4_. The solvent was evaporated to give the desired product IBA-OBz (1.68 g, 91%).

Colorless solid; yield: 91%. ^1^H NMR (400 MHz, CDCl_3_) δ = 8.30 (dd, *J* = 7.6, 1.6 Hz, 1H), 8.13–8.08 (m, 3H), 8.01–7.97 (m, 1H), 7.76 (dd, *J* = 7.4, 0.4 Hz, 1H), 7.66–7.61 (m, 1H), 7.51 (d, *J* = 7.6 Hz, 2H). ^13^C NMR (100 MHz, CDCl_3_) δ = 171.2, 168.1, 136.3, 133.5, 133.3, 131.4, 130.1, 129.2, 129.0, 128.8, 128.6, 118.8. HRMS(ESI): *m*/*z* calcd. for C_14_H_9_IO_4_ [M+H]^+^: 368.9618, found 368.9604.

### Synthesis of 3-1 to 3-26 and 6-1 to 6-22

To a mixture of PG-AA_1_-OH (0.2 mmol) and H-AA_2_-OMe·HCl (0.2 mmol) in DCE (5 mL) in a 25 mL round-bottom flask was added DMAP (0.6 mmol). The solution was stirred at room temperature for 5 min. Then, IBA-OBz(0.3 mmol) and (4-MeOC_6_H_4_)_3_P (0.3 mmol) was added in sequence. The resulting mixture was stirred at room temperature and monitored by TLC. After 10 min, the reaction was quenched with saturated aq. sodium bicarbonate solution (10 mL) and extracted with EtOAc (50 mL × 3). The combined organic layer was washed with brine, dried over anhydrous MgSO_4_, and concentrated in vacuo to afford the crude product, which was then purified by silica gel flash chromatography to give the pure dipeptide product PG-AA_1_-AA_2_-OMe.

### Boc-_L_-Phe-_L_-Leu-OMe (3-1)

Colorless solid; yield: 86%. ^1^H NMR (400 MHz, CDCl_3_) δ = 7.31–7.21 (m, 5H), 6.24 (d, *J* = 7.6 Hz, 1H), 4.99 (s, 1H), 4.58–4.54 (m, 1H), 4.35 (d, *J* = 6.8 Hz, 1H), 3.69 (s, 3H), 3.07 (d, *J* = 6.5 Hz, 2H), 1.63–1.41 (m, 12H), 0.92–0.88 (m, 6H); ^13^C NMR (100 MHz, CDCl_3_) δ = 172.8, 170.9, 155.4, 136.6, 129.3, 128.6, 126.9, 80.2, 55.6, 52.2, 50.7, 41.5, 38.0, 28.2, 24.6, 22.7, 21.8.

### Boc-Gly-Gly-OMe (3-2)

Light yellow solid, yield: 81%. ^1^H NMR (400 MHz, CDCl_3_) δ = 6.59 (s, 1H), 5.12 (s, 1H), 4.07 (d, *J* = 5.6 Hz, 2H), 3.85 (d, *J* = 6.0 Hz, 2H), 3.76 (s, 3H), 1.46 (s, 9H); ^13^C NMR (100 MHz, CDCl_3_) δ = 170.2, 169.8, 156.1, 80.4, 52.4, 44.2, 41.0, 28.2.

### Boc-_L_-Leu-_L_-Lys(Z)-OMe (3-3)

Colorless oily liquid; yield: 84%. ^1^H NMR (400 MHz, CDCl_3_) δ = 7.36–7.26 (m, 5H), 6.84 (s, 1H), 5.32 (d, *J* = 13.6 Hz, 1H), 5.12–5.03 (m, 3H), 4.56–4.53 (m, 1H), 4.16 (s, 1H), 3.71 (s, 3H), 3.23–3.11 (m, 2H), 1.80–1.26 (m, 18H), 0.90–0.89 (m, 6H); ^13^C NMR (100 MHz, CDCl_3_) δ = 173.1, 172.5, 156.5, 155.9, 136.5, 128.4, 128.2, 128.0, 80.0, 66.6, 52.8, 52.3, 51.9, 41.0, 40.4, 31.7, 29.0, 28.3, 24.6, 22.9, 22.4, 21.6.

### Boc-_L_-Leu-_L_-Ala-OMe (3-4)

Colorless solid; yield: 85%. ^1^H NMR (400 MHz, CDCl_3_): δ = 6.63 (s, 1H), 4.91 (s, 1H), 4.60–4.52 (m, 1H), 4.11 (br s, 1H), 3.74 (s, 3H), 1.72–1.59 (m, 3H), 1.43 (s, 9H), 1.39 (d, *J* = 7.2 Hz, 3H), 0.93 (dd, *J*_1_ = 6.2 Hz, *J*_2_ = 4.2 Hz, 6H); ^13^C NMR (100 MHz, CDCl_3_): δ = 173.1, 172.2, 155.6, 79.9, 52.4, 47.8, 41.3, 28.2, 24.6, 22.9, 21.9, 18.1.

### Cbz-_L_-Leu-_L_-Ala-OMe (3-5)

Colorless solid; yield: 78%. ^1^H NMR (400 MHz, CDCl_3_) δ = 7.36–7.30 (m, 5H), 6.45 (d, *J* = 7.2 Hz, 1H), 5.18–5.11 (m, 3H), 4.58–4.54 (m, 1H), 4.20–4.19 (m, 1H), 3.75 (s, 3H), 1.72–1.50 (m, 6H), 1.40 (d, *J* = 7.2 Hz, 3H), 0.94 (d, *J* = 6.0 Hz, 6H); ^13^C NMR (100 MHz, CDCl_3_) δ = 173.1, 171.8, 156.2, 136.2, 128.5, 128.1, 128.0, 67.0, 53.4, 52.4, 48.0, 41.5, 24.6, 22.9, 21.9, 18.1.

### Fmoc-_L_-Leu-_L_-Ala-OMe (3-6)

Colorless solid; yield: 84%. ^1^H NMR (400 MHz, CDCl_3_) δ = 7.75 (d, *J* = 7.6 Hz, 2H), 7.58 (d, *J* = 5.6 Hz, 2H), 7.39 (d, *J* = 7.4 Hz, 2H), 7.30–7.26 (m, 2H), 6.62 (s, 1H), 5.36 (s, 1H), 4.59–4.53 (m, 1H), 4.44–4.34 (m, 2H), 4.25–4.19 (m, 2H), 3.73 (s, 3H), 1.67–1.55 (m, 3H), 1.39 (d, *J* = 7.2 Hz, 3H), 0.95–0.94 (m, 6H); ^13^C NMR (100 MHz, CDCl_3_) δ = 173.1, 171.7, 156.2, 143.8, 143.7, 141.3, 127.7, 127.1, 125.0, 120.0, 120.0, 67.0, 53.4, 52.5, 48.1, 47.2, 41.6, 24.6, 22.9, 22.0, 18.3.

### Cbz-_L_-Leu-_L_-Lys(Z)-OMe (3-7)

Colorless solid; yield: 91%. ^1^H NMR (400 MHz, CDCl_3_) δ = 7.31–7.26 (m, 10H), 6.82 (d, *J* = 7.6 Hz, 1H), 5.38 (d, *J* = 7.6 Hz, 2H), 5.10–5.02 (m, 4H), 4.55–4.50 (m, 1H), 4.27–4.26 (m, 1H), 3.72 (s, 3H), 3.20–3.06 (m, 2H), 1.81–1.79 (m, 2H), 1.61–1.60 (m, 3H), 1.49–1.43 (m, 3H), 1.28–1.27 (m, 2H), 0.91–0.90 (m, 6H); ^13^C NMR (101 MHz, CDCl_3_) δ = 172.5, 172.5, 156.6, 156.3, 136.5, 136.1, 128.5, 128.5, 128.2, 128.1, 128.0, 67.0, 66.6, 53.3, 52.4, 51.9, 41.3, 40.3, 31.6, 29.0, 24.5, 22.9, 22.1, 21.8.

### Cbz-_L_-Ala-_L_-His(Trt)-OMe (3-8)

Colorless solid; yield: 88%. ^1^H NMR (400 MHz, CDCl_3_) δ = 7.67 (d, *J* = 7.6 Hz, 1H), 7.36–7.26 (m, 16H), 7.11–7.08 (m, 6H), 6.54 (s, 1H), 5.63 (d, *J* = 6.8 Hz, 1H), 5.11–5.02 (m, 2H), 4.80–4.75 (m, 1H), 4.33–4.30 (m, 1H), 3.59 (s, 3H), 3.07–2.97 (m, 2H), 1.43–1.39 (m, 3H); ^13^C NMR (100 MHz, CDCl_3_) δ = 172.0, 171.3, 155.6, 142.1, 138.6, 136.4, 136.2, 129.6, 128.4, 128.0, 128.0, 127.9, 127.8, 119.6, 75.3, 66.6, 52.5, 52.1, 50.4, 29.4, 19.1.

### Cbz-_L_-Ala-_L_-Cys(Trt)-OMe (3-9)

Colorless oily liquid; yield: 75%. ^1^H NMR (400 MHz, CDCl_3_) δ = 7.39–7.19 (m, 20H), 6.22 (d, *J* = 6.8 Hz, 1H), 5.29 (d, *J* = 8.8 Hz, 1H), 5.15–5.06 (m, 2H), 4.54–4.50 (m, 1H), 4.20 (s, 1H), 3.70 (s, 3H), 2.72–2.61 (m, 2H), 1.36 (d, *J* = 7.2 Hz, 3H); ^13^C NMR (100 MHz, CDCl_3_) δ = 171.8, 170.5, 155.7, 144.2, 136.2, 129.4, 128.5, 128.1, 128.0, 126.9, 66.9, 52.6, 51.2, 50.2, 33.5, 18.8.

### Cbz-_L_-Ala-_L_-Pro-OMe (3-10)

Colorless oily liquid; yield: 59%. ^1^H NMR (400 MHz, CDCl_3_) δ = 7.34–7.26 (m, 5H), 5.64 (d, *J* = 7.6 Hz, 1H), 5.08 (s, 2H), 4.55–4.51 (m, 2H), 3.72–3.61 (m, 5H), 2.24–2.17 (m, 1H), 2.10–1.96 (m, 3H), 1.39 (d, *J* = 6.8 Hz, 3H); ^13^C NMR (100 MHz, CDCl_3_) δ = 172.3, 171.2, 155.6, 136.4, 128.4, 128.0, 127.9, 66.6, 58.6, 52.2, 48.2, 46.7, 28.8, 24.8, 18.2.

### Cbz-_L_-Met-Gly-OMe (3-11)

Colorless solid; yield: 77%. ^1^H NMR (400 MHz, CDCl_3_) δ = 7.36–7.26 (m, 5H), 6.70 (s, 1H), 5.55 (d, *J* = 7.2 Hz, 1H), 5.11 (s, 2H), 4.45–4.43 (m, 1H), 4.13–3.96 (m, 2H), 3.75 (s, 3H), 2.62–2.58 (m, 2H), 2.15–1.95 (m, 5H); ^13^C NMR (100 MHz, CDCl_3_) δ = 171.5, 170.0, 156.1, 136.0, 128.5 128.2, 128.0, 67.1, 53.7, 52.3, 41.1, 31.6, 29.9, 15.1.

### Cbz-_L_-Met-Gly-OEt (3-12)

Colorless solid; yield: 81%. ^1^H NMR (400 MHz, CDCl_3_) δ = 7.36–7.26 (m, 5H), 6.65 (s, 1H), 5.52 (s, 1H), 5.12 (s, 2H), 4.44–4.42 (m, 1H), 4.21 (d, *J* = 7.2 Hz, 2H), 4.10–4.06 (m, 1H), 4.00–3.94 (m, 1H), 2.60 (t, *J* = 7.0 Hz, 2H), 2.14–1.97 (m, 5H), 1.30–1.25 (m, 5H); ^13^C NMR (100 MHz, CDCl_3_) δ = 171.5, 169.5, 156.1, 136.0, 128.5, 128.1, 128.0, 67.0, 61.5, 53.6, 41.2, 31.6, 29.9, 15.1, 14.0.

### Cbz-_L_-Trp-_L_-Leu-OMe (3-13)

Colorless oily liquid; yield: 80%. ^1^H NMR (400 MHz, CDCl_3_) δ = 8.07 (s, 1H), 7.71 (d, *J* = 7.6 Hz, 1H), 7.37–7.28 (m, 6H), 7.20 (t, *J* = 7.6 Hz, 1H), 7.14–7.10 (m, 2H), 6.00 (s, 1H), 5.50 (s, 1H), 5.13 (s, 2H), 4.53–4.47 (m, 2H), 3.65 (s, 3H), 3.37–3.34 (m, 1H), 3.19–3.14 (m, 1H), 1.69–1.35 (m, 7H), 0.84–0.81 (m, 6H); ^13^C NMR (100 MHz, CDCl_3_) δ = 172.8, 171.0, 155.9, 136.2, 128.5, 128.1, 128.0, 127.3, 123.5, 122.2, 119.7, 118.8, 111.2, 110.2, 66.9, 55.3, 52.2, 50.8, 41.4, 28.5, 24.6, 22.6, 21.9.

### Cbz-_L_-Asn(Trt)-_L_-Leu-OMe (3-14)

Colorless solid; yield: 75%. ^1^H NMR (400 MHz, CDCl_3_) δ = 7.37–7.17 (m, 20H), 6.93 (s, 1H), 6.41 (d, *J* = 7.2 Hz, 1H), 5.14–5.07 (m, 2H), 4.57–4.53 (m, 1H), 4.47–4.42 (m, 1H), 3.70 (s, 3H), 3.07–3.03 (m, 1H), 2.69–2.64 (m, 1H), 1.58–1.48 (m, 2H), 1.43–1.38 (m, 1H), 0.87–0.84 (m, 6H); ^13^C NMR (100 MHz, CDCl_3_) δ = 172.8, 170.9, 170.4, 156.3, 144.2, 136.1, 128.6, 128.5, 128.1, 127.9, 127.0, 70.8, 66.9, 52.1, 51.1, 50.9, 40.6, 38.2, 24.7, 22.6, 21.7.

### Cbz-_L_-Phe-_L_-Leu-OMe (3-15)

Colorless solid; yield: 84%. ^1^H NMR (400 MHz, CDCl_3_) δ = 7.38–7.19 (m, 11H), 6.25 (d, *J* = 7.6 Hz, 1H), 5.33 (d, *J* = 7.2 Hz, 1H), 5.13–5.06 (m, 2H), 4.50–4.43 (m, 2H), 3.69 (s, 3H), 3.15–3.02 (m, 2H), 1.80 (s, 1H), 1.39–1.25 (m, 1H), 1.11–1.00 (m, 1H), 0.87 (t, *J* = 7.4 Hz, 3H), 0.79 (d, *J* = 6.8 Hz, 3H); ^13^C NMR (100 MHz, CDCl_3_) δ = 171.7, 170.6, 155.9, 136.2, 136.1, 129.3, 128.6, 128.5, 128.1, 127.9, 126.9, 67.0, 56.5, 56.1, 52.0, 38.3, 37.7, 25.0, 15.2, 11.4.

### Cbz-_L_-Phe-_L_-Tyr(Bzl)-OMe (3-16)

Colorless solid; yield: 73%. ^1^H NMR (400 MHz, CDCl_3_) δ = 7.42–7.16 (m, 19H), 6.88–6.81 (m, 4H), 6.17 (d, *J* = 7.2 Hz, 1H), 5.23 (d, *J* = 8.8 Hz, 1H), 5.08 (s, 2H), 4.99 (s, 2H), 4.75–4.71 (m, 1H), 4.40 (d, *J* = 6.4 Hz, 1H), 3.67 (s, 3H), 3.05–2.92 (m, 4H); ^13^C NMR (101 MHz, CDCl_3_) δ = 171.4, 170.3, 157.9, 155.8, 136.9, 136.2, 136.1, 130.2, 129.3, 128.6, 128.5, 128.5, 128.1, 127.9, 127.9, 127.7, 127.4, 127.0, 114.8, 69.9, 67.0, 55.9, 53.4, 52.2, 38.3, 37.0; HRMS(ESI): *m*/*z* calcd. for C_34_H_34_N_2_O_6_ [M+H]^+^: 567.2490, found 567.2486.

### Cbz-_L_-Phe-_L_-Ser-OMe (3-17)

Colorless solid; yield: 60%. ^1^H NMR (400 MHz, CDCl_3_) δ = 7.34–7.25 (m, 10H), 7.20–7.18 (m, 2H), 6.68 (s, 1H), 5.25 (s, 1H), 5.08 (s, 2H), 4.57–4.55 (m, 1H), 4.43–4.38 (m, 1H), 3.91–3.88 (m, 2H), 3.75 (s, 3H), 3.10 (d, *J* = 6.8 Hz, 2H), 2.52 (s, 1H); ^13^C NMR (100 MHz, CDCl_3_) δ = 171.6, 170.5, 156.3, 136.2, 136.0, 129.3, 128.5, 128.4, 128.1, 127.9, 126.9, 67.0, 62.6, 56.1, 54.7, 52.6, 38.4.

### Cbz-_L_-Phe-_L_-Thr-OMe (3-18)

Colorless solid; yield: 42%. ^1^H NMR (400 MHz, CDCl_3_) δ = 7.34–7.18 (m, 10H), 6.74 (d, *J* = 8.4 Hz, 1H), 5.40 (d, *J* = 8.0 Hz, 1H), 5.06 (s, 2H), 4.57–4.48 (m, 2H), 4.29–4.25 (m, 1H), 3.71 (s, 3H), 3.15–3.04 (m, 2H), 2.56 (d, *J* = 4.8 Hz, 1H), 1.12 (d, *J* = 6.4 Hz, 3H); ^13^C NMR (100 MHz, CDCl_3_) δ = 171.7, 171.1, 156.1, 136.2, 136.0, 129.3, 128.5, 128.4, 128.1, 127.9, 126.9, 68.1, 67.0, 57.4, 56.1, 52.5, 38.2, 19.7.

### Cbz-_L_-Phe-_L_-Tyr-OMe (3-19)

Colorless solid; yield: 84%. ^1^H NMR (400 MHz, CDCl_3_) δ = 7.34–7.23 (m, 8H), 7.14 (d, *J* = 6.8 Hz, 2H), 6.81 (d, *J* = 8.0 Hz, 2H), 6.64 (d, *J* = 8.4 Hz, 2H), 6.36 (s, 1H), 5.88 (s, 1H), 5.32 (d, *J* = 7.2 Hz, 1H), 5.06 (s, 2H), 4.77–4.73 (m, 1H), 4.42 (d, *J* = 6.8 Hz, 1H), 3.67 (s, 3H), 3.03–2.89 (m, 4H); ^13^C NMR (100 MHz, CDCl_3_) δ = 171.5, 170.8, 156.0, 155.3, 136.1, 130.3, 129.3, 128.6, 128.5, 128.2, 128.0, 127.0, 126.8, 115.5, 67.1, 55.9, 53.5, 52.3, 38.3, 37.0.

### Boc-_L_-Val-_L_-Val-OMe (3-20)

Colorless solid; yield: 75%. ^1^H NMR (400 MHz, CDCl_3_) δ = 6.36 (d, *J* = 6.8 Hz, 1H), 5.04 (d, *J* = 8.4 Hz, 1H), 4.54 (dd, *J* = 8.8, 4.8 Hz, 1H), 3.92–3.88 (m, 1H), 3.73 (s, 3H), 2.22–2.12 (m, 2H), 1.44 (s, 9H), 0.97–0.90 (m, 12H); ^13^C NMR (100 MHz, CDCl_3_) δ = 172.1, 171.7, 155.8, 79.6, 59.9, 57.0, 51.9, 31.0, 30.6, 28.2, 19.1, 18.8, 17.9, 17.7.

### Cbz-_L_-Val-_L_-Glu(OEt)-OEt (3-21)

Colorless solid; yield: 70%. ^1^H NMR (400 MHz, CDCl_3_) δ = 7.36–7.30 (m, 5H), 6.64 (d, *J* = 7.2 Hz, 1H), 5.38 (d, *J* = 7.6 Hz, 1H), 5.14–5.07 (m, 2H), 4.60–4.54 (m, 1H), 4.22–4.10 (m, 4H), 4.05–4.01 (m, 1H), 2.46–2.31 (m, 2H), 2.24–1.96 (m, 3H), 1.29–1.22 (m, 6H), 0.95 (dd, *J* = 16.4, 6.8 Hz, 6H); ^13^C NMR (101 MHz, CDCl_3_) δ = 172.9, 171.4, 171.2, 156.3, 136.2, 128.5, 128.1, 128.0, 66.9, 61.6, 60.8, 60.2, 51.8, 31.2, 30.2, 26.9, 19.0, 17.7, 14.1, 14.0.

### Boc-_L_-Val-_L_-Pro-OMe (3-22)

Colorless oily liquid; yield: 46%. ^1^H NMR (400 MHz, CDCl_3_) δ = 5.19 (d, *J* = 9.2 Hz, 1H), 4.52–4.49 (m, 1H), 4.28–4.24 (m, 1H), 3.78–3.61 (m, 5H), 2.25–2.17 (m, 1H), 2.06–1.91 (m, 4H), 1.40 (s, 9H), 1.01 (d, *J* = 6.8 Hz, 3H), 0.92 (d, *J* = 6.8 Hz, 3H); ^13^C NMR (100 MHz, CDCl_3_) δ = 172.4, 171.2, 155.8, 79.4, 58.7, 56.8, 52.1, 47.1, 31.3, 29.0, 28.3, 24.9, 19.2, 17.3.

### Boc-_L_- Pro-_L_-Ala-OMe (3-23)

Colorless solid; yield: 84%. ^1^H NMR (400 MHz, CDCl_3_) δ = 7.31 (s, 0.64H), 6.56 (s, 0.35H), 4.54 (s, 1H), 4.29–4.20 (m, 1H), 3.72 (s, 3H), 3.54–3.32 (m, 2H), 2.30–2.12 (m, 2H), 1.86 (s, 2H), 1.45 (s, 9H), 1.37 (d, *J* = 7.2 Hz, 3H); ^13^C NMR (100 MHz, CDCl_3_) δ = 173.1, 172.1, 171.6, 155.6, 154.5, 80.6, 80.3, 60.9, 59.7, 52.3, 47.9, 47.0, 30.8, 28.2, 24.5, 23.6, 18.5, 18.1.

### Boc-_L_- Pro-_L_-Leu-OMe (3-24)

Colorless solid; yield: 93%. ^1^H NMR (400 MHz, CDCl_3_) δ = 7.36 (s, 0.43H), 6.39 (s, 0.36H), 4.60–4.51 (m, 1H), 4.31–4.23 (m, 1H), 3.70 (s, 3H), 3.46–3.31 (m, 2H), 2.35–2.12 (m, 2H), 1.86 (s, 2H), 1.63–1.45 (m, 12H), 0.90 (s, 6H); ^13^C NMR (100 MHz, CDCl_3_) δ = 173.0, 172.3, 171.6, 155.8, 154.6, 130.8, 128.8, 80.3, 61.0, 59.5, 52.1, 50.7, 46.9, 41.3, 31.8, 31.4, 30.8, 30.1, 29.6, 29.6, 29.3, 28.2, 27.5, 24.8, 23.9, 22.8, 22.6, 21.7, 19.1, 14.0.

### Boc-_L_- Leu-_L_-Pro-OMe (3-25)

Colorless oily liquid; yield: 63%. ^1^H NMR (400 MHz, CDCl_3_) δ = 5.11 (d, *J* = 8.8 Hz, 1H), 4.53–4.44 (m, 2H), 3.79–3.71 (m, 4H), 3.62–3.55 (m, 1H), 2.24–2.17 (m, 1H), 2.07–1.94 (m, 3H), 1.50–1.41 (m, 12H), 1.00–0.94 (m, 7H); ^13^C NMR (100 MHz, CDCl_3_) δ = 172.4, 171.8, 155.6, 79.4, 58.6, 52.1, 50.2, 46.6, 41.9, 28.9, 28.3, 24.8, 24.5, 23.3, 21.7.

### Cbz-Gly-_L_-Pro-OMe (3-26)

Colorless oily liquid; yield: 83%. ^1^H NMR (400 MHz, CDCl_3_) δ = 7.36–7.26 (m, 5H), 5.68 (s, 1H), 5.11 (s, 2H), 4.54–4.51 (m, 1H), 4.08–3.97 (m, 2H), 3.73 (s, 3H), 3.64–3.57 (m, 1H), 3.49–3.44 (m, 1H), 2.24–2.00 (m, 4H); ^13^C NMR (100 MHz, CDCl_3_) δ = 172.2, 166.9, 156.1, 136.4, 128.4, 127.9, 127.8, 66.7, 58.8, 52.2, 45.8, 43.2, 28.9, 24.5, 22.1.

### Cbz-_L_-Ala-Aib-OMe (6-1)

Colorless oily liquid; yield: 62%. ^1^H NMR (400 MHz, CDCl_3_) δ = 7.35–7.31 (m, 5H), 6.71 (s, 1H), 5.43 (d, *J* = 5.2 Hz, 1H), 5.10 (s, 2H), 4.25–4.21 (m, 1H), 3.71 (s, 3H), 1.57–1.51 (m, 6H), 1.36 (d, *J* = 7.2 Hz, 3H); ^13^C NMR (100 MHz, CDCl_3_) δ = 174.7, 171.5, 156.0, 136.2, 128.5, 128.1, 127.9, 66.9, 56.4, 52.6, 50.4, 24.7, 24.6, 18.4.

### Boc-Gly-Aib-OMe (6-2)

Colorless solid; yield: 72%. ^1^H NMR (400 MHz, CDCl_3_) δ = 6.70 (s, 1H), 5.20 (s, 1H), 3.76–3.73 (m, 5H), 1.55 (s, 6H), 1.45 (s, 9H); ^13^C NMR (100 MHz, CDCl_3_) δ = 174.8, 168.8, 156.1, 80.0, 56.4, 52.6, 44.4, 28.2, 24.7.

### Boc-_L_-Leu-Aib-OMe (6-3)

Colorless solid; yield: 67%. ^1^H NMR (400 MHz, CDCl_3_) δ = 6.65 (s, 1H), 4.84 (s, 1H), 4.05 (s, 1H), 3.72 (s, 3H), 1.69–1.64 (m, 3H), 1.57–1.50 (m, 6H), 1.45 (s, 9H), 0.95–0.92 (m, 6H); ^13^C NMR (100 MHz, CDCl_3_) δ = 174.7, 171.8, 155.8, 79.9, 56.2, 52.9, 52.4, 40.8, 28.2, 24.7, 24.6, 22.8, 22.0; HRMS(ESI): *m*/*z* calcd. for C_16_H_30_N_2_O_5_ [M+H]^+^: 331.2227, found 331.2227.

### Boc-_L_-Tyr-Aib-OMe (6-4)

Light yellow oily liquid; yield:70%. ^1^H NMR (400 MHz, CDCl_3_) δ = 7.07 (d, *J* = 8.0 Hz, 2H), 6.75 (d, *J* = 8.4 Hz, 2H), 6.30 (s, 1H), 5.65 (s, 1H), 5.10 (s, 1H), 4.22 (s, 1H), 3.72 (s, 3H), 3.04–2.88 (m, 2H), 1.46–1.43 (m, 15H); ^13^C NMR (100 MHz, CDCl_3_) δ = 174.6, 170.7, 155.6, 155.3, 130.5, 127.9, 115.5, 80.3, 56.4, 55.9, 52.6, 37.5, 28.2, 24.7, 24.5.

### Boc-_L_-Pro-Aib-OMe (6-5)

Colorless solid; yield: 81%. ^1^H NMR (400 MHz, CDCl_3_) δ = 7.47 (s, 0.44H), 6.48 (s, 0.45H), 4.26–4.14 (m, 1H), 3.71 (s, 3H), 3.47–3.32 (m, 2H), 2.33–1.85 (m, 4H), 1.53–1.46 (m, 15H); ^13^C NMR (100 MHz, CDCl_3_) δ = 174.6, 171.7, 171.1, 155.7, 154.4, 80.2, 61.0, 59.7, 56.0, 52.3, 46.8, 30.7, 28.2, 24.9, 24.6, 23.6.

### Cbz-_L_-Trp-Aib-OMe (6-6)

Colorless solid; yield: 77%. ^1^H NMR (400 MHz, CDCl_3_) δ = 8.28 (s, 1H), 7.71 (d, *J* = 6.0 Hz, 1H), 7.37–7.30 (m, 6H), 7.22–7.10 (m, 3H), 6.20 (s, 1H), 5.56 (d, *J* = 6.0 Hz, 1H), 5.12 (s, 2H), 4.51 (s, 1H), 3.67 (s, 3H), 3.36–3.33 (m, 1H), 3.17–3.11 (m, 1H), 1.35 (d, *J* = 5.6 Hz, 6H); ^13^C NMR (100 MHz, CDCl_3_) δ = 174.5, 170.3, 156.0, 136.2, 128.5, 128.1, 128.0, 127.3, 123.5, 122.3, 119.8, 118.9, 111.2, 110.4, 66.9, 56.3, 55.3, 52.5, 28.4, 24.7.

### Cbz-_L_-Phe-Aib-OMe (6-7)

Colorless solid; yield: 59%. ^1^H NMR (400 MHz, CDCl_3_) δ = 7.37–7.22 (m, 10H), 6.21 (s, 1H), 5.43 (d, *J* = 4.8 Hz, 1H), 5.09 (s, 2H), 4.37 (d, *J* = 6.4 Hz, 1H), 3.69 (s, 3H), 3.15–3.10 (m, 1H), 3.02–2.96 (m, 1H), 1.41 (d, *J* = 10.4 Hz, 6H); ^13^C NMR (100 MHz, CDCl_3_) δ = 174.4, 169.8, 155.9, 136.5, 136.2, 129.4, 128.6, 128.5, 128.1, 127.9, 127.0, 66.9, 56.4, 56.2, 52.6, 38.6, 24.5, 24.4.

### Boc-_L_-Ala-Aib-OMe (6-8)

Colorless oily liquid; yield: 79%. ^1^H NMR (400 MHz, CDCl_3_) δ = 6.77 (s, 1H), 5.04 (s, 1H), 4.12 (s, 1H), 3.71 (s, 3H), 1.52 (d, *J* = 6.0 Hz, 6H), 1.44 (s, 9H), 1.32 (d, *J* = 6.8 Hz, 3H); ^13^C NMR (100 MHz, CDCl_3_) δ = 174.8, 171.8, 155.7, 80.0, 56.3, 52.6, 49.9, 28.3, 24.8, 24.7, 17.8; HRMS(ESI): *m*/*z* calcd. for C_13_H_24_N_2_O_5_ [M+H]^+^: 289.1758, found 289.1759.

### Cbz-_L_-Met-Aib-OMe (6-9)

Light yellow oily liquid; yield: 63%. ^1^H NMR (400 MHz, CDCl_3_) δ = 7.35–7.29 (m, 5H), 6.75 (s, 1H), 5.58 (d, *J* = 7.6 Hz, 1H), 5.10 (s, 2H), 4.36–4.31 (m, 1H), 3.71 (s, 3H), 2.64–2.52 (m, 2H), 2.10–1.93 (m, 5H), 1.51 (d, *J* = 5.2 Hz, 6H); ^13^C NMR (100 MHz, CDCl_3_) δ = 174.4, 170.2, 156.0, 136.1, 128.5, 128.1, 128.0, 67.0, 56.4, 53.6, 52.6, 31.5, 29.8, 24.9, 24.5, 15.0.

### Cbz-_L_-Ser(*t*Bu)-Aib-OMe (6-10)

Colorless oily liquid; yield: 68%. ^1^H NMR (400 MHz, CDCl_3_) δ = 7.36–7.26 (m, 6H), 5.73 (s, 1H), 5.15–5.08 (m, 2H), 4.18 (s, 1H), 3.80–3.72 (m, 4H), 3.34 (d, *J* = 8.4 Hz, 1H), 1.54 (d, *J* = 8.0 Hz, 6H), 1.21 (s, 9H); ^13^C NMR (100 MHz, CDCl_3_) δ = 174.6, 169.4, 155.9, 136.2, 128.4, 128.1, 128.0, 74.1, 66.8, 61.6, 56.4, 54.1, 52.5, 27.3, 24.8, 24.5; HRMS(ESI): *m*/*z* calcd. for C_20_H_30_N_2_O_6_ [M+H]^+^: 395.2177, found 395.2182.

### Cbz-_L_-Val-Aib-OMe (6-11)

Colorless solid; yield: 46%. ^1^H NMR (400 MHz, CDCl_3_) δ = 7.35–7.28 (m, 5H), 6.50 (s, 1H), 5.39 (d, *J* = 7.2 Hz, 1H), 5.11 (s, 2H), 3.98–3.94 (m, 1H), 3.71 (s, 3H), 2.14–2.09 (m, 1H), 1.53 (s, 6H), 0.94 (dd, *J* = 16.8, 6.8 Hz, 6H); ^13^C NMR (100 MHz, CDCl_3_) δ = 174.7, 170.3, 156.4, 136.2, 128.5, 128.1, 128.0, 67.0, 60.2, 56.5, 52.6, 31.1, 24.8, 24.5, 19.0, 17.7.

### Cbz-_L_-NMePhe-Aib-OMe (6-12)

Colorless oily liquid; yield: 72%. ^1^H NMR (400 MHz, CDCl_3_) δ = 7.36–7.11 (m, 10H), 6.50 (s, 0.6H), 6.22 (s, 0.3H), 5.15–5.07 (m, 1.7H), 4.94–4.78 (m, 1.5H), 3.72–3.67 (m, 3H), 3.32–3.27 (m, 1H), 2.97–2.86 (m, 4H), 1.48–1.42 (m, 6H); ^13^C NMR (100 MHz, CDCl_3_) δ = 174.4, 169.3, 169.0, 157.2, 155.9, 137.4, 137.2, 136.5, 136.0, 128.9, 128.4, 128.1, 127.9, 127.5, 126.5, 67.6, 67.3, 61.0, 59.9, 56.4, 56.2, 52.5, 52.4, 33.9, 33.7, 30.8, 30.2, 25.0, 24.7, 24.4.

### Cbz-_L_-NMePhe-_L_-Val-OMe (6-13)

Colorless oily liquid; yield: 83%. ^1^H NMR (400 MHz, CDCl_3_) δ = 7.35–7.12 (m, 10H), 6.53 (d, *J* = 8.8 Hz, 0.6H), 6.23 (s, 0.3H), 5.16–5.07 (m, 1.8H), 4.98–4.95 (m, 1H), 4.86 (s, 0.3H), 4.52–4.49 (m, 1H), 3.71 (s, 3H), 3.36–3.27 (m, 1H), 3.07–2.50 (m, 4H), 2.16–2.07 (m, 1H), 0.86–0.79 (m, 6H); ^13^C NMR (100 MHz, CDCl_3_) δ = 171.8, 170.0, 169.7, 157.1, 155.9, 137.3, 137.1, 136.3, 135.9, 128.8, 128.4, 128.1, 127.9, 127.6, 126.5, 67.7, 67.4, 61.0, 60.1, 56.9, 52.0, 34.1, 33.8, 31.3, 31.0, 30.6, 18.8, 17.6, 17.4; HRMS(ESI): *m*/*z* calcd. for C_24_H_30_N_2_O_5_ [M+H]^+^: 427.2227 found 427.2228.

### Cbz-_L_-NMePhe-Gly-OMe (6-14)

Colorless oily liquid; yield: 90%. ^1^H NMR (400 MHz, CDCl_3_) δ = 7.34–7.13 (m, 10H), 6.58 (s, 0.7H), 6.27 (s, 0.3H), 5.14–4.87 (m, 3H), 4.15–4.04 (m, 1H), 3.92–3.87 (m, 1H), 3.73 (s, 3H), 3.38–3.33 (m, 1H), 3.03–2.87 (m, 4H); ^13^C NMR (100 MHz, CDCl_3_) δ = 170.5, 169.9, 157.2, 137.1, 136.3, 128.8, 128.4, 128.0, 127.9, 127.4, 126.5, 67.6, 67.3, 61.0, 59.9, 52.1, 41.0, 34.0, 33.8, 31.1, 30.4.

### Cbz-_L_-NMePhe-_L_-Ala-OMe (6-15)

Colorless oily liquid; yield: 81%. ^1^H NMR (400 MHz, CDCl_3_) δ = 7.35–7.12 (m, 10H), 6.53 (s, 0.6H), 6.26 (s, 0.4H), 5.10–5.05 (m, 1.8H), 4.95–4.93 (m, 1H), 4.80 (s, 0.4H), 4.56–4.51 (m, 1H), 3.72 (s, 3H), 3.34–3.31 (m, 1H), 3.05–2.84 (m, 4H), 1.35–1.33 (m, 3H); ^13^C NMR (100 MHz, CDCl_3_) δ = 172.8, 169.7, 169.3, 156.9, 155.9, 137.3, 137.1, 136.4, 135.9, 128.8, 128.4, 128.0, 127.9, 127.5, 126.5, 67.6, 67.3, 61.0, 60.1, 52.3, 48.0, 34.0, 31.3, 30.6, 18.0; HRMS(ESI): *m*/*z* calcd. for C_22_H_26_N_2_O_5_ [M+H]^+^: 399.1914 found 399.1914.

### Cbz-_L_-NMePhe-_L_-Ile-OMe (6-16)

Colorless oily liquid; yield: 86%. ^1^H NMR (400 MHz, CDCl_3_) δ = 7.36–7.10 (m, 10H), 6.55 (d, *J* = 8.0 Hz, 0.6H), 6.24 (d, *J* = 9.2 Hz, 0.3H), 5.15–5.07 (m, 1.7H), 4.98–4.94 (m, 1H), 4.84 (s, 0.3H), 4.56–4.53 (m, 1H), 3.70 (s, 3H), 3.62–3.28 (m, 1H), 3.07–2.85 (m, 4H), 1.86 (s, 1H), 1.40–1.29 (m, 1H), 1.10–1.01 (m, 1H), 0.89–0.82 (m, 6H); ^13^C NMR (100 MHz, CDCl_3_) δ = 171.7, 169.9, 169.6, 157.1, 155.9, 137.3, 137.1, 136.3, 135.9, 128.8, 128.4, 128.0, 127.9, 127.6, 126.5, 67.7, 67.4, 61.1, 60.2, 56.3, 51.9, 37.6, 34.1, 33.8, 31.3, 30.6, 24.9, 15.3, 11.4.

### Cbz-_L_-NMePhe-_L_-His(Trt)-OMe (6-17)

Colorless oily liquid; yield: 87%. ^1^H NMR (400 MHz, CDCl_3_) δ = 7.32–7.07 (m, 27H), 6.53 (d, *J* = 4.0 Hz, 1H), 5.11–4.87 (m, 3H), 4.75 (d, *J* = 3.6 Hz, 1H), 3.59 (s, 3H), 3.44–3.39 (m, 1H), 3.15–2.88 (m, 3H), 2.81 (d, *J* = 14.0 Hz, 3H); ^13^C NMR (100 MHz, CDCl_3_) δ = 171.3, 170.1, 169.9, 156.6, 155.8, 142.1, 138.6, 138.4, 137.6, 137.5, 136.5, 136.2, 129.5, 128.8, 128.7, 128.3, 128.2, 128.2, 127.9, 127.6, 127.6, 127.5, 127.2, 126.3, 126.2, 119.3, 119.2, 75.1, 67.1, 66.9, 61.0, 60.4, 52.6, 51.9, 33.9, 31.2, 30.9, 29.5, 29.1.

### Cbz-_L_-NMePhe-_L_-Tyr(Bzl)-OMe (6-18)

Colorless oily liquid; yield: 90%. ^1^H NMR (400 MHz, CDCl_3_) δ = 7.40–7.07 (m, 15H), 6.96–6.80 (m, 4H), 6.45 (d, *J* = 7.6 Hz, 0.6H), 6.14 (d, *J* = 6.0 Hz, 0.3H), 5.12–4.89 (m, 5H), 4.78–4.77 (m, 1H), 3.71 (s, 3H), 3.28–3.23 (m, 1H), 3.14–3.04 (m, 1H), 2.96–2.86 (m, 2H), 2.64–2.59 (m, 3H); ^13^C NMR (100 MHz, CDCl_3_) δ = 171.5, 169.6, 169.3, 157.8, 157.6, 156.9, 155.6, 137.1, 137.0, 136.8, 136.2, 135.9, 130.0, 128.7, 128.4, 128.3, 128.0, 127.9, 127.8, 127.5, 127.2, 126.4, 115.0, 114.7, 69.7, 67.5, 67.3, 60.5, 59.6, 53.0, 52.2, 36.9, 36.7, 33.8, 33.6, 30.6, 30.0; HRMS(ESI): *m*/*z* calcd. for C_35_H_36_N_2_O_6_ [M+H]^+^: 581.2646 found 581.2642.

### Cbz-_L_-NMePhe-_L_-Cys(Trt)-OMe (6-19)

Colorless oily liquid; yield: 80%. 1H NMR (400 MHz, CDCl3) δ = 7.33–7.01 (m, 25H), 6.52 (d, J = 6.8 Hz, 0.6H), 6.22 (d, *J* = 6.8 Hz, 0.4H), 5.05–4.73 (m, 3H), 4.38–4.34 (m, 1H), 3.59 (s, 3H), 3.23–3.18 (m, 1H), 2.92–2.67 (m, 4H), 2.60–2.56 (m, 1H), 2.47–2.42 (m, 1H); 13C NMR (100 MHz, CDCl3) δ = 170.3, 169.7, 169.5, 157.1, 155.8, 144.1, 137.2, 137.0, 136.2, 135.9, 129.3, 128.8, 128.4, 128.3, 127.9, 127.4, 127.1, 126.7, 126.5, 67.6, 67.4, 66.7, 66.6, 60.7, 59.6, 52.6, 52.4, 51.3, 34.0, 33.6, 33.5, 33.5, 31.1, 30.3; HRMS(ESI): m/z calcd. for C41H40N2O5S [M+H]+: 695.2550 found 695.2538.

### Cbz-_L_-NMePhe-_L_-Lys(Z)-OMe (6-20)

Colorless oily liquid; yield: 88%. ^1^H NMR (400 MHz, CDCl_3_) δ = 7.33–7.10 (m, 16H), 6.56 (d, *J* = 7.2 Hz, 0.6H), 6.30 (d, 0.3H), 5.08–4.80 (m, 6H), 4.56–4.51 (m, 1H), 3.71 (s, 3H), 3.32–2.81 (m, 7H), 1.82–1.24 (m, 6H); ^13^C NMR (100 MHz, CDCl_3_) δ = 172.2, 170.1, 169.7, 156.9, 156.4, 137.2, 137.0, 136.5, 136.3, 128.8, 128.4, 127.9, 127.5, 127.1, 126.5, 67.3, 66.4, 60.9, 60.5, 52.4, 52.3, 51.9, 51.8, 40.4, 40.3, 34.0, 31.7, 31.1, 29.3, 29.0, 22.3, 22.1; HRMS(ESI): *m*/*z* calcd. for C_33_H_39_N_3_O_7_ [M+H]^+^: 590.2861 found 590.2862.

### Cbz-_L_-NMePhe-_L_-Glu(OEt)-OEt (6-21)

Colorless oily liquid; yield: 82%. ^1^H NMR (400 MHz, CDCl_3_) δ = 7.33–7.08 (m, 10H), 6.71–6.61 (m, 1H), 5.08–5.02 (m, 1.6H), 4.95–4.89 (m, 1H), 4.78 (s, 0.4H), 4.52–4.51 (m, 1H), 4.19–4.06 (m, 4H), 3.33–3.28 (m, 1H), 3.08–2.82 (m, 4H), 2.31–2.21 (m, 3H), 1.96–1.90 (m, 1H), 1.26–1.19 (m, 6H); ^13^C NMR (100 MHz, CDCl_3_) δ = 172.6, 172.5, 171.2, 170.1, 169.9, 156.8, 155.8, 137.3, 137.0, 136.3, 135.9, 128.8, 128.4, 128.0, 127.9, 127.5, 126.5, 67.5, 67.3, 61.4, 61.2, 60.7, 60.5, 60.4, 51.9, 51.6, 34.1, 33.9, 31.5, 30.9, 30.1, 30.0, 27.0, 26.7, 14.0, 14.0; HRMS(ESI): *m*/*z* calcd. for C_27_H_34_N_2_O_7_ [M+H]^+^: 499.2439 found 499.2439.

### Cbz-_L_-NMePhe-_L_-Tyr-OMe (6-22)

Colorless oily liquid; yield: 91%. ^1^H NMR (400 MHz, CDCl_3_) δ = 7.34–7.06 (m, 10H), 6.88–6.59 (m, 5H), 6.40 (s, 0.4H), 6.22–6.14 (m, 1H), 5.13–4.78 (m, 4H), 3.73–3.71 (m, 3H), 3.28–2.83 (m, 4H), 2.64–2.63 (m, 3H); ^13^C NMR (100 MHz, CDCl_3_) δ = 171.9, 171.7, 170.0, 169.6, 157.1, 155.3, 137.0, 136.7, 136.2, 135.7, 130.1, 128.8, 128.6, 128.5, 128.1, 128.0, 127.6, 127.0, 126.6, 67.8, 67.5, 60.7, 59.9, 53.2, 52.4, 37.1, 36.8, 33.8, 30.9, 30.3; HRMS(ESI): *m*/*z* calcd. for C_28_H_30_N_2_O6 [M+H]^+^: 491.2177 found 491.2180.

### General Procedure for Fmoc-SPPS Mediated by IBA-OBz

All Fmoc-protected amino acids and 2-chlorotrityl chloride resin (2-Cl-Trt-Cl resin, 0.98 mmol/g) were purchased and used without further purification. All peptides were synthesized using 200 mg resin in solid-phase peptide synthesis vessel. The synthetic route included immobilized the C terminal amino acid onto resin, deprotection, coupling, cleavage from resin, and purification of peptides.

### Immobilized the C-Terminal Amino Acid Onto Resin

To a 5 mL round-bottom flask was added 2-Cl-Trt-Cl resin (0.196 mmol, 200 mg) and C-terminal Fmoc-amino acid (Fmoc-AA_1_-OH, 0.6 mmol), then DCM and DMF (2 mL, v:v = 1:1) were added and the mixture was stirred at room temperature. Then DIPEA (0.1 mL) was added and the mixture was stirred at room temperature for 4 h. Afterward, the mixture was filtered and washed with MeOH (3 × 5 mL) and DCM (3 × 5 mL) in sequence. The resin was used for next step without any purification.

### Blocked the Reaction Sites

To a 5 mL round-bottom flask was added the resin from last step and MeOH/DIPEA/DCM (2 mL, v:v:v = 1:2:7), the mixture was stirred at room temperature for 4 h. Afterward, the mixture was filtered and washed with MeOH (3 × 5 mL) and DCM (3 × 5 mL) in sequence. The resin was used for next step without any purification.

### Deprotection of Fmoc

To a solid-phase peptide synthesis vessel was added the resin from last step and 20% piperidine/DMF (2 mL), the tube was capped and the mixture was shaken with air bubbling from the bottom of the reaction vessel by using air-pump at room temperature for 30 min. The mixture was filtered and washed with MeOH (3 × 5 mL) and DCM (3 × 5 mL) in sequence. The resin was used for next step without any purification.

### Coupling

To a solid phase peptide synthesis vessel was added the resin from last step, Fmoc-AA_2_-OH (0.6 mmol) and DMF (2 mL), then the mixture was shaken with air bubbling from the bottom of the reaction vessel by using air-pump. Afterward, TEA (0.6 mmol, 84 μL) was added. After 5 min, IBA-OBz (221 mg, 0.6 mmol) and (4-MeOC_6_H_4_)_3_P (216 mg, 0.6 mmol) was added. After 2 h, the mixture was filtered and washed with MeOH (3 × 5 mL) and DCM (3 × 5 mL) in sequence. The resin was used for next step without any purification. The peptide chain elongation was completed by repeating deprotection of Fmoc and peptide coupling reaction.

### Cleavage

The resin was treated with a solution of 0.5% TFA/DCM (2 mL) for 10 min. Then the mixture was filtered and washed with MeOH (3 × 5 mL) and DCM (3 × 5 mL) in sequence. The filtrate was evaporated under vacuum to afford the crude peptide as a oily liquid.

### Purification of Peptides

The crude peptide was purified via RP-HPLC. Analytical HPLC was performed using a J&K C18 (5 μm, 4.6 mm × 250 mm) analytical column. Linear gradients using A: H_2_O (0.1% TFA) and B: MeCN (0.1% TFA) were run at a flow rate of 0.8 mL·min^−1^. Preparative HPLC was performed using a Shimpack Prep-ODS C18 (15 μm, 20 mm × 250 mm) preparative column. Linear gradients using A: H_2_O (0.1% TFA) and B: MeCN (0.1% TFA) were run at a flow rate of 8.0 mL·min^−1^.

### H_2_N-Tyr-Gly-Gly-Phe-Leu-OH

Colorless solid, yield: 44%. QFT/ESI: m/z calcd. for C_28_H_37_N_5_O_7_ [M+H]^+^: 556.2693, found: 556.2768; for C_28_H_37_N_5_O_7_. [M+Na]^+^: 578.2585, found: 578.2549.

## Data Availability Statement

All datasets generated for this study are included in the article/Supplementary Material.

## Author Contributions

L-JQ completed SPPS and completed the draft. DL synthesized iodine(III) compounds and dipeptides. KZ completed computational studies. M-TZ guided computational studies. CZ directed the project and finalized the manuscript.

### Conflict of Interest

The authors declare that the research was conducted in the absence of any commercial or financial relationships that could be construed as a potential conflict of interest.
