# Supplementary material for: A Benziodoxole-Based Hypervalent Iodine(III) Compound Functioning as a Peptide Coupling Reagent
Source: Front Chem. 2020 Mar 18;8:183. doi: 10.3389/fchem.2020.00183 (PMC7093377; doi:10.3389/fchem.2020.00183)
Supplement: Supplementary file 1 [file Data_Sheet_1.pdf]

A Benziodoxolone-Based Hypervalent Iodine(III) Compound Functioning as a Peptide Coupling Reagent

(Supporting Information)

Li-Jun Qiu,[1‡] Dan Liu,[1‡] Ke Zheng,[1‡] Ming-Tao Zhang[2*], and Chi Zhang[1*]

[1]State Key Laboratory of Elemento-Organic Chemistry, Collaborative Innovation Center of Chemical Science and Engineering (Tianjin), College of Chemistry, Nankai University, Tianjin 300071, China.

[2]Computational Center for Molecular Science, Nankai University, Tianjin 300071, China.

These authors contributed equally to this work.

E-mail: zhangmt@nankai.edu.cn

E-mail: zhangchi@nankai.edu.cn

Single Crystallographic Structure of IBA-OBz 2

Mechanistic Studies 2

Density functional theory (DFT) calculations 3

^{1}H NMR, ^{13}C NMR of Products 40

Table of Contents

Single Crystallographic Structure of IBA-OBz

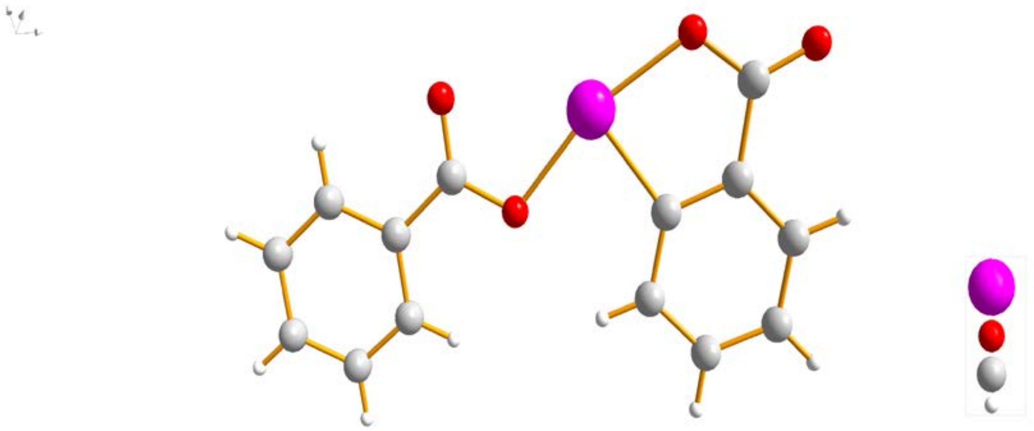

Figure S1. Single-crystal X-ray structure of IBA-OBz.

A single crystal of product IBA-OBz suitable for X-ray crystallographic analysis was grown from chloroform/diethyl ether at room temperature. The X-ray diffraction data was collected on Bruker SMART-1000 CCD diffractometer.

Table S1. Single Crystallographic Data of IBA-OBz

Compound	IBA-OBz	*c*, Å	19.938(4) Å
Formula	$C_{14}H_9I_1O_4$	α, deg	90
Molecular weight	368.11	β, deg	93.20(3)°
Crystal system	Monoclinic	γ, deg	90
Space group	P 1 21/n 1 (14)	*V*, $Å^3$	1239.55(43) $Å^3$
Color	Colorless	*Z*	2
a, Å	6.5858(13) Å	ρ_{calc}, $g.cm^{-3}$	1.9724 g/cm^3
b, Å	9.4548(19) Å	CCDC number	1947276

Mechanistic Studies

A mixture of IBA-OBz (55.2 mg, 0.15 mmol) and DMAP (36.6 mg, 0.3 mmol) in DCE (5 mL) in a 10 mL rounded bottom flask was stirred under room temperature in air. Aliquots (one drop) were taken out by syringe at every 2 min and diluted with $CHCl_3$/MeOH prior before the injection into the mass spectrometer.

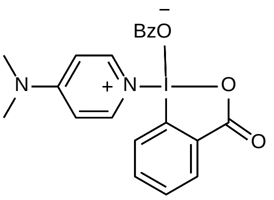

Exact Mass: 490.0390 (calc. for [**IM1**+H]$^+$: 491.0462)
Observed: m/z = 491.2082 ([**IM1**+H]$^+$)

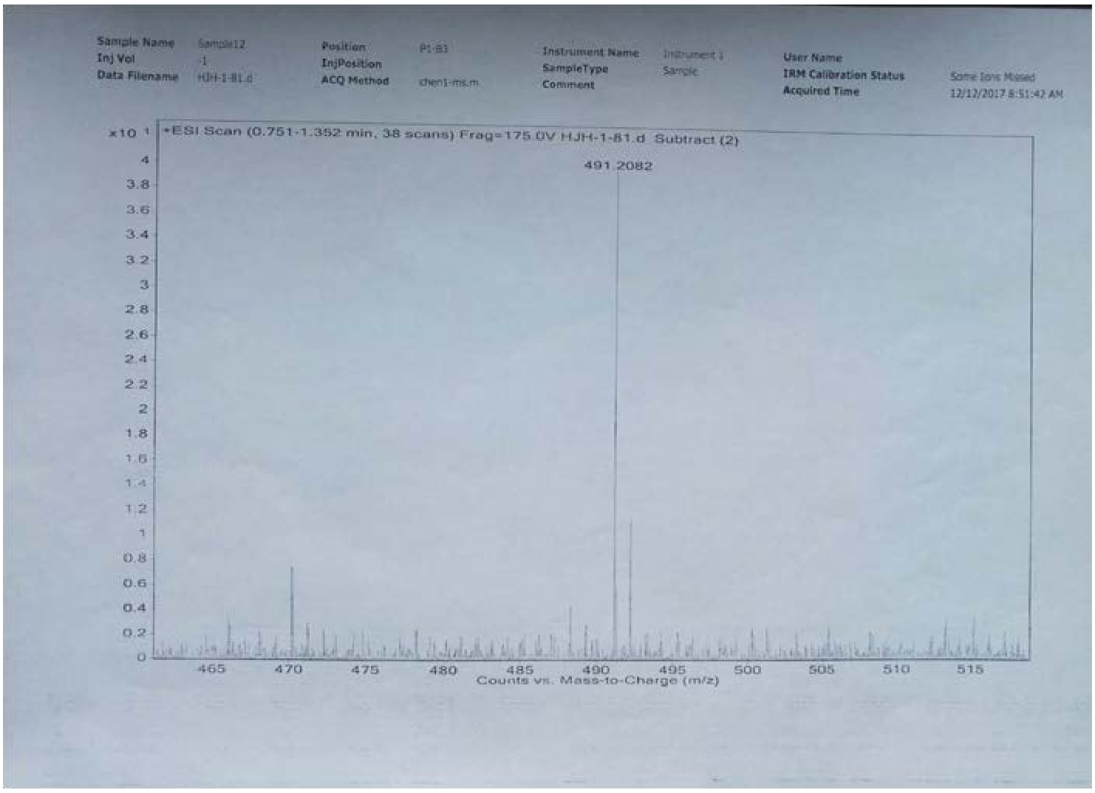

Figure S2. Mass spectrum of zwitterion **IM1**.

Density functional theory (DFT) calculations

SMD-M06-2X/[6-31G(d)+LANL2DZ (I)] calculated cartesian coordinates

I

C	-3.11408300	0.66899000	0.00217600
C	-3.88714100	1.82879700	0.00686400
C	-3.26015800	3.06943000	0.01157300
C	-1.86701300	3.15185200	0.01174700
C	-1.07598000	2.00147000	0.00695300
C	-1.73492000	0.78453300	0.00207200
H	-4.96831500	1.73391300	0.00681900
H	-3.85449400	3.97697600	0.01523900
H	-1.38129600	4.12232300	0.01567500
H	0.00427100	2.06423800	0.00729300
I	-0.79881200	-1.11539500	-0.00492700
C	-3.72813000	-0.69782500	-0.00210000

O	-4.92841500	-0.87846600	-0.00258900
O	-2.84123700	-1.67118900	-0.00498800
O	0.99242100	0.00880400	-0.00740300
C	2.04433600	-0.78368200	0.00465200
O	1.94443600	-2.00263200	0.01708100
C	3.35912900	-0.07721300	0.00144200
C	4.52326600	-0.84888800	0.01943300
C	3.43929400	1.31686100	-0.01961700
C	5.76680600	-0.22641400	0.01679300
H	4.43811500	-1.93055900	0.03541000
C	4.68532800	1.93650700	-0.02238600
H	2.52919800	1.90713900	-0.03413300
C	5.84765200	1.16607700	-0.00418500
H	6.67226300	-0.82474000	0.03075900
H	4.75061800	3.01980100	-0.03886500
H	6.81872500	1.65175500	-0.00638400

Zero-point correction= 0.201006 (Hartree/Particle)

Thermal correction to Energy= 0.217196

Thermal correction to Enthalpy= 0.218140

Thermal correction to Gibbs Free Energy= 0.153232

Sum of electronic and zero-point Energies= -850.563140

Sum of electronic and thermal Energies= -850.546950

Sum of electronic and thermal Enthalpies= -850.546006

Sum of electronic and thermal Free Energies= -850.610914

DMAP

C	-1.94751900	1.13166200	-0.00895300
C	-0.56432100	1.19851800	-0.00816600

C	0.18599800	0.00002500	0.00111200
C	-0.56431300	-1.19847200	0.00934300
C	-1.94751400	-1.13164500	0.00837600
N	-2.66432100	0.00000200	-0.00076500
H	-2.51855100	2.05858200	-0.01743700
H	-0.08053300	2.16739200	-0.01764200
H	-0.08050400	-2.16733600	0.01944500
H	-2.51853700	-2.05857600	0.01604000
N	1.54642200	-0.00002100	0.00218100
C	2.26951000	-1.25827700	-0.01624800
H	3.33875400	-1.05106500	-0.04931900
H	2.06010600	-1.85612100	0.87941500
H	2.00807200	-1.85591600	-0.89783600
C	2.26965800	1.25822800	0.01444500
H	2.06012200	1.85220100	-0.88384400
H	2.00849600	1.85971500	0.89341100
H	3.33887300	1.05102200	0.04839300

Zero-point correction= 0.164333 (Hartree/Particle)

Thermal correction to Energy= 0.172899

Thermal correction to Enthalpy= 0.173843

Thermal correction to Gibbs Free Energy= 0.129121

Sum of electronic and zero-point Energies= -381.932954

Sum of electronic and thermal Energies= -381.924387

Sum of electronic and thermal Enthalpies= -381.923443

Sum of electronic and thermal Free Energies= -381.968166

TS1

C	3.51483700	-1.03675900	0.60582400
C	4.39033000	-0.88698900	1.68029300

C	3.92594900	-0.31588200	2.85944700
C	2.59406600	0.09167100	2.96927500
C	1.70675100	-0.04895000	1.90127800
C	2.20776900	-0.60049400	0.73676200
H	5.41603300	-1.22611700	1.57329800
H	4.59747200	-0.19548500	3.70290700
H	2.23532800	0.52029500	3.89956100
H	0.66756100	0.24569000	1.98270300
I	1.08768700	-0.96268400	-1.00924600
C	3.91605800	-1.67910500	-0.67814500
O	5.03083200	-2.08815400	-0.91372800
O	2.92091400	-1.77618600	-1.55604800
O	-1.58398600	-1.13347600	-1.43895900
C	-1.78732800	-1.49348200	-0.25411400
O	-0.93993400	-1.39564700	0.67837300
C	-3.14369900	-2.07048900	0.09165000
C	-3.42411900	-2.47785400	1.39727800
C	-4.12305200	-2.19560900	-0.89573000
C	-4.67299800	-3.00650800	1.71403500
H	-2.65162400	-2.37453500	2.15249800
C	-5.37223700	-2.72401100	-0.58119000
H	-3.88810200	-1.87461600	-1.90538900
C	-5.64837400	-3.13021100	0.72453600
H	-4.88698300	-3.32302800	2.73063700
H	-6.13104400	-2.82062600	-1.35217000
H	-6.62240000	-3.54304000	0.97038400
C	0.68210600	2.21829900	-0.84226300
C	0.31877600	3.54690700	-0.77671600
C	-0.87840600	3.90706700	-0.11027000

C	-1.61682700	2.84023700	0.46052200
C	-1.15851600	1.54477700	0.34028200
N	-0.02593900	1.21753000	-0.30262400
H	1.59714400	1.93641300	-1.36084200
H	0.95433000	4.28856800	-1.24293900
H	-2.53895700	3.01731400	0.99910500
H	-1.70110000	0.72465800	0.79471300
N	-1.28969800	5.19233500	-0.02200500
C	-2.53144400	5.51360000	0.66243900
H	-2.70189200	6.58749200	0.59685800
H	-3.38210100	5.00013800	0.20022700
H	-2.48821400	5.23235400	1.72119600
C	-0.48371300	6.25264800	-0.60427300
H	0.51781300	6.27952000	-0.15951700
H	-0.37959500	6.12383300	-1.68792900
H	-0.96982200	7.20889800	-0.41549200

Zero-point correction= 0.366494 (Hartree/Particle)

Thermal correction to Energy= 0.392231

Thermal correction to Enthalpy= 0.393175

Thermal correction to Gibbs Free Energy= 0.306530

Sum of electronic and zero-point Energies= -1232.469276

Sum of electronic and thermal Energies= -1232.443539

Sum of electronic and thermal Enthalpies= -1232.442595

Sum of electronic and thermal Free Energies= -1232.529240

IM1

C	2.84561100	-2.69969700	-0.01237600
C	4.18768600	-3.07393900	-0.04577800
C	5.16898300	-2.09303600	-0.12022700

C	4.80319800	-0.74735300	-0.16543000
C	3.46123100	-0.36517100	-0.13070000
C	2.49516600	-1.35801400	-0.04814000
H	4.43603900	-4.13019100	-0.01610000
H	6.21738300	-2.37120200	-0.14779200
H	5.56823800	0.02006500	-0.23102700
H	3.20414800	0.68623600	-0.17444900
I	0.37588300	-1.08560400	0.01214800
C	1.74937000	-3.71674900	0.04557200
O	1.96245400	-4.91331700	0.08482600
O	0.54272500	-3.19410100	0.04331900
O	-2.24013400	-1.89869500	0.05263400
C	-2.68043800	-0.72209200	0.01372500
O	-1.96991600	0.31732100	-0.01867400
C	-4.18813500	-0.53841000	0.00719400
C	-4.73941000	0.74378400	-0.02056700
C	-5.03346400	-1.64920600	0.03104100
C	-6.12155600	0.91574600	-0.02387300
H	-4.06723100	1.59575500	-0.03863400
C	-6.41610000	-1.48104800	0.02694000
H	-4.58874100	-2.63903100	0.05303400
C	-6.96201100	-0.19751600	-0.00023000
H	-6.54532100	1.91571400	-0.04461100
H	-7.06924400	-2.34875500	0.04522200
H	-8.04007500	-0.06502100	-0.00274600
C	0.68518400	1.80691600	-1.13427500
C	0.83712700	3.16971200	-1.17435300
C	1.13442700	3.88302000	0.01964300
C	1.25485100	3.10791900	1.20637700

C	1.08733500	1.74739300	1.15102700
N	0.82081900	1.09703200	0.00188300
H	0.44682400	1.24121200	-2.02917200
H	0.71600500	3.67716500	-2.12191500
H	1.47228200	3.56661500	2.16163900
H	1.17114400	1.13383200	2.04279700
N	1.29003200	5.21751900	0.02708800
C	1.56678800	5.91349500	1.27672100
H	1.64128000	6.98087200	1.07540100
H	0.76309900	5.75122000	2.00287000
H	2.51189800	5.57434000	1.71439500
C	1.15403000	5.97453800	-1.21021700
H	1.87962100	5.63608400	-1.95742700
H	0.14571300	5.87202400	-1.62603500
H	1.33891700	7.02679900	-1.00138200

Zero-point correction= 0.367641 (Hartree/Particle)

Thermal correction to Energy= 0.394007

Thermal correction to Enthalpy= 0.394951

Thermal correction to Gibbs Free Energy= 0.306406

Sum of electronic and zero-point Energies= -1232.493112

Sum of electronic and thermal Energies= -1232.466746

Sum of electronic and thermal Enthalpies= -1232.465802

Sum of electronic and thermal Free Energies= -1232.554348

$P(4\text{-}MeOC_6H_4)_3$

C	-0.12903800	-1.64391000	0.69130300
C	-0.94924700	-2.59971700	1.29583700
C	0.53108400	-1.99720300	-0.49532300
C	-1.13374700	-3.86605900	0.74183000

H	-1.46291800	-2.35767500	2.22391000
C	0.36737300	-3.25489300	-1.05503700
H	1.18139400	-1.28030700	-0.98977100
C	-0.46954600	-4.19672400	-0.44206200
H	-1.78088500	-4.57876100	1.24002700
H	0.87795000	-3.53337900	-1.97186100
C	1.49103800	0.71152100	0.69097900
C	1.46606300	1.46520000	-0.49219900
C	2.72953100	0.47365200	1.29198100
C	2.63703000	1.95231100	-1.05210400
H	0.51932200	1.67429100	-0.98341800
C	3.91831600	0.94674300	0.73758400
H	2.77723000	-0.09659800	2.21736100
C	3.87182500	1.69277600	-0.44293800
H	2.62226000	2.53762200	-1.96645700
H	4.85936600	0.73839200	1.23332200
C	-1.35882000	0.93672700	0.69076800
C	-1.76261800	2.13605400	1.28310700
C	-2.01058200	0.52890200	-0.48267800
C	-2.76942700	2.92632200	0.72988000
H	-1.28304400	2.47076200	2.20067500
C	-3.02059500	1.29698900	-1.04130800
H	-1.72575400	-0.40107100	-0.96751700
C	-3.40491000	2.50272000	-0.44030900
H	-3.05180000	3.85200700	1.21785300
H	-3.52922700	0.98380600	-1.94799300
O	-0.56661500	-5.39543300	-1.06520300
O	-4.39924600	3.18246000	-1.06025900
O	4.95875000	2.20751700	-1.06616100

C	-4.81709300	4.40755800	-0.48165200
H	-3.99545100	5.13193200	-0.44872100
H	-5.61033700	4.78906200	-1.12500400
H	-5.21056200	4.25528800	0.52968700
C	6.22756600	1.95950500	-0.48401100
H	6.44097700	0.88550100	-0.44029700
H	6.95641400	2.44747100	-1.13156900
H	6.29262100	2.38607000	0.52328600
C	-1.40978100	-6.37342900	-0.47964200
H	-2.44772200	-6.02519500	-0.43161100
H	-1.35104200	-7.24821300	-1.12777300
H	-1.06768100	-6.64189600	0.52623500
P	0.00128300	0.00161800	1.50457200

Zero-point correction= 0.377445 (Hartree/Particle)

Thermal correction to Energy= 0.400455

Thermal correction to Enthalpy= 0.401399

Thermal correction to Gibbs Free Energy= 0.323199

Sum of electronic and zero-point Energies= -1379.042601

Sum of electronic and thermal Energies= -1379.019591

Sum of electronic and thermal Enthalpies= -1379.018647

Sum of electronic and thermal Free Energies= -1379.096847

TS2

C	-3.61467600	-2.05132800	-2.13541700
C	-4.99177400	-1.89540800	-2.29050100
C	-5.56259600	-0.66263400	-1.99646000
C	-4.76282900	0.39490100	-1.55313000
C	-3.38400500	0.23944100	-1.40332500
C	-2.83033600	-0.99434900	-1.70407500

H	-5.59009100	-2.73446800	-2.63163000
H	-6.63255500	-0.52026500	-2.10720700
H	-5.21724400	1.35242600	-1.31459900
H	-2.77213200	1.05646800	-1.03926900
I	-0.78538500	-1.55224500	-1.51857600
C	-2.91578600	-3.33087900	-2.39733800
O	-1.59006600	-3.29480300	-2.16196800
O	-3.43598300	-4.35060300	-2.78284300
C	0.17653400	-0.49852900	2.70115300
C	1.03961600	-1.58104700	2.51941000
C	-0.28118100	-0.22041300	3.99911600
C	1.44835200	-2.37666200	3.59100400
H	1.41470400	-1.80299100	1.52165800
C	0.10815100	-1.00538300	5.07130600
H	-0.95640500	0.61435600	4.16985000
C	0.97619100	-2.09013400	4.87345300
H	2.12046300	-3.20784900	3.41220300
H	-0.24368200	-0.80038600	6.07769200
C	0.90390600	1.92248500	1.33607200
C	2.02301700	1.88649500	0.50201900
C	0.74354100	3.02248300	2.19369100
C	2.95300100	2.92697000	0.48461600
H	2.16847100	1.03232000	-0.15722400
C	1.64663100	4.07249200	2.17396900
H	-0.10426600	3.06552600	2.87291200
C	2.75127100	4.03536800	1.30907600
H	3.80587900	2.87038600	-0.18342000
H	1.52333000	4.93552100	2.82088200
O	1.29512200	-2.79792800	5.98261500

O	3.56125200	5.12184500	1.34162700
C	4.70518800	5.10778800	0.50640300
H	4.42668600	5.01816100	-0.55034700
H	5.21015100	6.06045200	0.66822500
H	5.38019700	4.28631400	0.77219300
C	2.16658900	-3.90508200	5.82672800
H	3.14250700	-3.59079500	5.43989500
H	2.29196200	-4.33217500	6.82201100
H	1.73493700	-4.65884100	5.15865500
P	-0.23714100	0.49597100	1.21423800
C	-0.56972100	2.51969000	-1.78216400
C	0.26476900	3.61456200	-1.90773700
C	1.54250500	3.44862900	-2.49151700
C	1.84724700	2.14664800	-2.95142600
C	0.93351200	1.12633700	-2.75621200
N	-0.26509000	1.27228000	-2.17255200
H	-1.54944300	2.66119700	-1.33228600
H	-0.06949700	4.57662100	-1.53906200
H	2.79367300	1.92005600	-3.42617100
H	1.19362100	0.11941500	-3.07680400
N	2.42809400	4.47394000	-2.58992400
C	2.11136000	5.76066300	-1.99501100
H	1.94942000	5.67401500	-0.91183600
H	2.94206100	6.44495400	-2.16782900
H	1.21171000	6.19595300	-2.44513700
C	3.73728600	4.24028400	-3.17248500
H	3.65168500	3.88428000	-4.20558400
H	4.29479200	5.17677900	-3.17969900
H	4.30982100	3.49765000	-2.60058300

C	-1.89907200	1.16035700	1.60666100
C	-2.89183100	0.25831200	2.00785000
C	-2.30040700	2.46456900	1.27551100
C	-4.23473200	0.62258900	2.07792900
H	-2.62120200	-0.76438700	2.26180400
C	-3.63520300	2.84130500	1.33073400
H	-1.56499200	3.19656700	0.95424800
C	-4.61419600	1.91977600	1.72129200
H	-4.96927700	-0.11106300	2.38912800
H	-3.94348700	3.84910400	1.06977100
O	-5.89181800	2.37068400	1.71721300
C	-6.91142300	1.44664400	2.05715600
H	-6.79591600	1.08603800	3.08549000
H	-7.85248600	1.99001100	1.96874900
H	-6.91694900	0.59276700	1.36954900
O	0.96815300	-3.16181300	-1.62635900
C	2.00144900	-2.54280000	-1.19836300
O	2.00507900	-1.34588900	-0.85067700
C	3.27401100	-3.34761500	-1.11290600
C	4.42749900	-2.75168100	-0.59978100
C	3.30825100	-4.67711900	-1.53804500
C	5.60999400	-3.48089600	-0.51125900
H	4.38007700	-1.71748600	-0.27399700
C	4.49183400	-5.40561800	-1.45264400
H	2.40345800	-5.12690900	-1.93355100
C	5.64286900	-4.80837700	-0.93876600
H	6.50570300	-3.01648900	-0.10985200
H	4.51770400	-6.43881000	-1.78555300
H	6.56536000	-5.37750200	-0.87136600

Zero-point correction= 0.744603 (Hartree/Particle)

Thermal correction to Energy= 0.795293

Thermal correction to Enthalpy= 0.796237

Thermal correction to Gibbs Free Energy= 0.652398

Sum of electronic and zero-point Energies= -2611.515853

Sum of electronic and thermal Energies= -2611.465164

Sum of electronic and thermal Enthalpies= -2611.464220

Sum of electronic and thermal Free Energies= -2611.608059

IM2

C	2.95602200	3.32915400	-0.61612000
C	4.29201000	3.59254100	-0.91546000
C	5.12598700	2.56877500	-1.34939100
C	4.61404300	1.28280500	-1.51471700
C	3.28026900	1.00390700	-1.21495500
C	2.48113700	2.03067300	-0.73354000
H	4.64542700	4.61398900	-0.81564300
H	6.16567600	2.77418300	-1.58260100
H	5.24769400	0.48322900	-1.88581200
H	2.89762400	0.00362900	-1.36900300
I	0.40441600	1.84598100	-0.21098400
C	2.00891100	4.44981600	-0.26448100
O	2.42229100	5.58862200	-0.09567500
O	0.76891700	4.08411600	-0.20603100
O	-2.36022900	2.47782700	0.60294900
C	-2.79523000	1.47748300	-0.01303400
O	-2.10300500	0.49074900	-0.39080100
C	-4.27968700	1.43308500	-0.33272500
C	-4.82021200	0.35460000	-1.03562400

C	-5.11427300	2.47652800	0.07214000
C	-6.18117300	0.31792700	-1.32959900
H	-4.15730800	-0.44681900	-1.34733900
C	-6.47581300	2.44222400	-0.21898000
H	-4.67701100	3.30916200	0.61381000
C	-7.01111700	1.36209600	-0.92088200
H	-6.59668500	-0.52260000	-1.87803000
H	-7.12024100	3.25681900	0.09869300
H	-8.07254200	1.33456900	-1.14975000
P	0.33800100	-0.77190800	0.13780700
C	-0.36086400	-1.63886600	-1.26428900
C	-1.53006000	-2.38785700	-1.13523000
C	0.26176700	-1.53360600	-2.51704900
C	-2.06581700	-3.05668000	-2.23153200
H	-2.03964400	-2.44408200	-0.17841400
C	-0.26389400	-2.19367900	-3.61192000
H	1.15655100	-0.92696300	-2.63777800
C	-1.43057600	-2.96276700	-3.47524400
H	-2.97365500	-3.63481300	-2.10775800
H	0.20278000	-2.12348400	-4.58870600
C	2.00647800	-1.38912000	0.43526000
C	2.75118700	-0.78014700	1.46132400
C	2.58888200	-2.40914100	-0.31731000
C	4.04928700	-1.17541700	1.71229400
H	2.30893500	0.01543800	2.05736600
C	3.90017900	-2.81406200	-0.07275800
H	2.02856400	-2.90170800	-1.10582900
C	4.63687500	-2.19247300	0.94026300
H	4.63776100	-0.71119100	2.49663600

H	4.32908700	-3.61034500	-0.66920900
C	-0.53474800	-1.11499900	1.68159700
C	-1.30610000	-0.17393400	2.36485400
C	-0.37567200	-2.40201100	2.22672600
C	-1.91897200	-0.50365300	3.57058800
H	-1.44719400	0.82765200	1.96973800
C	-0.99830500	-2.74130700	3.41312400
H	0.23562300	-3.14352400	1.71881600
C	-1.77406100	-1.79171300	4.09567100
H	-2.50714700	0.24760700	4.08363300
H	-0.89212000	-3.73378200	3.83819100
O	-1.86595700	-3.56758600	-4.59706700
O	-2.33338200	-2.21022900	5.24712500
O	5.90925300	-2.50098300	1.25272200
C	-3.05827700	-4.33574700	-4.51896000
H	-2.94451300	-5.17038400	-3.81873000
H	-3.90763600	-3.71400000	-4.21646000
H	-3.22972000	-4.72365600	-5.52281700
C	-3.12307700	-1.28226500	5.97777100
H	-3.47492700	-1.81866400	6.85876600
H	-2.52754600	-0.41754100	6.28947400
H	-3.98166700	-0.94483700	5.38763200
C	6.54840100	-3.52392200	0.50262300
H	6.02521700	-4.47954400	0.61477900
H	6.60539200	-3.25674200	-0.55802300
H	7.55503100	-3.60957700	0.91101500

Zero-point correction= 0.579478 (Hartree/Particle)

Thermal correction to Energy= 0.621044

Thermal correction to Enthalpy= 0.621988

Thermal correction to Gibbs Free Energy= 0.498837

Sum of electronic and zero-point Energies= -2229.588917

Sum of electronic and thermal Energies= -2229.547351

Sum of electronic and thermal Enthalpies= -2229.546407

Sum of electronic and thermal Free Energies= -2229.669558

Boc-Gly-OH

C	-1.73606300	0.48308500	-0.31969600
H	-1.29439700	1.24505200	0.32611400
C	-3.20908600	0.41627900	0.01433600
N	-1.05657100	-0.77774900	-0.13190500
H	-1.57151200	-1.63414800	-0.28962900
O	-3.92353500	1.38808700	0.02616900
O	-3.64299300	-0.82440400	0.26568100
C	0.29647700	-0.91945200	-0.08752700
O	0.85487000	-2.00198200	-0.06203800
O	0.89825100	0.28083700	-0.05868400
C	2.34966500	0.38270800	0.05314000
C	3.01556800	-0.24601300	-1.16588800
H	4.08889700	-0.03269300	-1.13977500
H	2.87128200	-1.32708800	-1.18275800
H	2.60279300	0.18502600	-2.08398900
C	2.58057200	1.88856900	0.06934500
H	2.20127900	2.34437700	-0.85047900
H	2.07044600	2.34565200	0.92293800
H	3.65088200	2.09961300	0.14808200
C	2.82177100	-0.24385500	1.36047400
H	2.27198400	0.18675800	2.20389900
H	2.67921500	-1.32538100	1.35632800

H	3.88591600	-0.02832000	1.49926700
H	-1.66489600	0.85299400	-1.35152800
H	-4.60206400	-0.77582900	0.45072000

Zero-point correction= 0.209500 (Hartree/Particle)

Thermal correction to Energy= 0.223060

Thermal correction to Enthalpy= 0.224004

Thermal correction to Gibbs Free Energy= 0.168229

Sum of electronic and zero-point Energies= -629.802546

Sum of electronic and thermal Energies= -629.788985

Sum of electronic and thermal Enthalpies= -629.788041

Sum of electronic and thermal Free Energies= -629.843816

HOBz

C	2.56192900	0.04215800	-0.00012400
C	1.83660800	1.23292200	0.00008600
C	0.44538100	1.20171600	-0.00002900
C	-0.21788400	-0.02816900	0.00008800
C	0.50901000	-1.22102900	0.00019100
C	1.89859300	-1.18507200	-0.00010500
H	3.64753600	0.07038300	-0.00015100
H	2.35530700	2.18640700	0.00021900
H	-0.12513100	2.12426200	0.00006100
H	-0.02707800	-2.16472400	0.00019100
H	2.46529200	-2.11082400	-0.00022300
C	-1.70299000	-0.11787200	0.00008600
O	-2.32743500	-1.15535900	-0.00005500
O	-2.30179700	1.08294600	-0.00005800
H	-3.26595100	0.92587600	-0.00034900

Zero-point correction= 0.116970 (Hartree/Particle)

Thermal correction to Energy= 0.124045

Thermal correction to Enthalpy= 0.124989

Thermal correction to Gibbs Free Energy= 0.084935

Sum of electronic and zero-point Energies= -420.543158

Sum of electronic and thermal Energies= -420.536084

Sum of electronic and thermal Enthalpies= -420.535139

Sum of electronic and thermal Free Energies= -420.575194

IM3

C	-3.47108300	-3.67142100	-0.93109100
C	-3.20336300	-4.99918200	-0.59596400
C	-2.15223500	-5.31135200	0.25832400
C	-1.36139300	-4.29475700	0.79136600
C	-1.61058400	-2.95926500	0.47269400
C	-2.66035700	-2.69037100	-0.38873800
H	-3.84147600	-5.76711800	-1.02130400
H	-1.94828700	-6.34598900	0.51348400
H	-0.54136200	-4.52988000	1.46197000
H	-0.98869600	-2.18037300	0.89577300
I	-3.19256300	-0.70293200	-1.01411400
C	-4.62578400	-3.31841900	-1.84635800
O	-4.77067600	-2.05452100	-2.04134700
O	-5.32152900	-4.20908100	-2.31732100
C	3.81450700	1.74951900	0.31906600
H	3.63570500	0.94376300	1.03352100
C	2.45489300	2.26286800	-0.21737000
N	4.62236200	1.29529500	-0.79111600
H	4.25443600	1.56185000	-1.69885600
O	1.58491700	2.50071200	0.65249100

O	2.36764200	2.40619000	-1.45978400
C	5.65985900	0.43590900	-0.75996800
O	6.28708900	0.08103400	-1.74993100
O	5.91984400	0.03992000	0.50476500
C	7.02688900	-0.85605700	0.78288200
C	8.34919500	-0.18312100	0.42687900
H	9.18145700	-0.81568800	0.75245100
H	8.42785000	-0.02188500	-0.64937200
H	8.43033300	0.78143100	0.93914800
C	6.92602600	-1.06122900	2.29042000
H	7.00538700	-0.10291800	2.81309600
H	5.96687500	-1.52098700	2.55032300
H	7.73179800	-1.71574500	2.63551800
C	6.84674900	-2.18652200	0.05710000
H	5.84626500	-2.58717100	0.24778700
H	6.97569200	-2.06761000	-1.01913900
H	7.58602500	-2.90330600	0.43012100
H	4.30507200	2.56831200	0.86340900
C	0.30092800	-0.15288500	-0.42794100
C	0.58428200	-0.25043800	-1.79335200
C	1.21804400	-0.65485100	0.51103200
C	1.77875900	-0.80896100	-2.22935900
H	-0.12776000	0.11522300	-2.52898100
C	2.40046900	-1.22120200	0.08294000
H	1.01277200	-0.58946600	1.57518200
C	2.70041300	-1.28383700	-1.28777100
H	1.98673800	-0.85870000	-3.29104500
H	3.13161900	-1.59658800	0.79193100
C	-1.39666300	0.56247600	1.90831500

C	-0.51219200	1.37470800	2.64798400
C	-2.37134300	-0.18631000	2.57008200
C	-0.62096300	1.42064500	4.02434100
H	0.26590400	1.94087100	2.13548100
C	-2.48349100	-0.13682900	3.95597400
H	-3.05853900	-0.82016100	2.01651100
C	-1.60790000	0.67232100	4.68779600
H	0.04957900	2.03477400	4.61654900
H	-3.24938400	-0.72445200	4.44708000
C	-1.57343200	2.20118500	-0.53494200
C	-2.77944800	2.81020100	-0.16273800
C	-0.68368800	2.86374700	-1.39537100
C	-3.12023300	4.06309300	-0.65442800
H	-3.46029000	2.31019600	0.52349900
C	-1.02425000	4.11174200	-1.88388200
H	0.29600100	2.44965300	-1.62678400
C	-2.23965500	4.71603900	-1.52800500
H	-4.05559600	4.51872000	-0.35338700
H	-0.34824900	4.65359600	-2.53742900
O	3.88964300	-1.82594500	-1.59516000
O	-2.47271000	5.92646500	-2.06550100
O	-1.63422100	0.79572800	6.02617200
C	-3.68148600	6.58922700	-1.72172500
H	-3.73133900	6.78249400	-0.64502100
H	-3.66621500	7.53578300	-2.26142200
H	-4.55362800	6.00464300	-2.03343600
C	-2.62086800	0.06711500	6.74422000
H	-2.49390200	-1.01147800	6.60293400
H	-2.47056000	0.31713500	7.79407400

H	-3.62927700	0.36436700	6.43735700
C	4.28862800	-1.83103500	-2.95883300
H	4.32176700	-0.81304900	-3.35764900
H	5.29166500	-2.25425300	-2.97499100
H	3.61247700	-2.45149900	-3.55749400
P	-1.22646000	0.57622300	0.12322900

Zero-point correction= 0.672176 (Hartree/Particle)

Thermal correction to Energy= 0.719763

Thermal correction to Enthalpy= 0.720707

Thermal correction to Gibbs Free Energy= 0.585488

Sum of electronic and zero-point Energies= -2438.852253

Sum of electronic and thermal Energies= -2438.804667

Sum of electronic and thermal Enthalpies= -2438.803722

Sum of electronic and thermal Free Energies= -2438.938942

TS3

C	-3.19971400	-3.81249300	-0.76041500
C	-2.78767800	-5.14166800	-0.64018900
C	-1.53204400	-5.46173200	-0.13742900
C	-0.66618400	-4.44587600	0.26118300
C	-1.05183700	-3.10964500	0.15493100
C	-2.30934200	-2.83019300	-0.35626400
H	-3.48889400	-5.90784100	-0.95495800
H	-1.22740500	-6.49995400	-0.05373500
H	0.31705600	-4.67989300	0.65676500
H	-0.37386400	-2.32490600	0.46498400
I	-3.00026500	-0.80184800	-0.59326200
C	-4.58789900	-3.49473900	-1.31172300
O	-4.85502200	-2.25370900	-1.37576800

O	-5.31111300	-4.43807400	-1.63595500
C	3.43121500	2.06606400	0.24953300
H	3.39174100	1.27064200	0.99567300
C	2.00814900	2.32504400	-0.29421800
N	4.28975200	1.69814700	-0.85803700
H	3.87643300	1.88693800	-1.76590000
O	1.08513400	2.34477600	0.57045500
O	1.90403000	2.47288700	-1.52714400
C	5.31892600	0.82163600	-0.84468700
O	5.89899300	0.44330800	-1.85326500
O	5.62837700	0.45139300	0.41516300
C	6.79026200	-0.38219600	0.67503100
C	8.05995200	0.32918700	0.21647100
H	8.93549700	-0.23437800	0.55449400
H	8.09275100	0.41379500	-0.87069300
H	8.10746100	1.33196400	0.65414500
C	6.77835400	-0.51603600	2.19350300
H	6.86344500	0.46741200	2.66618200
H	5.84699600	-0.98410900	2.52847900
H	7.61780000	-1.13599800	2.52171200
C	6.63382000	-1.75156600	0.02173900
H	5.70115400	-2.22385900	0.34609600
H	6.62242100	-1.66852600	-1.06525500
H	7.46833700	-2.39172300	0.32731600
H	3.78918300	2.97449500	0.75289700
C	0.44625200	-0.14046100	-0.52311100
C	0.58326300	-0.33212200	-1.89716600
C	1.45017100	-0.58052500	0.35617900
C	1.73471700	-0.92289600	-2.40885400

H	-0.19907300	-0.01086300	-2.57997100
C	2.58083200	-1.18779100	-0.14473300
H	1.35581500	-0.41347900	1.42505400
C	2.74411100	-1.34018400	-1.53272200
H	1.84056300	-1.04367700	-3.48013600
H	3.38235600	-1.51102800	0.51169100
C	-1.06004300	0.73384700	1.95107400
C	-0.84517600	1.94451900	2.63452000
C	-1.36278900	-0.42471600	2.67213000
C	-0.92953000	1.97687300	4.01223500
H	-0.59055200	2.84032400	2.08037100
C	-1.43430700	-0.39966700	4.05910600
H	-1.56876300	-1.35768500	2.15467800
C	-1.21808000	0.80789700	4.73530000
H	-0.76878600	2.89952500	4.55966100
H	-1.67401900	-1.30979500	4.59492800
C	-1.66295600	2.15403200	-0.59186600
C	-2.87590600	2.62332700	-0.07253500
C	-1.08880200	2.77384100	-1.71258400
C	-3.52140500	3.70720400	-0.65709900
H	-3.32597300	2.15271700	0.79883400
C	-1.73397000	3.84578000	-2.29686000
H	-0.10839700	2.46295100	-2.06184700
C	-2.95172600	4.31897000	-1.77986600
H	-4.45395600	4.06008400	-0.23424300
H	-1.30486500	4.35360900	-3.15441100
O	3.90133000	-1.89986300	-1.91657100
O	-3.49146400	5.36816900	-2.42338700
O	-1.27467800	0.94560800	6.07000400

C	-4.71672100	5.89489000	-1.93238300
H	-4.60598700	6.25356300	-0.90373100
H	-4.96456000	6.73111800	-2.58555100
H	-5.51351800	5.14510700	-1.97926500
C	-1.56686000	-0.20668800	6.85007500
H	-0.80343100	-0.97913900	6.71042300
H	-1.56237200	0.12734200	7.88713100
H	-2.55309300	-0.61086100	6.59897600
C	4.24335500	-1.84707100	-3.29431800
H	4.22959000	-0.81433500	-3.65468800
H	5.25619200	-2.24234500	-3.36606600
H	3.56564500	-2.46716900	-3.89117000
P	-0.95162000	0.70705700	0.16987500

Zero-point correction= 0.672209 (Hartree/Particle)

Thermal correction to Energy= 0.718953

Thermal correction to Enthalpy= 0.719897

Thermal correction to Gibbs Free Energy= 0.588375

Sum of electronic and zero-point Energies= -2438.849846

Sum of electronic and thermal Energies= -2438.803102

Sum of electronic and thermal Enthalpies= -2438.802158

Sum of electronic and thermal Free Energies= -2438.933681

IM4

C	2.80004700	-0.42576600	-0.20795500
H	2.98704200	0.60492300	-0.52358400
C	1.44318500	-0.48117100	0.43569700
N	3.79258300	-0.86161600	0.73782300
H	3.51442000	-1.50686700	1.46710000
O	0.49115600	-0.00011200	-0.42812200

O	1.18037200	-0.88563800	1.53195000
C	5.13620300	-0.72782300	0.54814600
O	5.96607200	-1.21381500	1.29317900
O	5.38286600	0.01293700	-0.54297200
C	6.75417600	0.34353900	-0.92453700
C	7.52504600	-0.92852000	-1.25781800
H	8.49796800	-0.65827200	-1.68014100
H	7.68518000	-1.53999100	-0.36882200
H	6.97887300	-1.51535200	-2.00370200
C	6.55889300	1.19337400	-2.17361500
H	6.03870300	0.62225700	-2.94881800
H	5.97088600	2.08675000	-1.94142900
H	7.53091400	1.50730400	-2.56432700
C	7.42374800	1.15789000	0.17653200
H	6.80469900	2.02362300	0.43407200
H	7.58815300	0.55682400	1.07190700
H	8.39003100	1.52321300	-0.18520700
H	2.76123000	-1.04150500	-1.11757700
C	-1.34795600	1.05516600	1.35939400
C	-2.42113600	0.84620100	2.23048000
C	-0.50165500	2.16391700	1.54095100
C	-2.65477600	1.72396900	3.28240900
H	-3.08586900	-0.00197700	2.09448400
C	-0.72543700	3.03343000	2.58771600
H	0.33069000	2.34267700	0.86532900
C	-1.80482700	2.82170000	3.46454000
H	-3.48787700	1.54288000	3.95031300
H	-0.08048600	3.88975400	2.75328500
C	-1.77958000	0.71516500	-1.53830000

C	-1.26321300	0.35567400	-2.79684900
C	-2.84646400	1.61293900	-1.45251100
C	-1.81404400	0.89248200	-3.94220400
H	-0.43067700	-0.33751600	-2.87538600
C	-3.40615400	2.15426900	-2.60378900
H	-3.24575700	1.90228800	-0.48409900
C	-2.88937600	1.79449100	-3.85427400
H	-1.42887500	0.63665900	-4.92349000
H	-4.22942500	2.85278500	-2.51732100
C	-1.71292400	-1.64634500	0.23452400
C	-2.38495700	-2.32237900	-0.79193100
C	-1.53173600	-2.27973600	1.48176600
C	-2.86111100	-3.61196900	-0.59866000
H	-2.55059400	-1.84342100	-1.75211300
C	-2.00790500	-3.55773200	1.67939100
H	-1.01838000	-1.76875800	2.28934900
C	-2.67299600	-4.23576200	0.64130300
H	-3.37913500	-4.11327900	-1.40686500
H	-1.88019600	-4.06230400	2.63115600
O	-1.94122900	3.72647900	4.44523000
O	-3.09706800	-5.47186800	0.93451300
O	-3.35169400	2.26133400	-5.02417600
C	-3.77616900	-6.20881100	-0.07758700
H	-3.13136800	-6.35972600	-0.94902400
H	-4.01874900	-7.17178300	0.37008100
H	-4.69740100	-5.69988600	-0.37857200
C	-4.43108500	3.18864300	-4.99552200
H	-4.15131500	4.09705700	-4.45215600
H	-4.63960500	3.43354900	-6.03639700

H	-5.31907000	2.74073600	-4.53787100
C	-3.02196600	3.57021100	5.35835500
H	-2.93663500	2.62711600	5.90769100
H	-2.94601700	4.40598600	6.05309200
H	-3.98352600	3.61195700	4.83672900
P	-1.11631300	0.00178700	-0.05021100

Zero-point correction= 0.578627 (Hartree/Particle)

Thermal correction to Energy= 0.616110

Thermal correction to Enthalpy= 0.617054

Thermal correction to Gibbs Free Energy= 0.503297

Sum of electronic and zero-point Energies= -2008.129103

Sum of electronic and thermal Energies= -2008.091621

Sum of electronic and thermal Enthalpies= -2008.090676

Sum of electronic and thermal Free Energies= -2008.204433

H-Gly-OMe

C	1.43144100	0.45440500	0.19753500
H	1.61080100	0.67130200	1.26375800
H	1.85183900	1.28606000	-0.37245500
N	1.99993000	-0.80402700	-0.27157100
C	-0.06886100	0.51286600	0.01458500
O	-0.69821500	1.53468800	-0.13531300
O	-0.63459700	-0.69546400	0.10075000
C	-2.06102000	-0.71547200	-0.01219400
H	-2.35110600	-1.76196400	0.07126900
H	-2.51455700	-0.12922100	0.79019300
H	-2.37144400	-0.31079300	-0.97794800
H	1.62622100	-1.56399600	0.29373900
H	3.00187500	-0.78779000	-0.09061800

Zero-point correction= 0.109733 (Hartree/Particle)

Thermal correction to Energy= 0.116769

Thermal correction to Enthalpy= 0.117713

Thermal correction to Gibbs Free Energy= 0.078455

Sum of electronic and zero-point Energies= -323.483528

Sum of electronic and thermal Energies= -323.476493

Sum of electronic and thermal Enthalpies= -323.475549

Sum of electronic and thermal Free Energies= -323.514807

IM5

C	-0.89989600	-1.27065500	-2.98387800
H	-0.47741200	-2.13278100	-3.51114800
C	0.17423400	-0.74733800	-2.02914100
N	-2.10594600	-1.68118200	-2.30035100
H	-2.02657600	-2.46497300	-1.66298500
O	-0.35422200	0.34146700	-1.24385500
O	0.45662200	-1.61562200	-1.01234200
C	-3.17091600	-0.87083000	-2.03683700
O	-4.07682800	-1.18507800	-1.28230300
O	-3.10888600	0.26689800	-2.74563300
C	-4.12685100	1.29500200	-2.58394800
C	-4.20567600	1.75006400	-1.12946000
H	-4.79346300	2.67213700	-1.07293800
H	-4.67427200	0.99476700	-0.49751900
H	-3.20027400	1.96048400	-0.74768000
C	-3.60157600	2.42455100	-3.46181400
H	-2.62833200	2.76914200	-3.09732200
H	-3.48707400	2.08404000	-4.49567700
H	-4.29984000	3.26631600	-3.44652400

C	-5.46716000	0.78836300	-3.10470800
H	-5.36478700	0.44438100	-4.13919800
H	-5.83983300	-0.03304400	-2.49064400
H	-6.19720900	1.60396400	-3.08438600
C	-0.40642000	1.30185700	1.10168700
C	-0.65386800	2.50689700	0.44149700
C	-0.50619600	1.30025300	2.50386300
C	-1.00568800	3.66965500	1.13272100
H	-0.57221300	2.55273000	-0.63918200
C	-0.84461800	2.44292100	3.20863400
H	-0.31142700	0.38591800	3.06003000
C	-1.10276700	3.63889000	2.52417300
H	-1.19676500	4.58134500	0.57817400
H	-0.92152400	2.43593800	4.29153600
C	1.93920900	-0.18447000	0.63590900
C	2.76574200	-1.30675600	0.43027800
C	2.53996200	0.99155100	1.09147300
C	4.12371500	-1.25177900	0.68076400
H	2.33706400	-2.22612700	0.05034200
C	3.91321700	1.07124400	1.32094100
H	1.94599200	1.88321700	1.25798300
C	4.71071200	-0.05493800	1.11540900
H	4.76020000	-2.11590000	0.51711200
H	4.34179800	2.00934700	1.65346100
C	-0.82579800	-1.42367200	1.20185500
C	-0.24950300	-2.52902700	1.82463500
C	-2.21230100	-1.24464400	1.28429900
C	-1.02842500	-3.43904000	2.53834600
H	0.82340400	-2.69175800	1.77252900

C	-3.00184200	-2.15919300	1.96226000
H	-2.68510900	-0.39175000	0.80371900
C	-2.41306100	-3.25702800	2.60268100
H	-0.55026700	-4.27922200	3.02817000
H	-4.07928600	-2.03884100	2.01365900
O	-1.43505900	4.70242200	3.29455000
O	-3.26179700	-4.08467400	3.25694300
O	6.05149100	-0.08443000	1.28456100
C	-2.70657300	-5.21288700	3.91215700
H	-1.99974600	-4.91052800	4.69292200
H	-3.54578000	-5.73893600	4.36780300
H	-2.20344800	-5.87729300	3.20065900
C	6.69613400	1.12735600	1.64609200
H	6.34950000	1.48425100	2.62187900
H	7.76003100	0.89651400	1.70088000
H	6.52846000	1.90366500	0.89088900
C	-1.70974100	5.92760500	2.63617000
H	-2.55900500	5.82804500	1.95075100
H	-1.95917500	6.64201000	3.42107000
H	-0.83445400	6.28589600	2.08261200
P	0.15780500	-0.24321900	0.22143100
H	-1.14750100	-0.50569500	-3.72106300
C	2.37915000	-1.27568300	-2.98811100
H	2.19479100	-2.16222000	-2.37518000
H	2.38351800	-1.60985100	-4.03437000
N	1.36961500	-0.28038800	-2.70157700
C	3.81020200	-0.85662100	-2.68481900
O	4.74522500	-1.60795700	-2.86012100
O	3.94427300	0.39539200	-2.25098500

C	5.29175100	0.81245900	-2.02068300
H	5.22395600	1.82427800	-1.62115800
H	5.78881000	0.15039700	-1.30797900
H	5.85267200	0.81446800	-2.95929400
H	1.13433900	0.27294000	-3.52108000

Zero-point correction= 0.679912 (Hartree/Particle)

Thermal correction to Energy= 0.723224

Thermal correction to Enthalpy= 0.724168

Thermal correction to Gibbs Free Energy= 0.600853

Sum of electronic and zero-point Energies= -2331.175356

Sum of electronic and thermal Energies= -2331.132044

Sum of electronic and thermal Enthalpies= -2331.131100

Sum of electronic and thermal Free Energies= -2331.254416

TS4

C	-0.29340800	-2.12572000	-2.53701000
H	0.06285800	-3.13528200	-2.76173800
C	0.90427700	-1.35118600	-1.95806000
N	-1.41946700	-2.20757800	-1.64252900
H	-1.33498000	-2.76003800	-0.79979700
O	0.31854900	0.07929300	-1.68743500
O	1.36500400	-1.71506000	-0.79031800
C	-2.48537700	-1.37486000	-1.73336000
O	-2.66636000	-0.56946300	-2.63391100
O	-3.32552800	-1.59754100	-0.70260800
C	-4.64736100	-0.98855100	-0.68506300
C	-4.53957900	0.52954100	-0.57993400
H	-5.53424700	0.95115200	-0.40015900
H	-3.89330000	0.80281800	0.26116800

H	-4.13341600	0.96002800	-1.49660000
C	-5.44335100	-1.42321400	-1.91239800
H	-5.01813700	-1.01011600	-2.82825400
H	-5.45560200	-2.51592900	-1.98501900
H	-6.47661500	-1.07483500	-1.81554700
C	-5.27439200	-1.56751300	0.57789900
H	-5.30355200	-2.66039600	0.52288700
H	-4.69803500	-1.27904400	1.46208300
H	-6.29714300	-1.19559900	0.68991700
C	-0.33182100	2.01926600	-0.13764400
C	-1.35971200	2.27395900	-1.04939700
C	-0.01818400	3.00142100	0.81442100
C	-2.05867000	3.47989900	-1.03044700
H	-1.62916300	1.51528200	-1.78053900
C	-0.70192500	4.20511300	0.84111500
H	0.76450400	2.82440000	1.54691800
C	-1.72760200	4.45260300	-0.08252700
H	-2.85028200	3.64748100	-1.75174000
H	-0.46184400	4.97222700	1.57040900
C	2.08805000	0.66250900	0.54437200
C	2.62312100	-0.14333700	1.55738200
C	2.83790900	1.73881300	0.06797300
C	3.87087700	0.12984800	2.08430300
H	2.05812100	-0.98962200	1.93527000
C	4.10678000	2.01109600	0.57387300
H	2.43601300	2.38449800	-0.70958200
C	4.62524400	1.20243900	1.58874200
H	4.29465300	-0.48279200	2.87355800
H	4.66981200	2.84852400	0.17928100

C	-0.58832300	-0.55626900	1.06395600
C	-0.38040600	-1.90555100	1.38451200
C	-1.60715100	0.12969800	1.75030400
C	-1.16566600	-2.55883900	2.32822400
H	0.39125500	-2.45089100	0.85435000
C	-2.38865300	-0.50854300	2.69877800
H	-1.80631600	1.17608000	1.55149000
C	-2.18358300	-1.86170500	2.98851800
H	-0.97883700	-3.60576700	2.53712700
H	-3.17604700	0.02532300	3.22182900
O	-2.33683400	5.65403300	0.02225500
O	-3.01113400	-2.40431400	3.90468500
O	5.83676700	1.38190100	2.15982900
C	-2.84228200	-3.77971200	4.21687600
H	-1.84993500	-3.96976900	4.63986800
H	-3.60482100	-4.01343300	4.95973300
H	-2.99126000	-4.40556000	3.33039800
C	6.64050900	2.44897700	1.68217300
H	6.14930900	3.41579500	1.83892800
H	7.56285200	2.41287400	2.26215400
H	6.87295700	2.32376400	0.61890700
C	-3.38455300	5.94455600	-0.89016000
H	-4.21122400	5.23368000	-0.78256700
H	-3.73189200	6.94658200	-0.63803900
H	-3.02319200	5.93273200	-1.92421200
P	0.44041000	0.36991000	-0.12458000
H	-0.63521200	-1.65971300	-3.46424600
C	3.15946700	-0.70639300	-2.58598800
N	1.88137000	-1.24163800	-3.00332200

C	3.92356000	-1.63526300	-1.66725100
O	4.67806100	-1.24748000	-0.80419600
O	3.77286000	-2.92650100	-1.98360100
C	4.43659600	-3.83463100	-1.10936900
H	4.20683200	-4.83286600	-1.48172400
H	5.51685800	-3.66871500	-1.11775300
H	4.06249100	-3.71786500	-0.08858700
H	1.51234900	-0.72965800	-3.80030600
H	3.77694300	-0.56624100	-3.48077500
H	3.10034800	0.25709200	-2.06591000

Zero-point correction= 0.679239 (Hartree/Particle)

Thermal correction to Energy= 0.722089

Thermal correction to Enthalpy= 0.723033

Thermal correction to Gibbs Free Energy= 0.602715

Sum of electronic and zero-point Energies= -2331.176131

Sum of electronic and thermal Energies= -2331.133281

Sum of electronic and thermal Enthalpies= -2331.132336

Sum of electronic and thermal Free Energies= -2331.252655

O=P(4-MeOC_6H_4)$_3$

C	0.43244300	-1.55440900	0.42344500
C	0.49076100	-2.66892100	1.25745000
C	0.72154100	-1.71159500	-0.94142900
C	0.82862400	-3.92738600	0.75993500
H	0.27206100	-2.54895900	2.31488400
C	1.05747800	-2.95317100	-1.44969900
H	0.69600500	-0.85474100	-1.61069700
C	1.11231100	-4.07014400	-0.60031100
H	0.86865000	-4.77588100	1.43273800

H	1.28750900	-3.08913900	-2.50165700
C	1.14534200	1.24194900	0.54387700
C	0.78161200	2.59113600	0.40120400
C	2.47579700	0.88155300	0.31938000
C	1.71754200	3.54109700	0.03266000
H	-0.24584700	2.89949900	0.57688100
C	3.42967900	1.82616800	-0.05206400
H	2.78431900	-0.15480600	0.42994700
C	3.04865800	3.16336700	-0.19845000
H	1.44563800	4.58516600	-0.08564200
H	4.45266100	1.51347100	-0.22489200
C	-1.63735600	0.49993200	0.41241700
C	-2.77396900	0.45909500	1.21791600
C	-1.77310500	0.86784000	-0.93534800
C	-4.03353100	0.77147300	0.70729200
H	-2.67130800	0.18448800	2.26405000
C	-3.01594100	1.17900200	-1.45673800
H	-0.89794300	0.92536000	-1.57827000
C	-4.15507700	1.13070100	-0.63731400
H	-4.89945000	0.73501600	1.35790100
H	-3.13591700	1.46898600	-2.49578700
O	1.45310000	-5.23764600	-1.18999400
O	-5.32230900	1.45275200	-1.23786200
O	3.88759700	4.15830100	-0.55901200
C	-6.50041100	1.42201900	-0.44728900
H	-6.43881900	2.13539900	0.38197400
H	-7.31561200	1.70721600	-1.11242000
H	-6.68607700	0.41686800	-0.05297400
C	5.25018100	3.82745900	-0.78138900

H	5.35481700	3.11444300	-1.60674200
H	5.74593200	4.76210100	-1.04386000
H	5.70858000	3.41217900	0.12260900
C	1.54157400	-6.39010500	-0.36686500
H	0.57606300	-6.62020400	0.09694600
H	1.83171800	-7.20820200	-1.02621100
H	2.30133800	-6.26173700	0.41222000
P	-0.05292200	0.03072600	1.15675100
O	-0.15656200	-0.06962900	2.65507500

Zero-point correction= 0.382162 (Hartree/Particle)

Thermal correction to Energy= 0.406153

Thermal correction to Enthalpy= 0.407097

Thermal correction to Gibbs Free Energy= 0.326734

Sum of electronic and zero-point Energies= -1454.271327

Sum of electronic and thermal Energies= -1454.247336

Sum of electronic and thermal Enthalpies= -1454.246392

Sum of electronic and thermal Free Energies= -1454.326755

II

C	0.02725600	-0.65879300	0.17700800
H	0.40673200	-1.62158300	0.53794900
C	-1.42858300	-0.81524600	-0.23721600
N	0.77019900	-0.18775300	-0.96346600
H	0.24158000	0.01118900	-1.80500500
O	-1.83371500	-0.49509300	-1.34836300
C	2.08592200	0.13328700	-0.95985500
O	2.67805400	0.58351400	-1.92619700
O	2.62613700	-0.12421400	0.24671200
C	4.03960800	0.12833000	0.49565800

C	4.34602500	1.61392900	0.33906300
H	5.37082700	1.80802400	0.67129000
H	4.24602100	1.93120300	-0.69969200
H	3.66633800	2.20581700	0.96108800
C	4.20764000	-0.29861700	1.94884000
H	3.56051400	0.29689800	2.60039700
H	3.94957400	-1.35529300	2.07011500
H	5.24576000	-0.15446200	2.26177600
C	4.89971900	-0.73981100	-0.41654100
H	4.60439000	-1.79019300	-0.32335100
H	4.80464400	-0.43405800	-1.45926800
H	5.94847700	-0.65078700	-0.11566300
H	0.07794700	0.04828000	1.01468200
C	-3.65831300	-1.41873000	0.44452800
H	-3.85959300	-2.06085100	-0.41632600
H	-4.15066700	-1.85357100	1.31790600
N	-2.24307000	-1.33520500	0.70539200
C	-4.31660000	-0.07835500	0.15346100
O	-5.33663600	0.02879200	-0.48461200
O	-3.67981300	0.94517400	0.72303700
C	-4.26136200	2.23104600	0.48269000
H	-3.62237000	2.94666100	0.99801300
H	-4.28197700	2.44168800	-0.58873400
H	-5.27723400	2.27021600	0.88200900
H	-1.90096600	-1.46609400	1.64889500

Zero-point correction= 0.294582 (Hartree/Particle)

Thermal correction to Energy= 0.314163

Thermal correction to Enthalpy= 0.315107

Thermal correction to Gibbs Free Energy= 0.243488

Sum of electronic and zero-point Energies= -876.939746

Sum of electronic and thermal Energies= -876.920164

Sum of electronic and thermal Enthalpies= -876.919220

Sum of electronic and thermal Free Energies= -876.990839

^{1}H NMR, ^{13}C NMR of Products

IBA-OBz

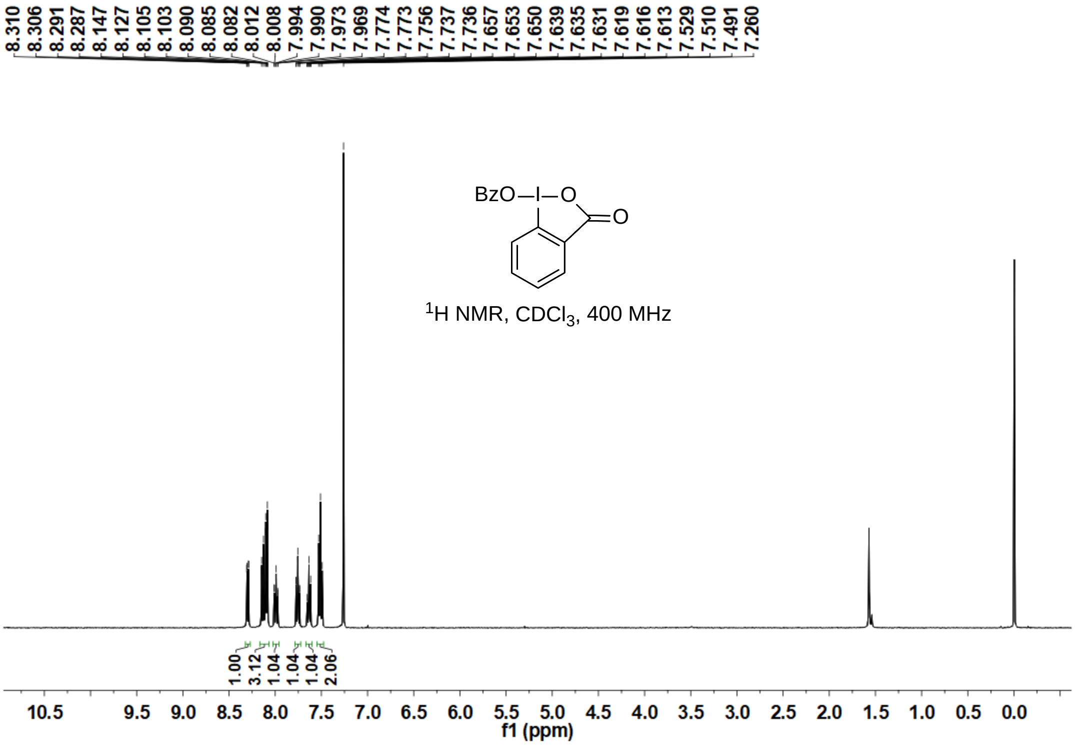

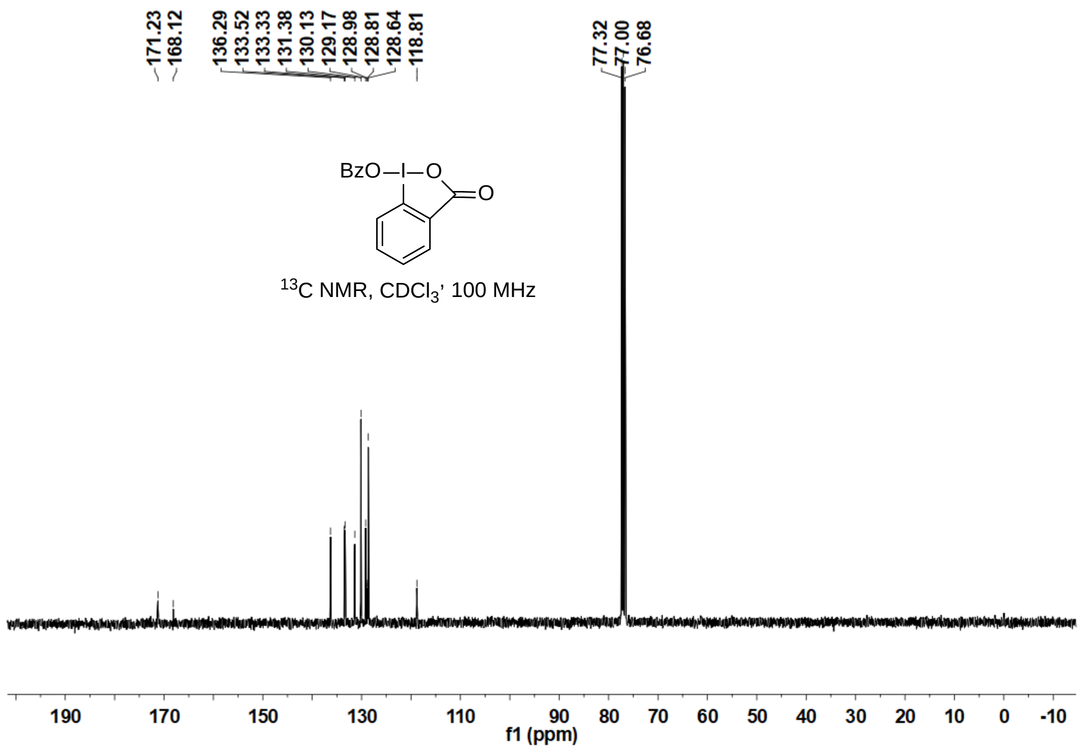

Boc-L-Phe-L-Leu-OMe (3-1)

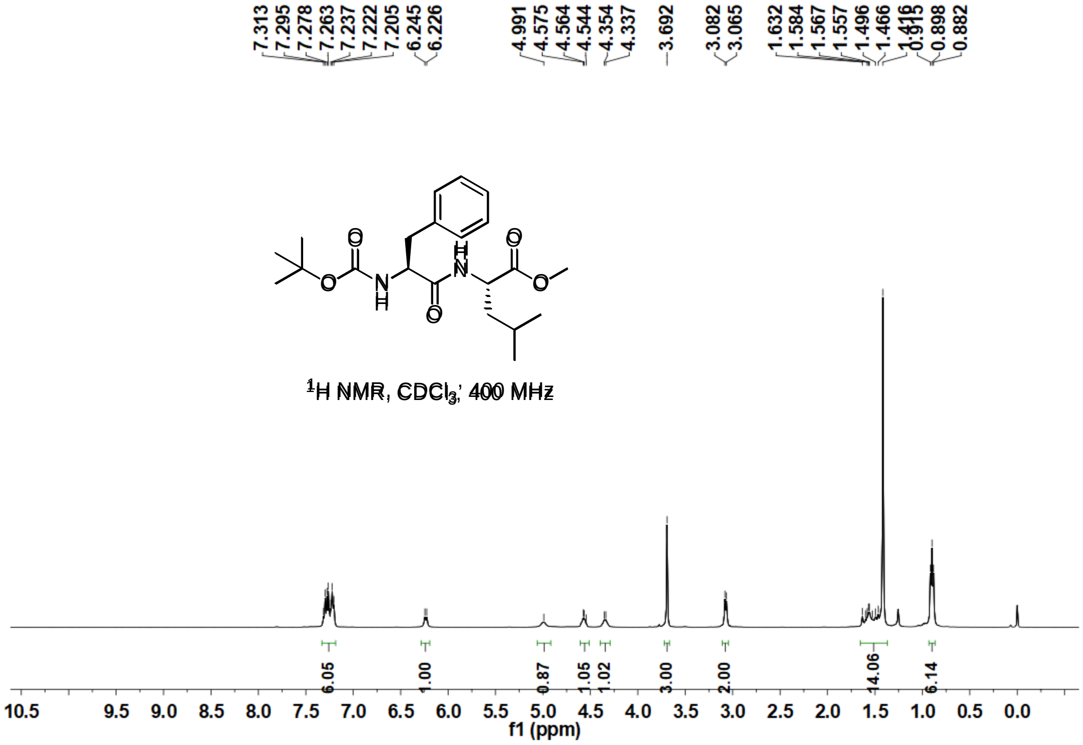

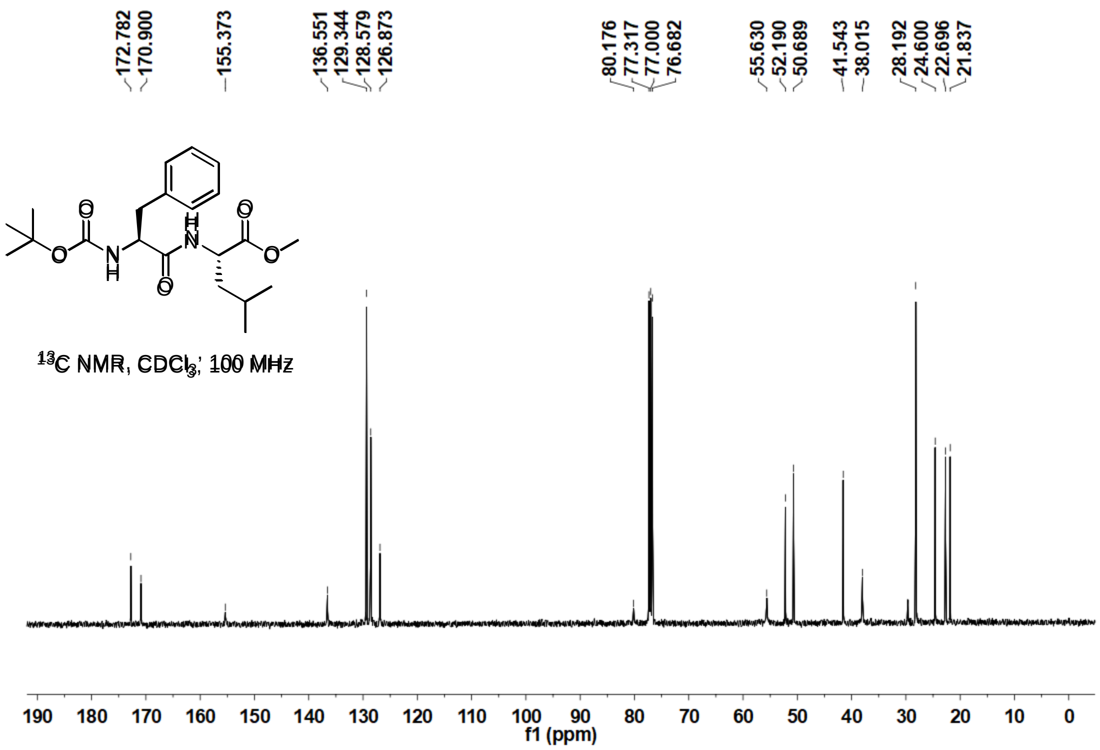

Boc-Gly-Gly-OMe (3-2)

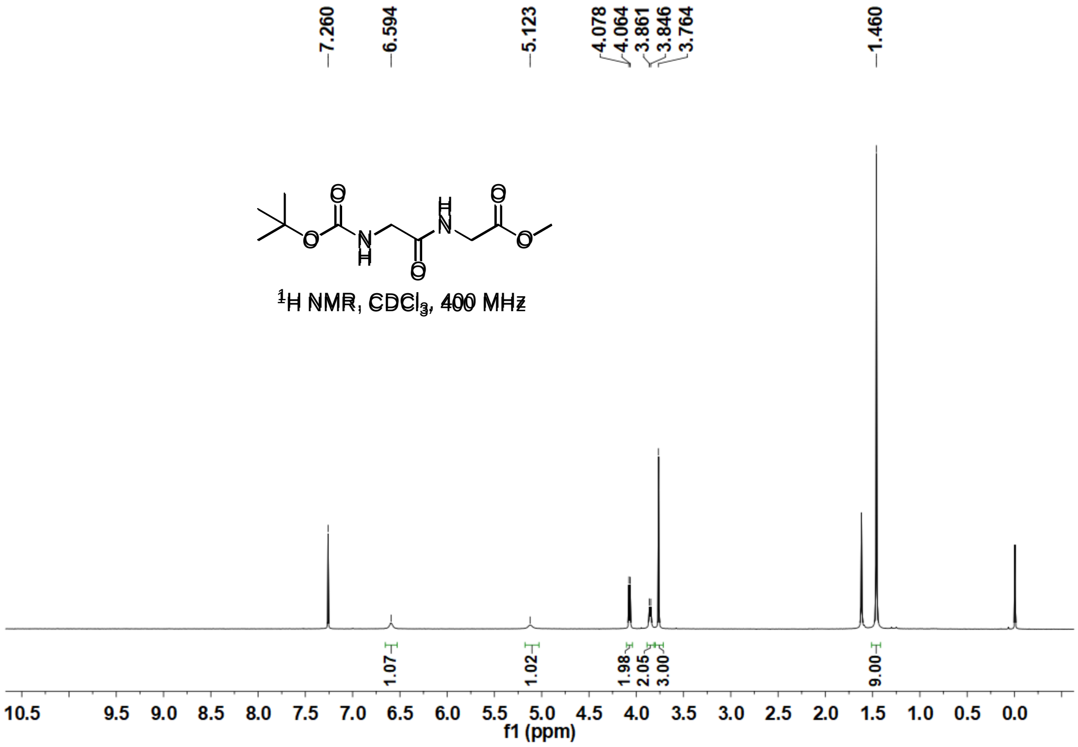

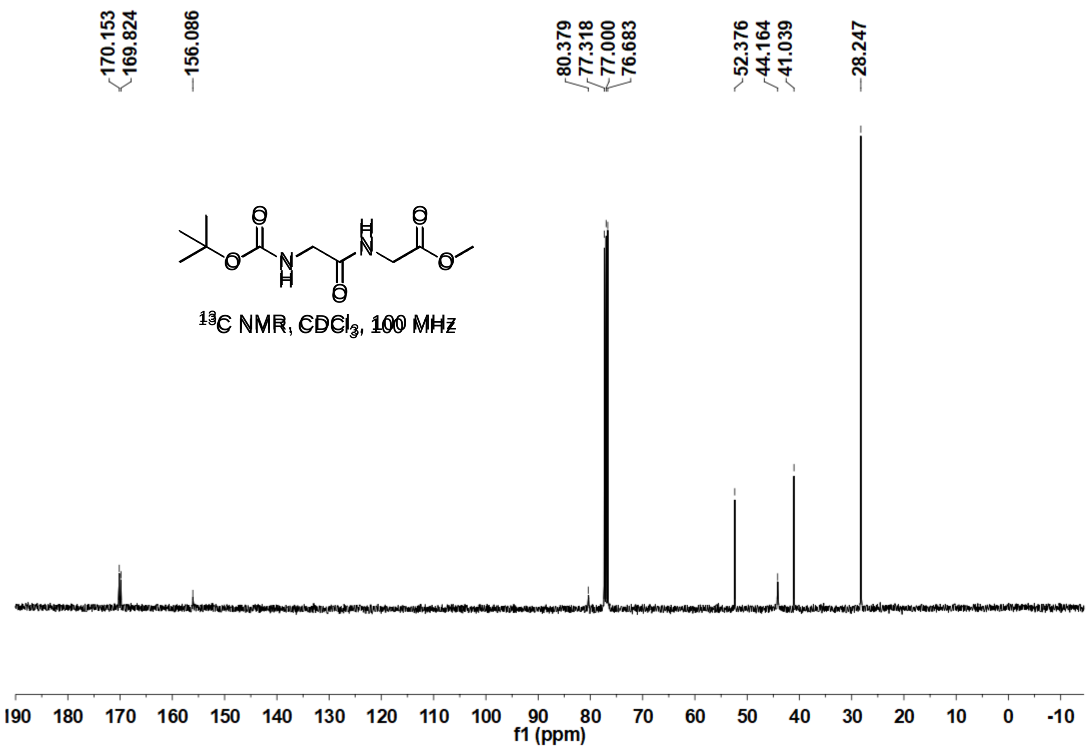

Boc-L-Leu-L-Lys(Z)-OMe (3-3)

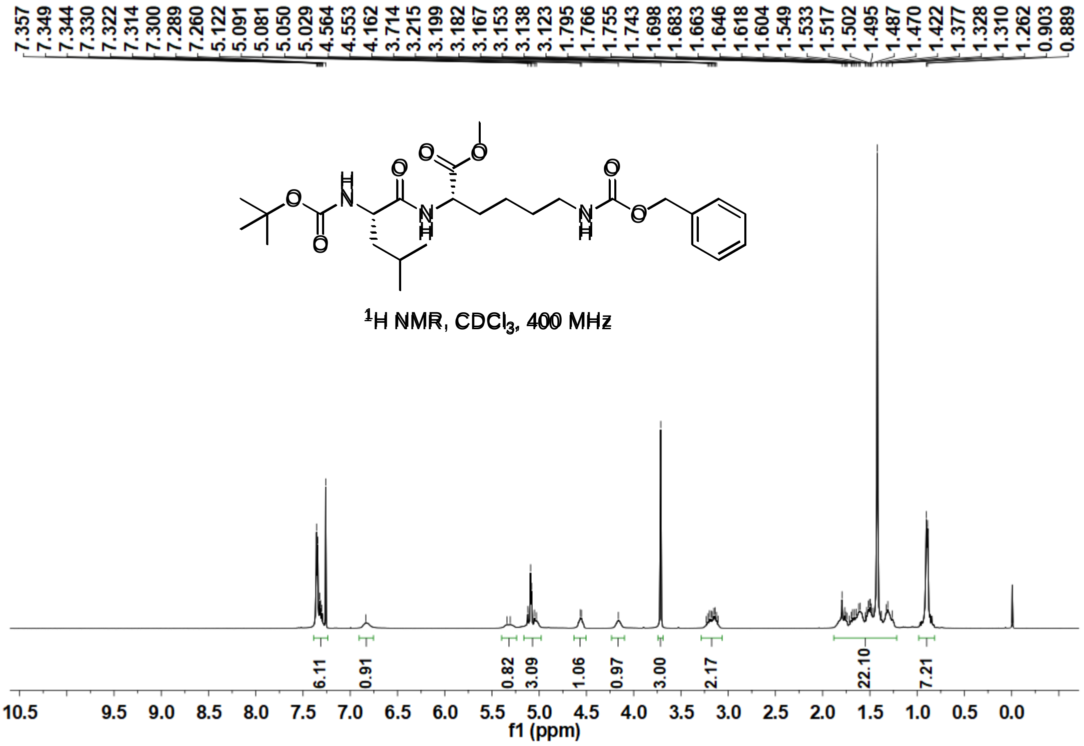

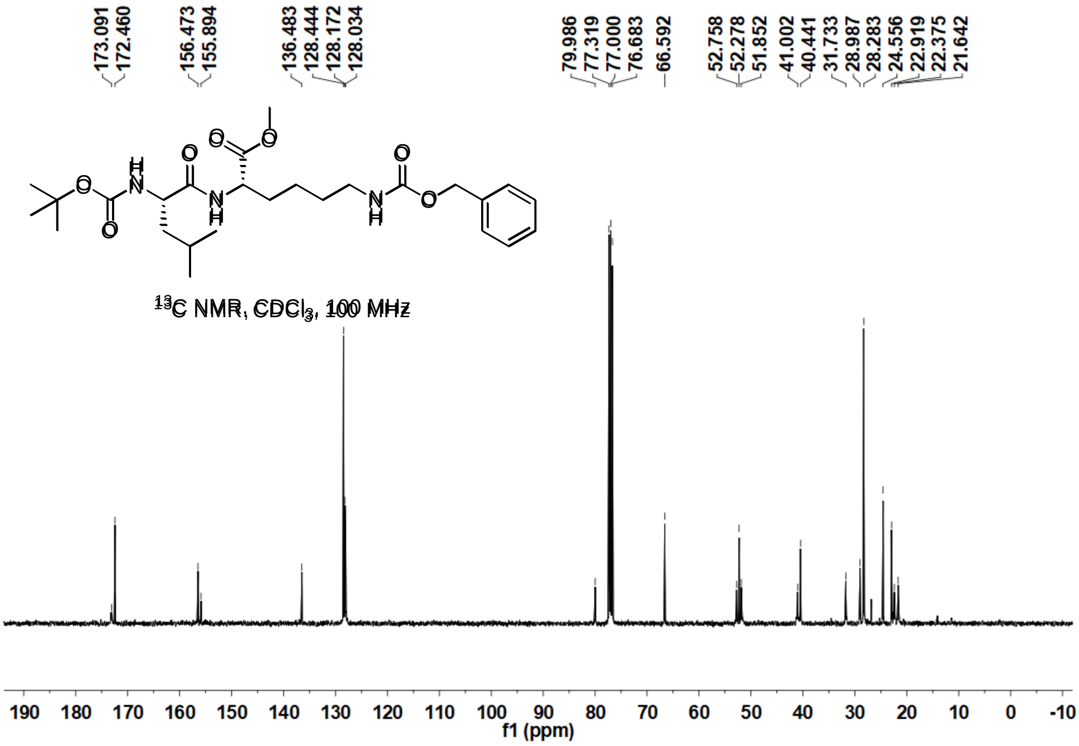
173.091
172.460
156.473
155.894
136.483
128.444
128.172
128.034
79.986
77.319
77.000
76.683
66.592
52.758
52.278
51.852
41.002
40.441
31.733
28.987
28.283
24.556
22.919
22.375
21.642
^{13}C NMR, $CDCl_3$, 100 MHz
190 180 170 160 150 140 130 120 110 100 90 80 70 60 50 40 30 20 10 0 -10
f1 (ppm)

Boc-L-Leu-L-Ala-OMe (3-4)

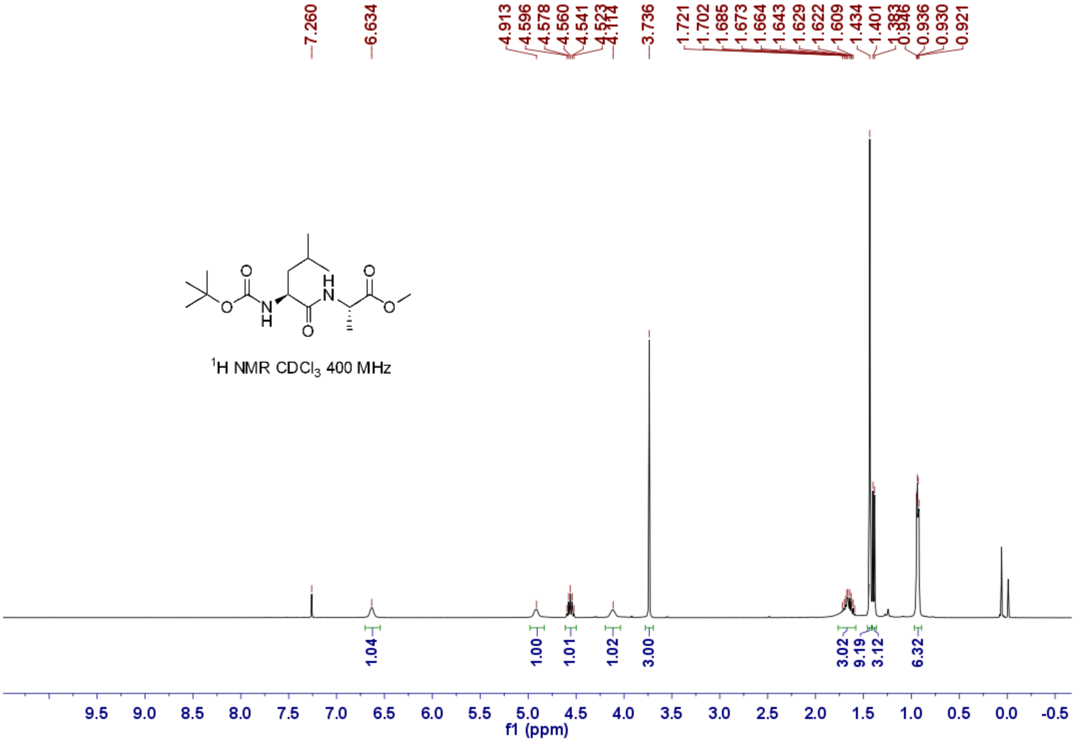

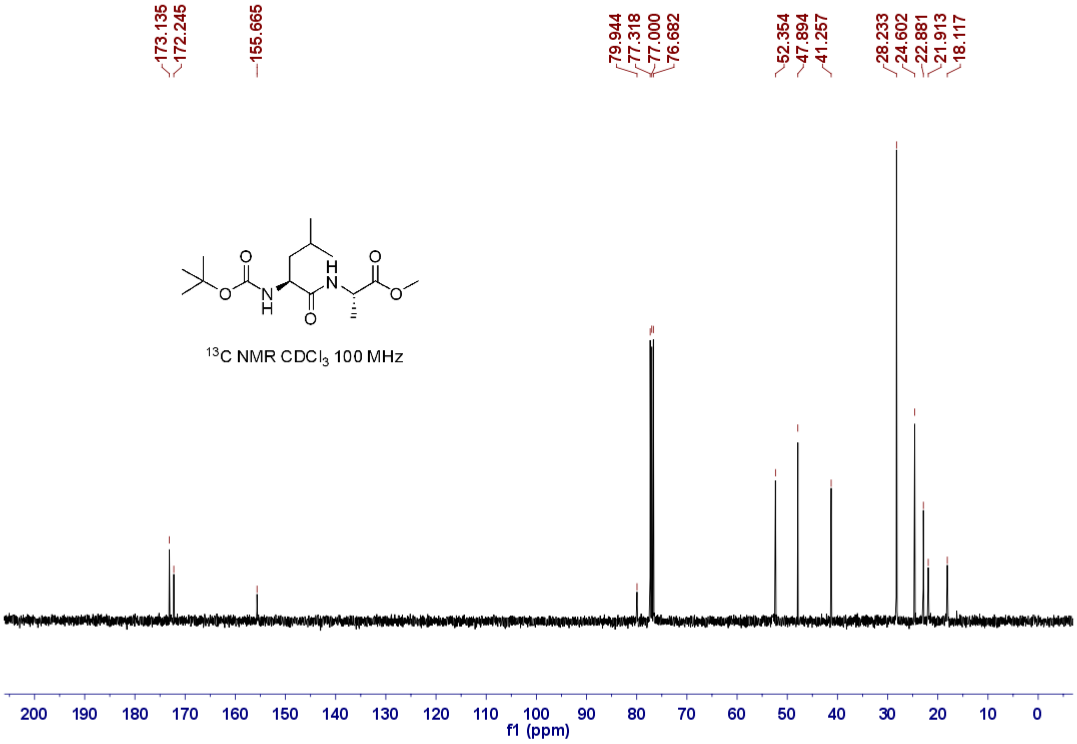

Cbz-L-Leu-L-Ala-OMe (3-5)

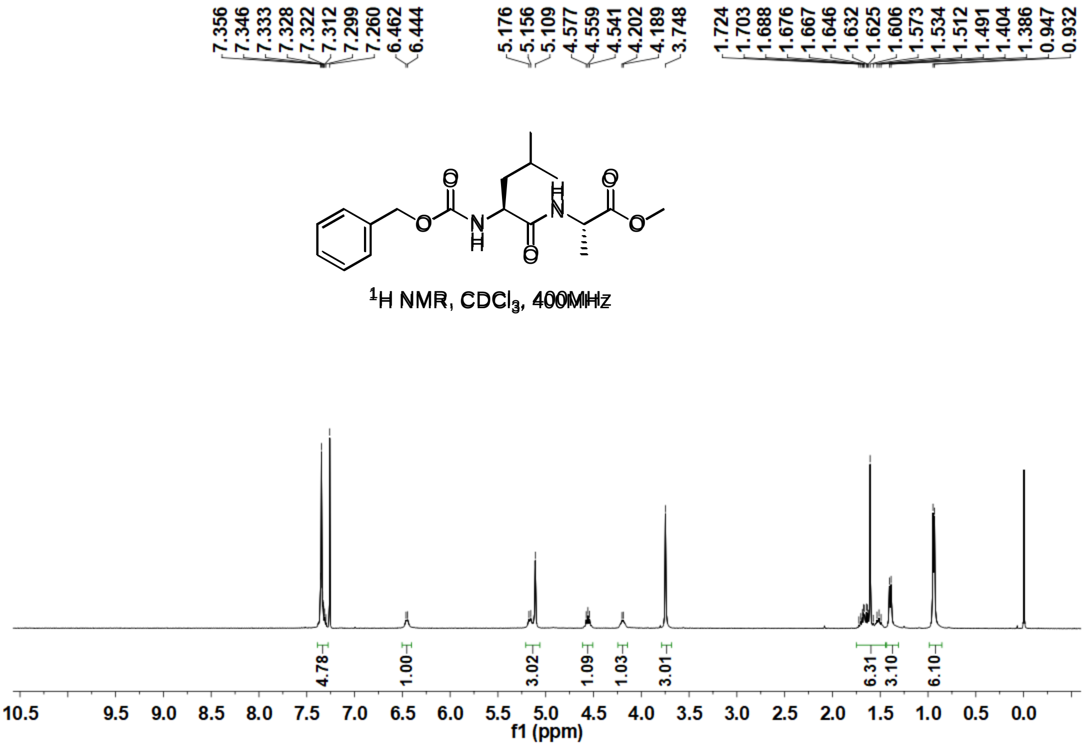

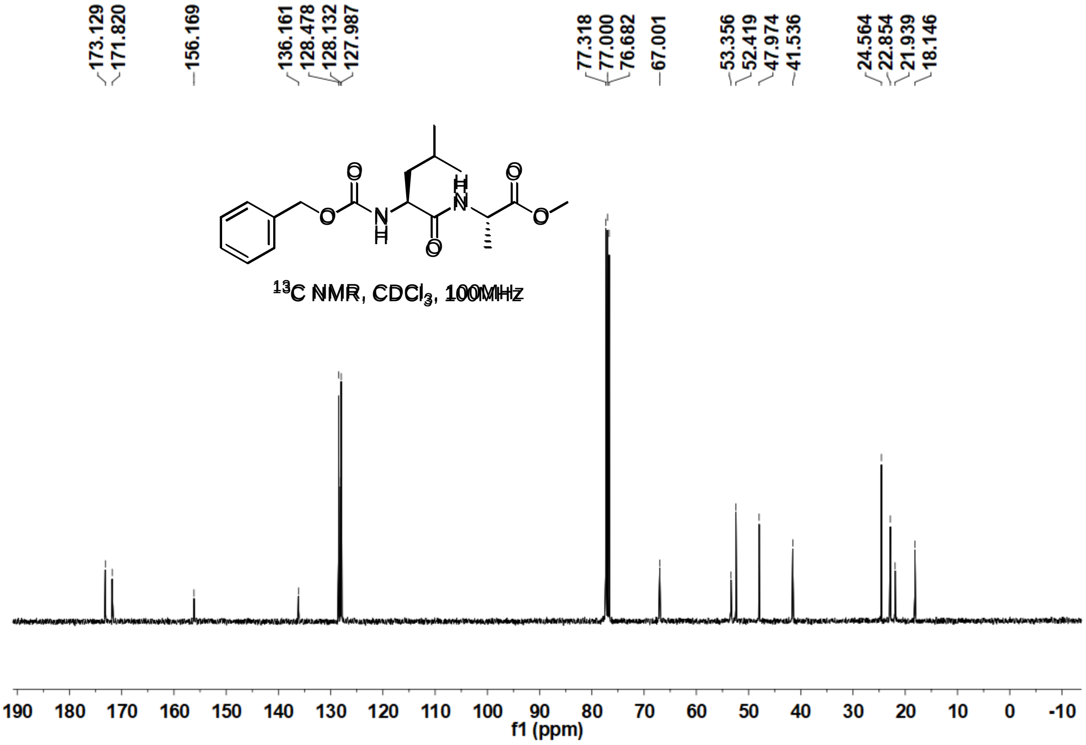

Fmoc-L-Leu-L-Ala-OMe (3-6)

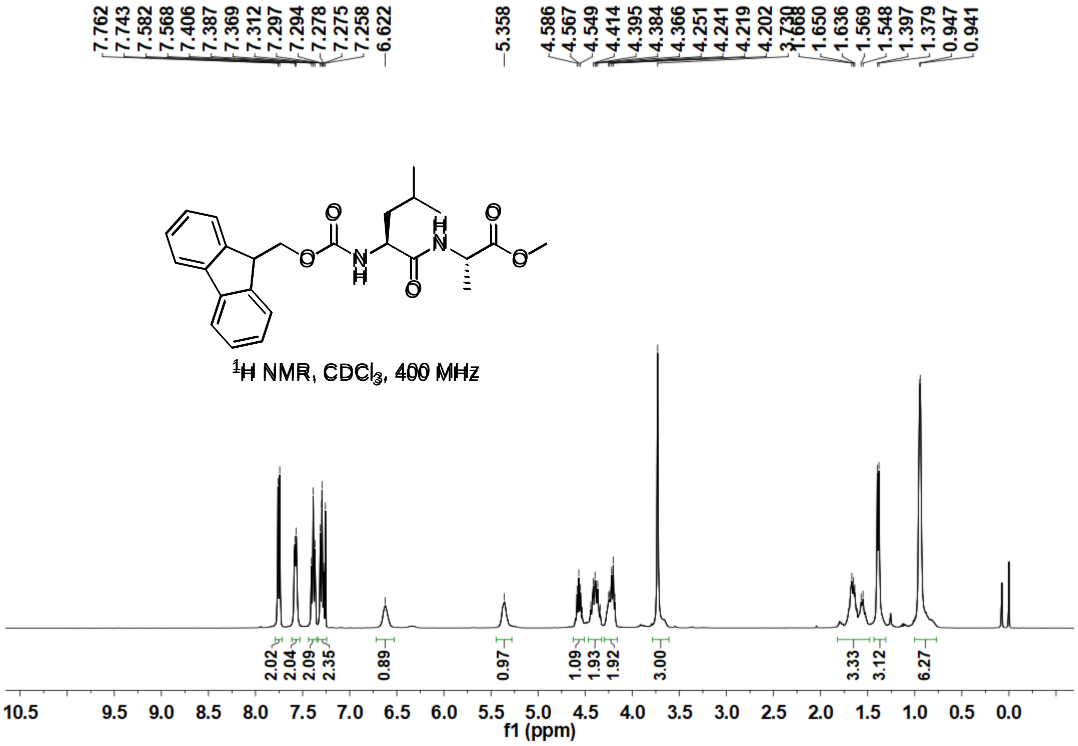

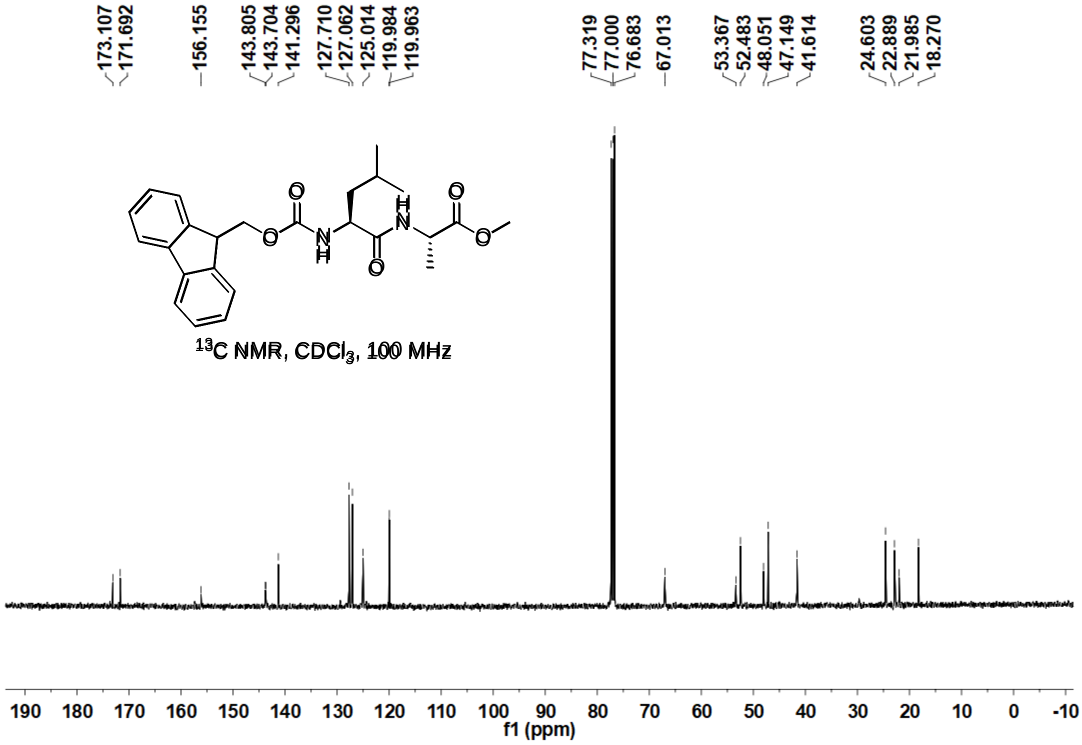

Cbz-L-Leu-L-Lys(Z)-OMe (3-7)

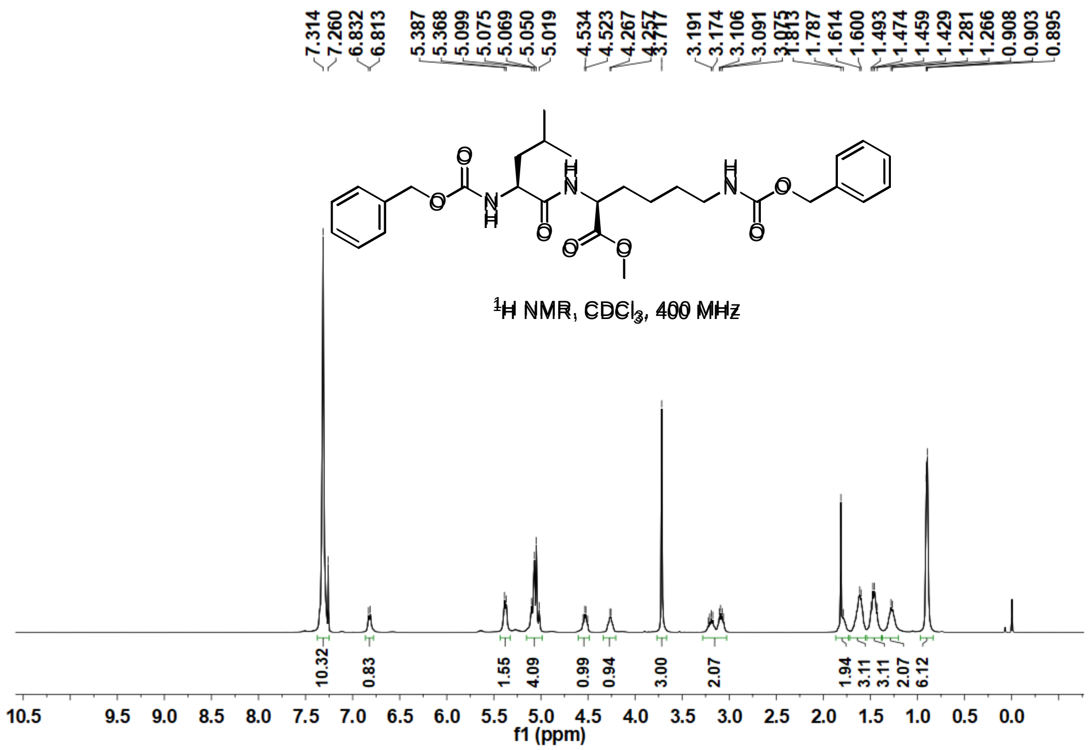

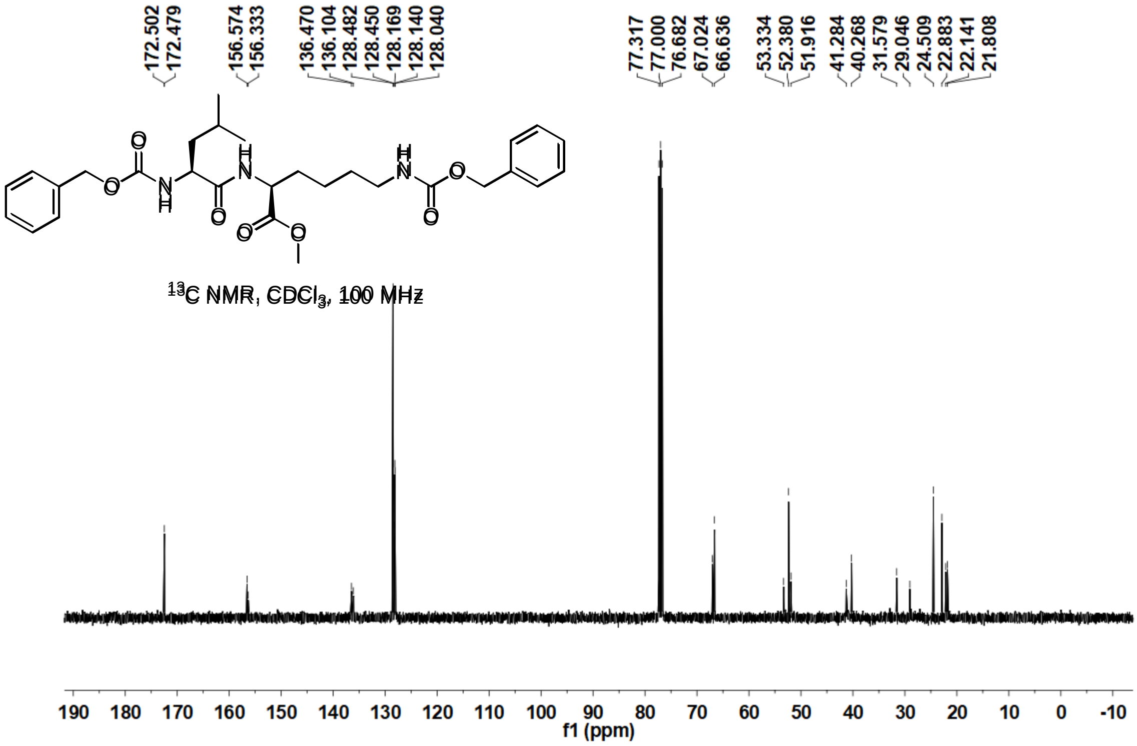

Cbz-L-Ala-L-His(Trt)-OMe (3-8)

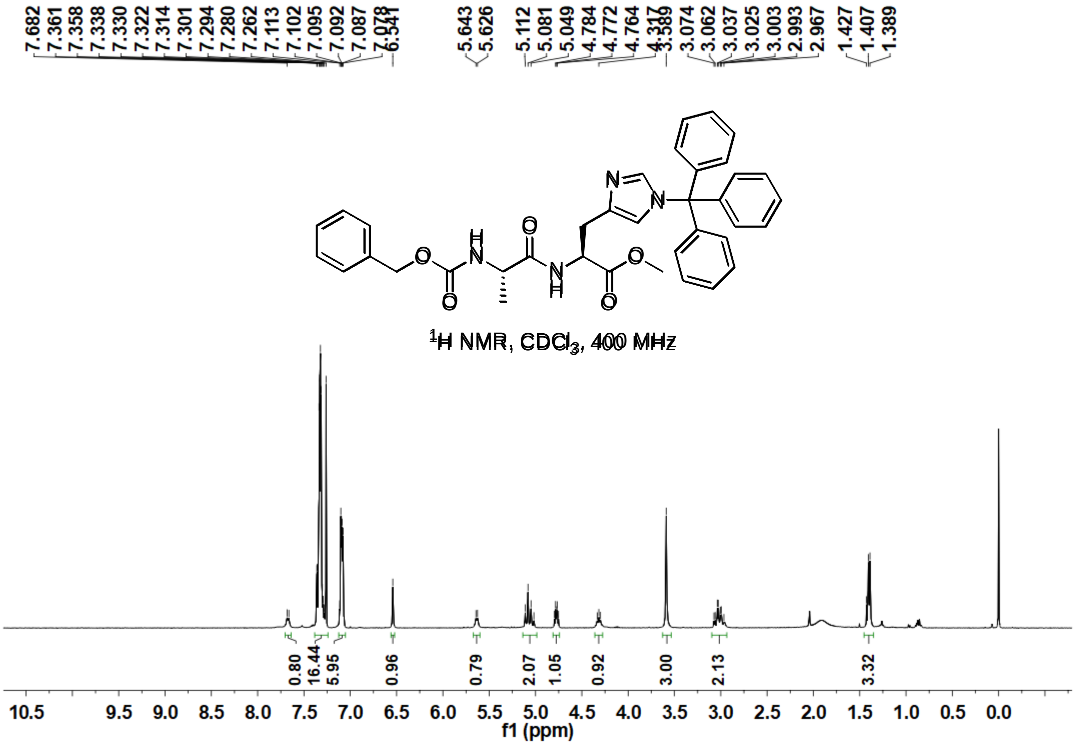

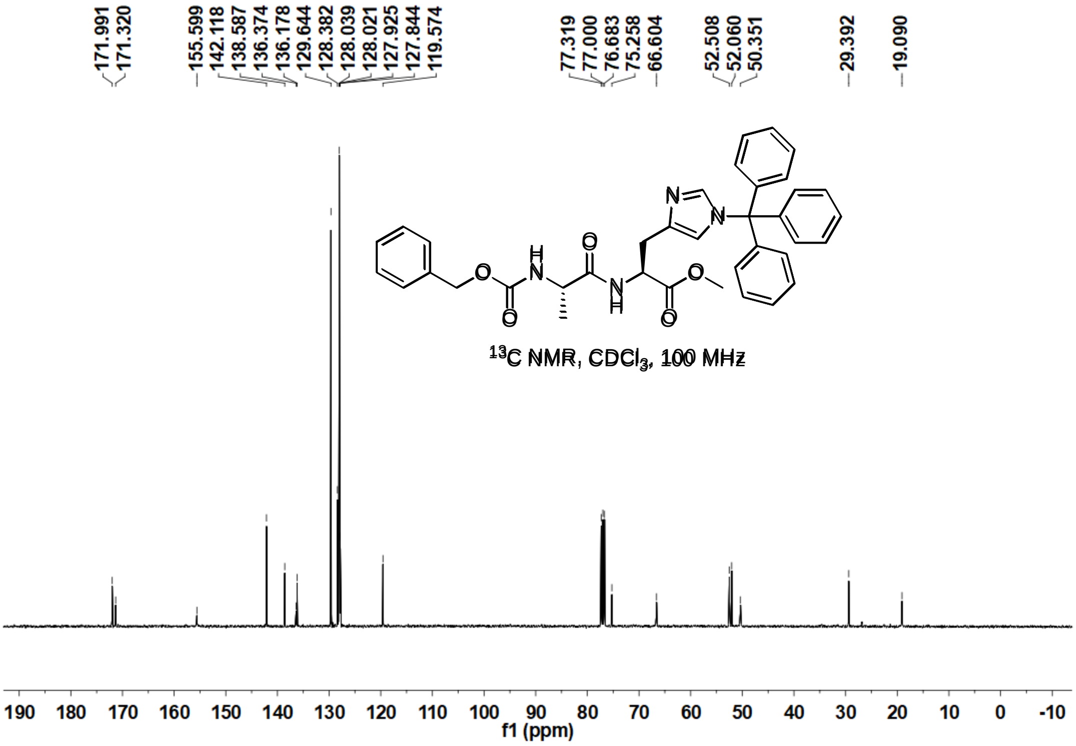

Cbz-L-Ala-L-Cys(Trt)-OMe (3-9)

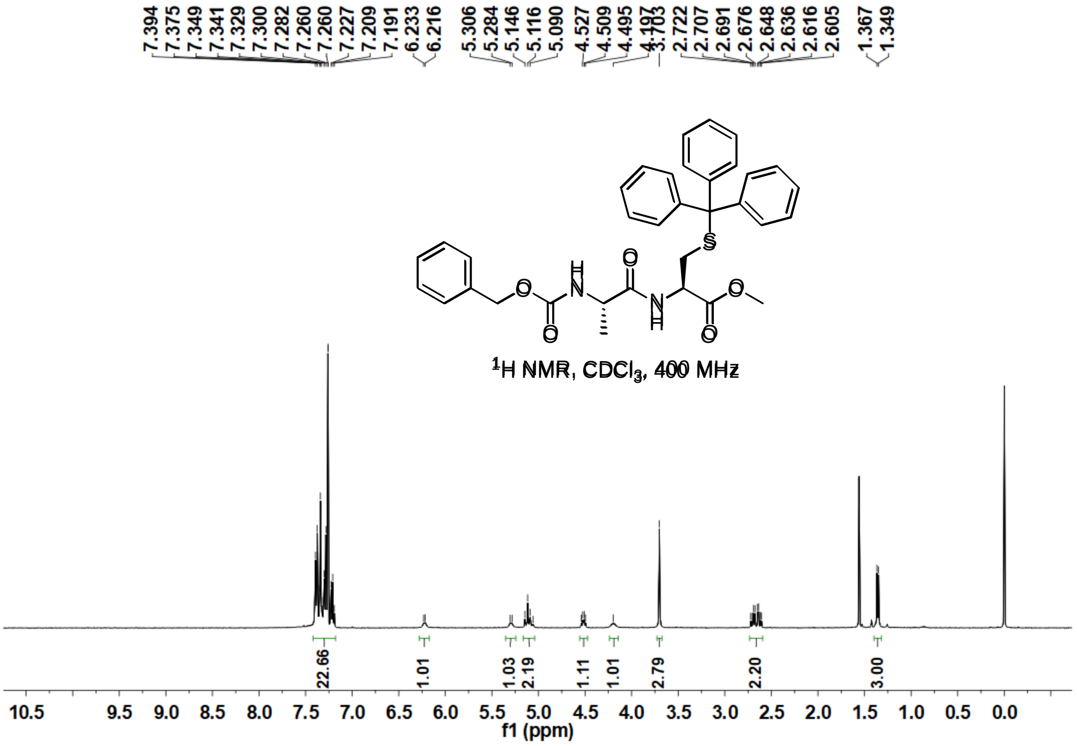

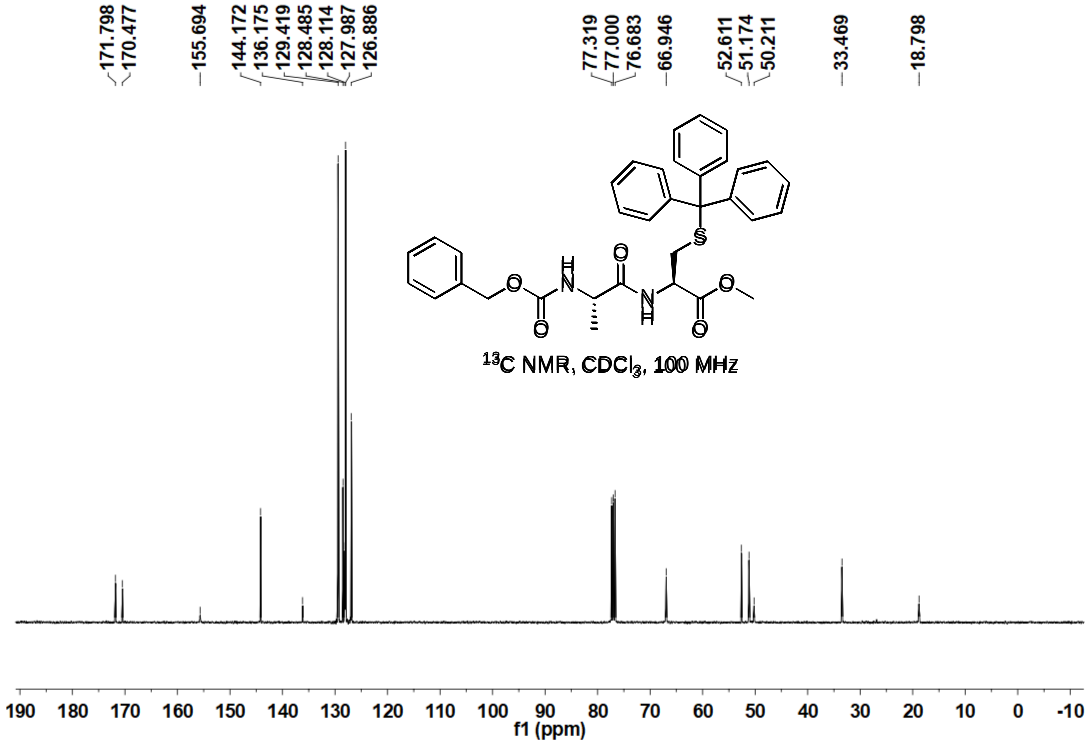

Cbz-L-Ala-L-Pro-OMe (3-10)

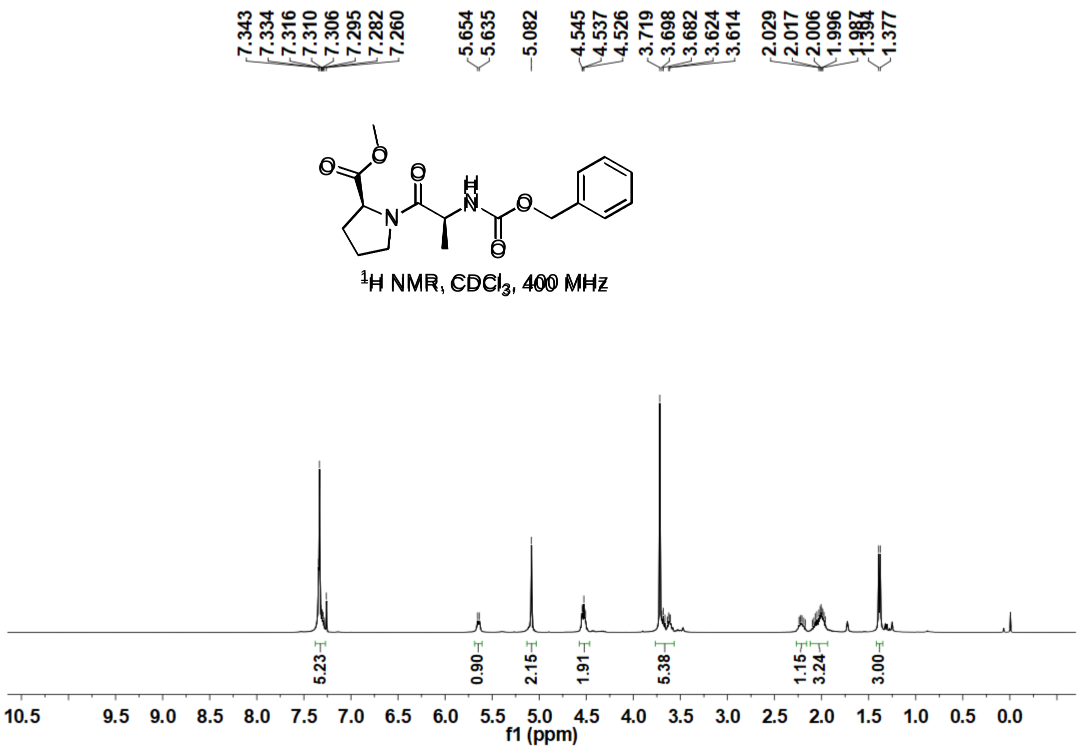

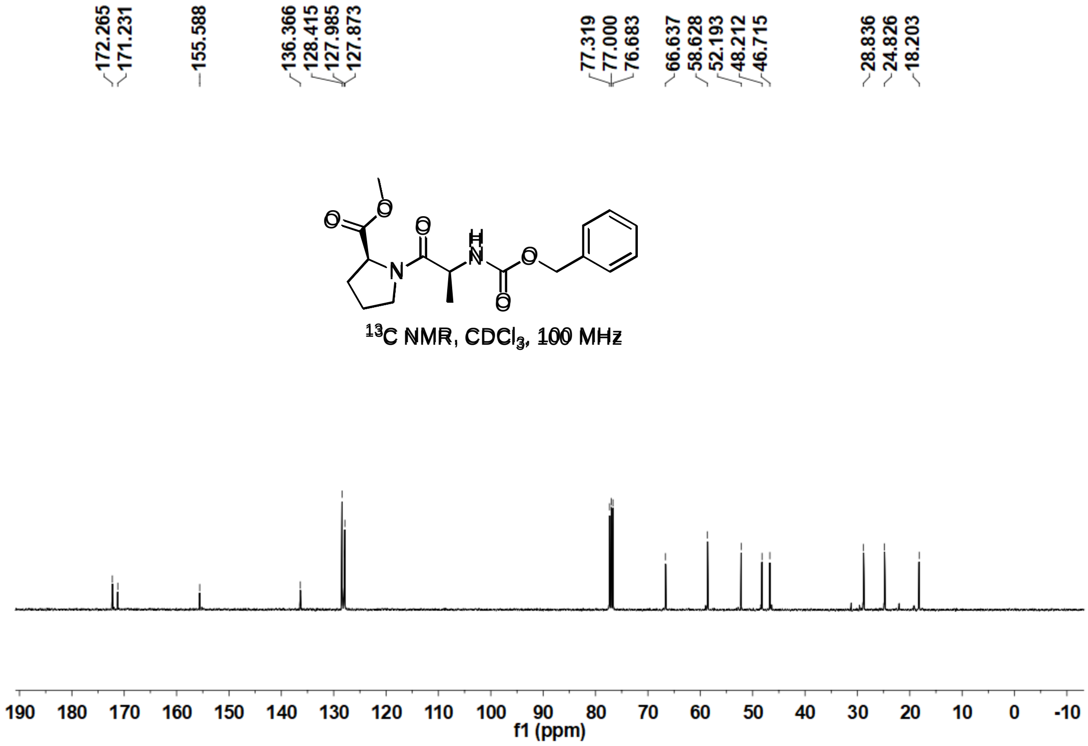

Cbz-L-Met-Gly-OMe (3-11)

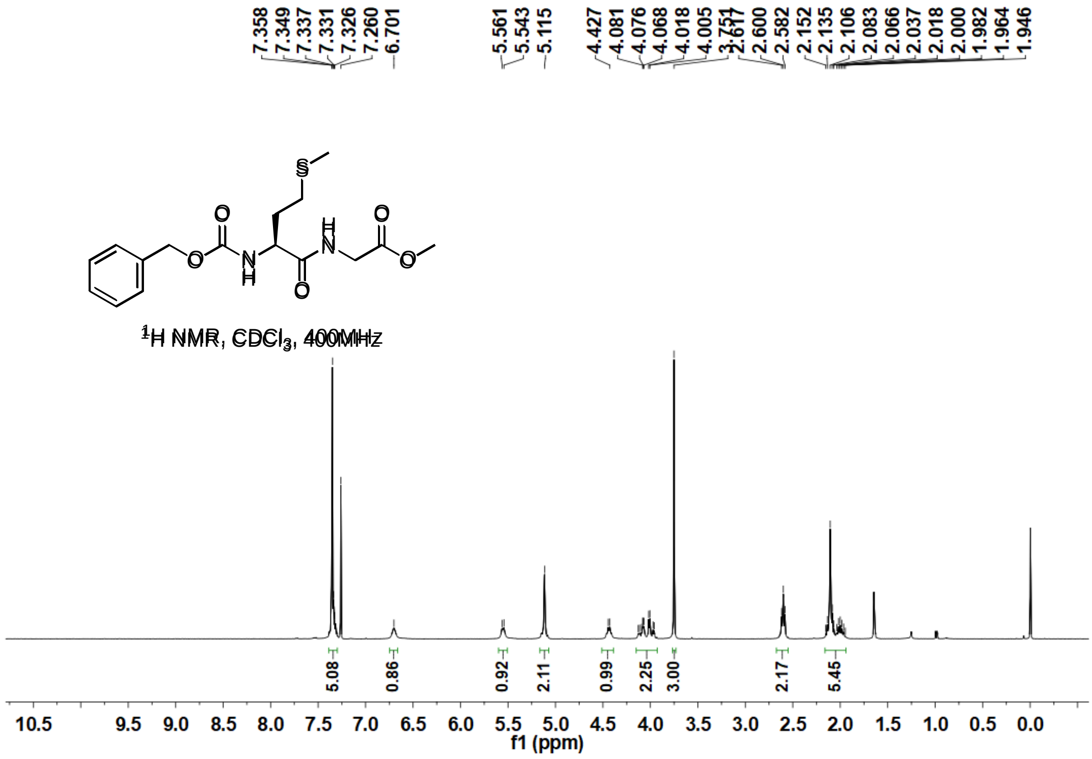
7.358
7.349
7.337
7.331
7.326
7.260
6.701
5.561
5.543
5.115
4.427
4.081
4.076
4.068
4.018
4.005
3.751
2.617
2.600
2.582
2.152
2.135
2.106
2.083
2.066
2.037
2.018
2.000
1.982
1.964
1.946
^{1}H NMR, $CDCl_3$, 400MHz
5.08
0.86
0.92
2.11
0.99
2.25
3.00
2.17
5.45
f1 (ppm)

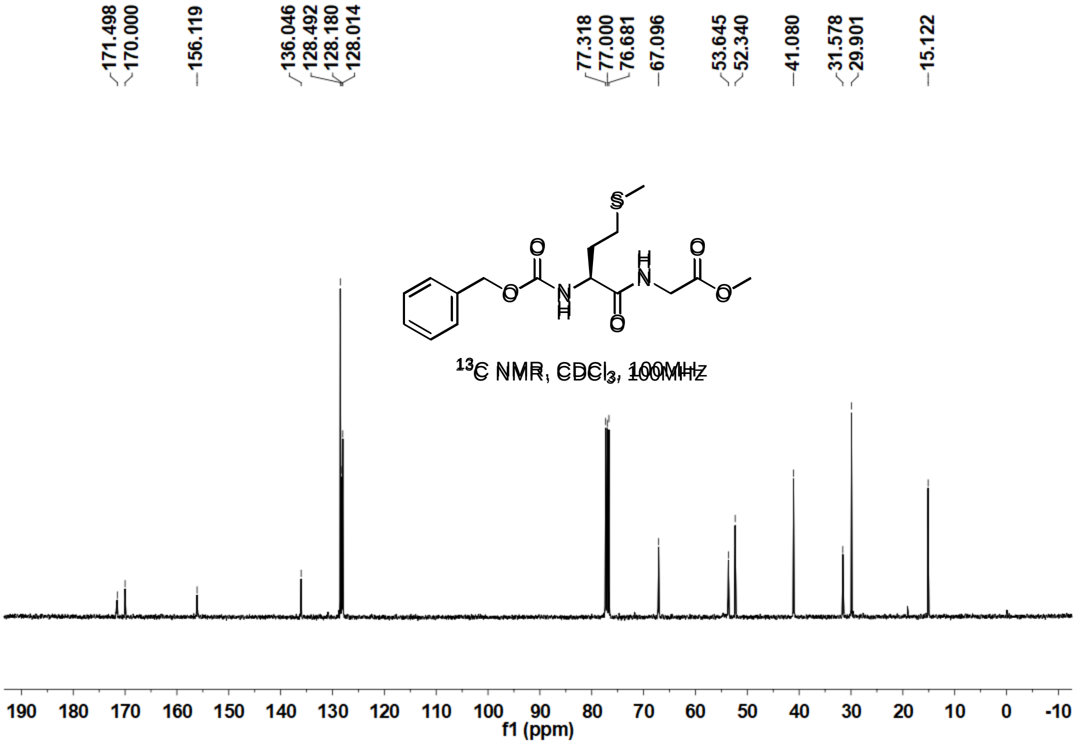
171.498
170.000
156.119
136.046
128.492
128.180
128.014
77.318
77.000
76.681
67.096
53.645
52.340
41.080
31.578
29.901
15.122
^{13}C NMR, $CDCl_3$, 100MHz
f1 (ppm)

Cbz-L-Met-Gly-OEt (3-12)

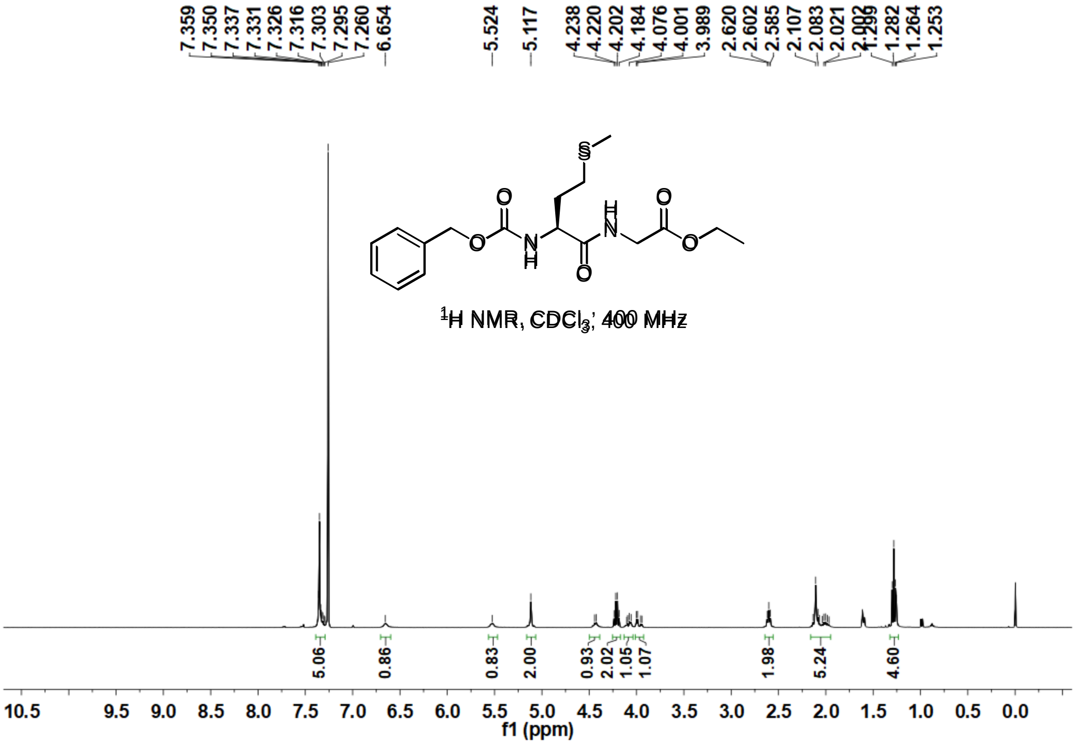

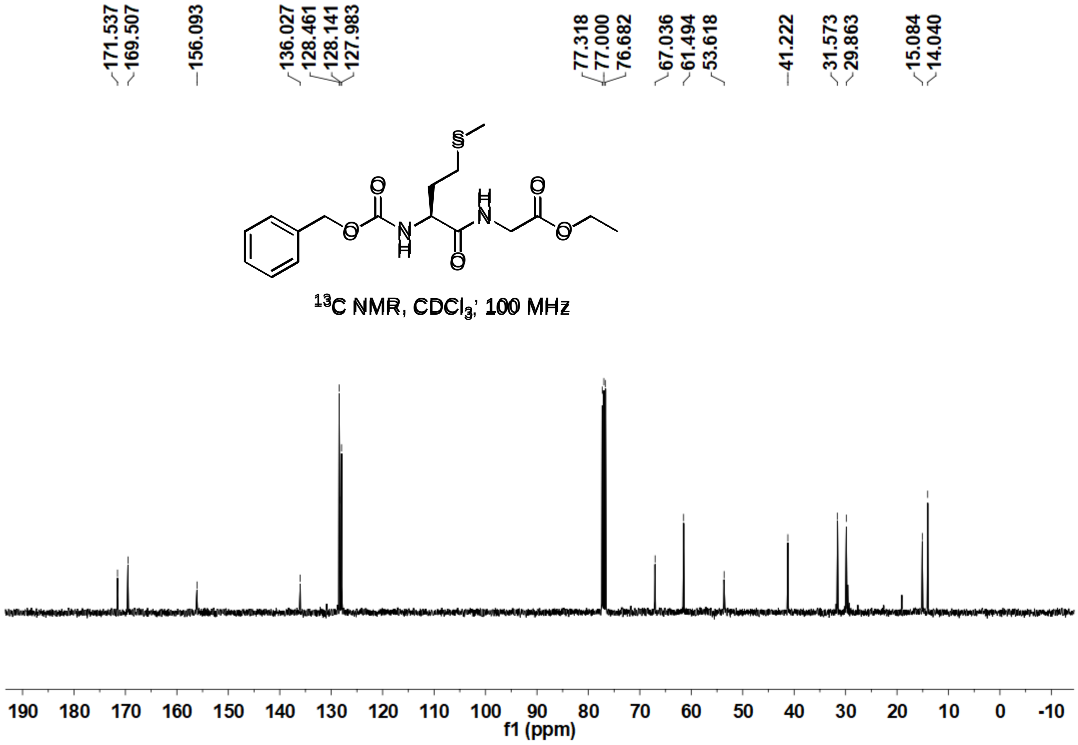

Cbz-L-Trp-L-Leu-OMe (3-13)

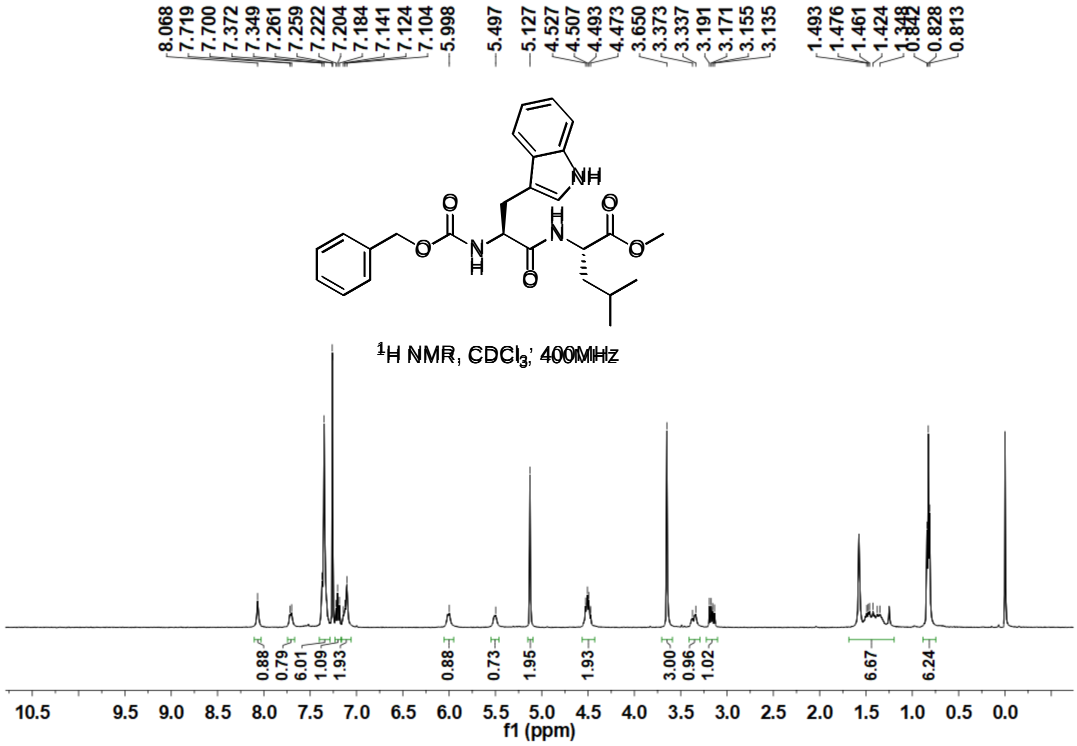

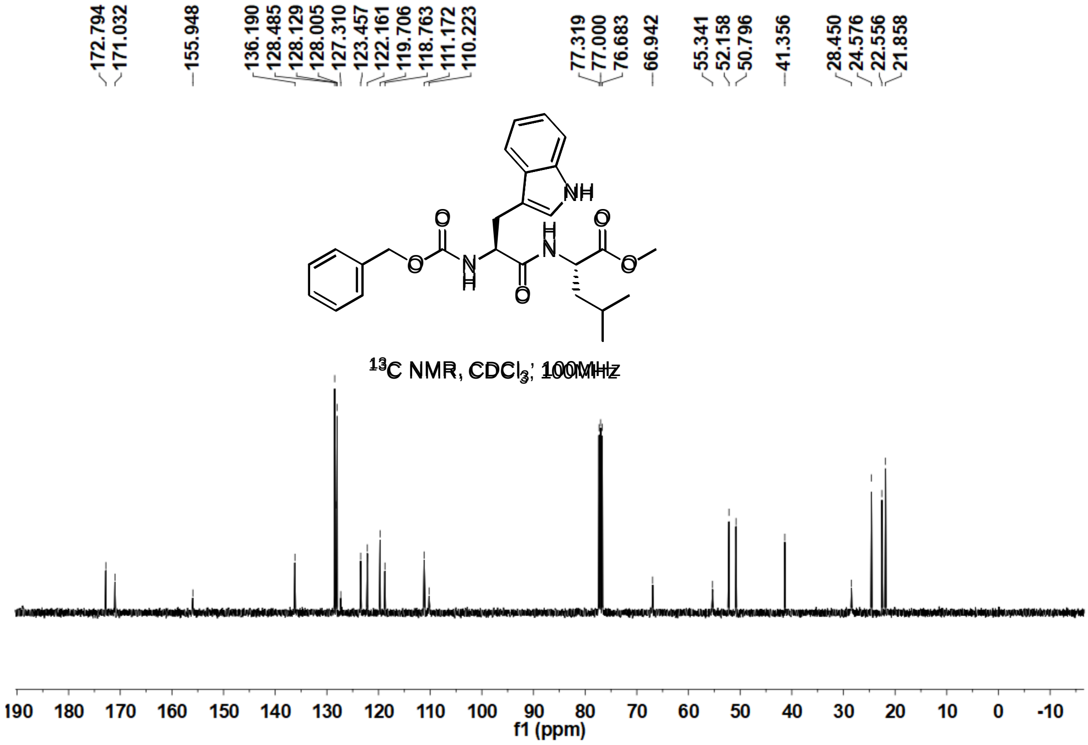

Cbz-L-Asn(Trt)-L-Leu-OMe (3-14)

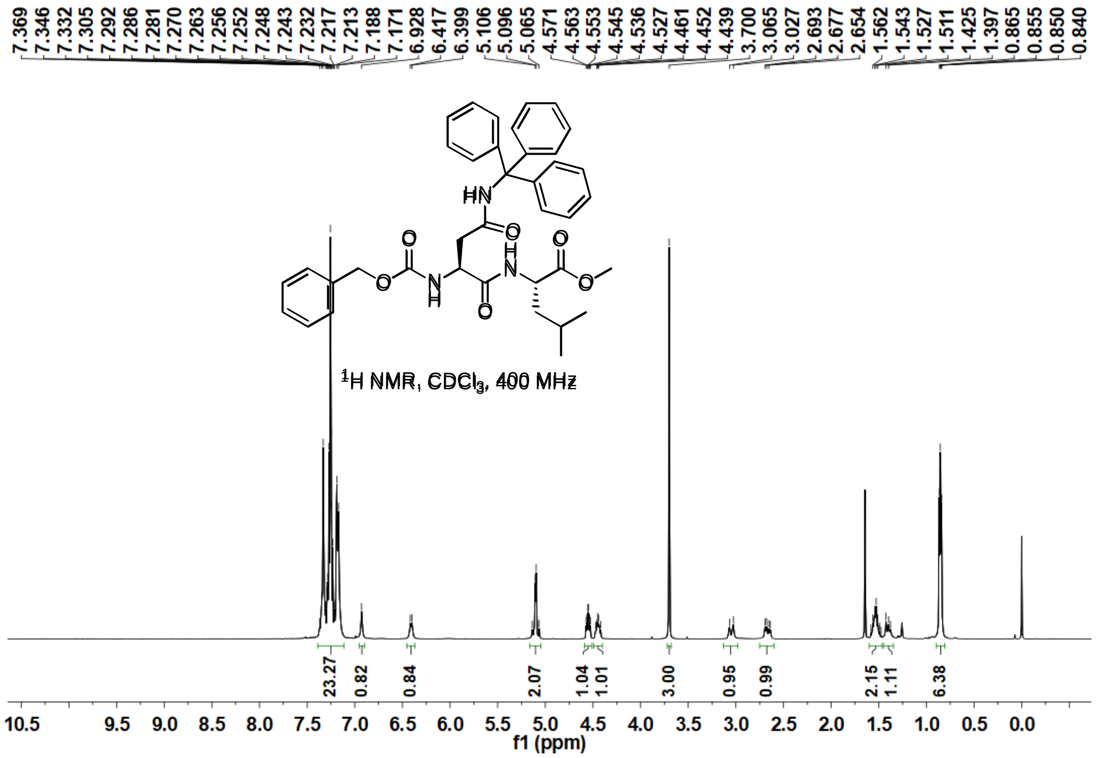

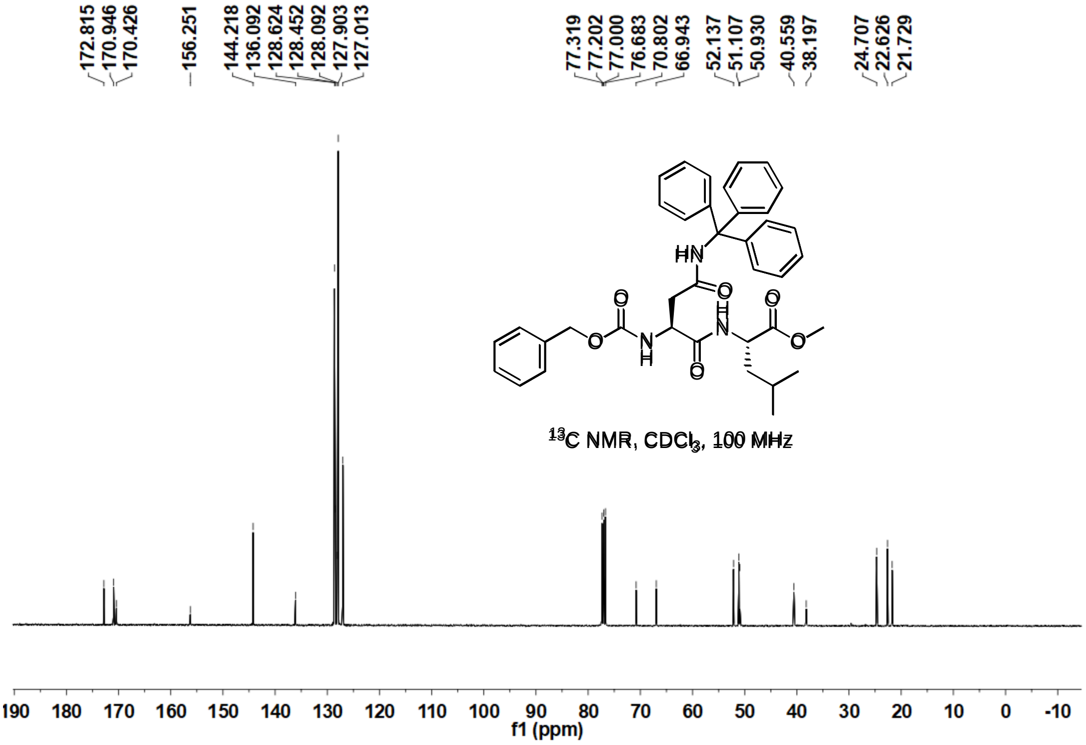

Cbz-L-Phe-L-Leu-OMe (3-15)

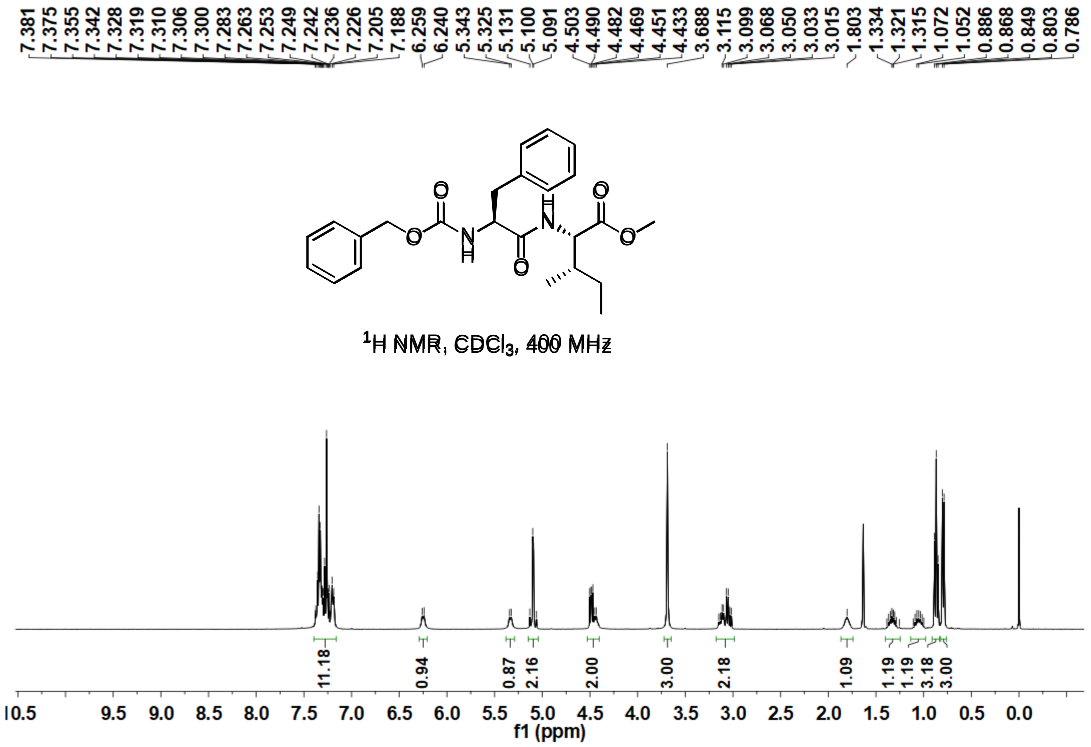

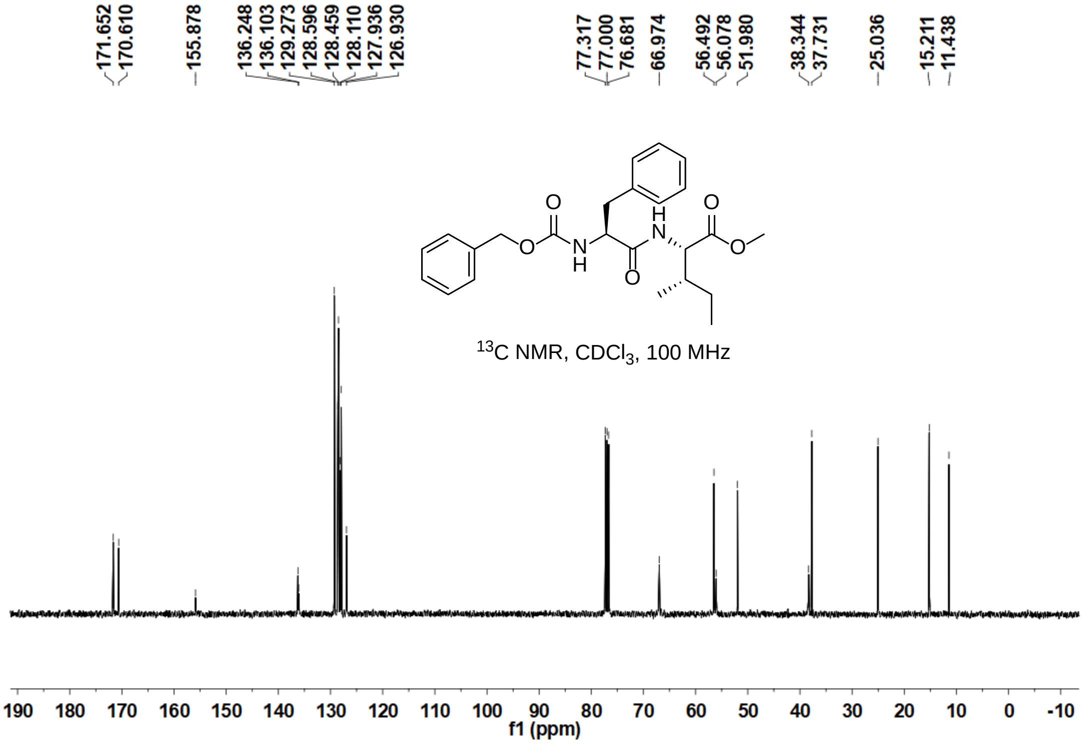

Cbz-L-Phe-L-Tyr(Bzl)-OMe (3-16)

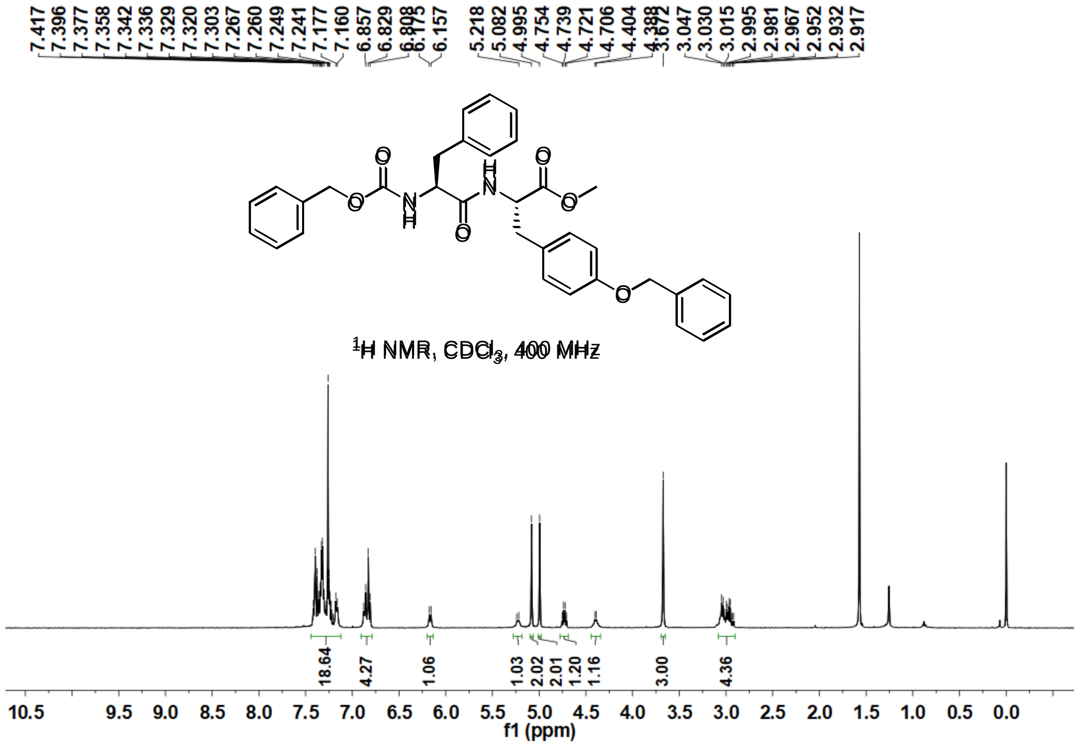

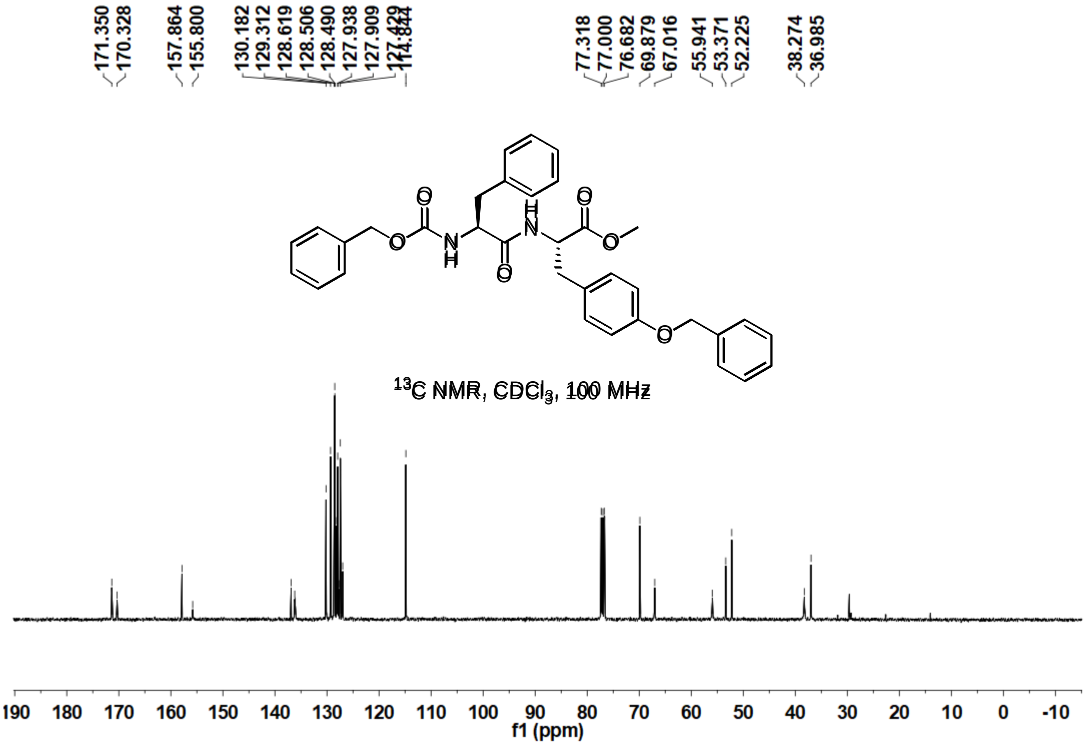

Cbz-L-Phe-L-Ser-OMe (3-17)

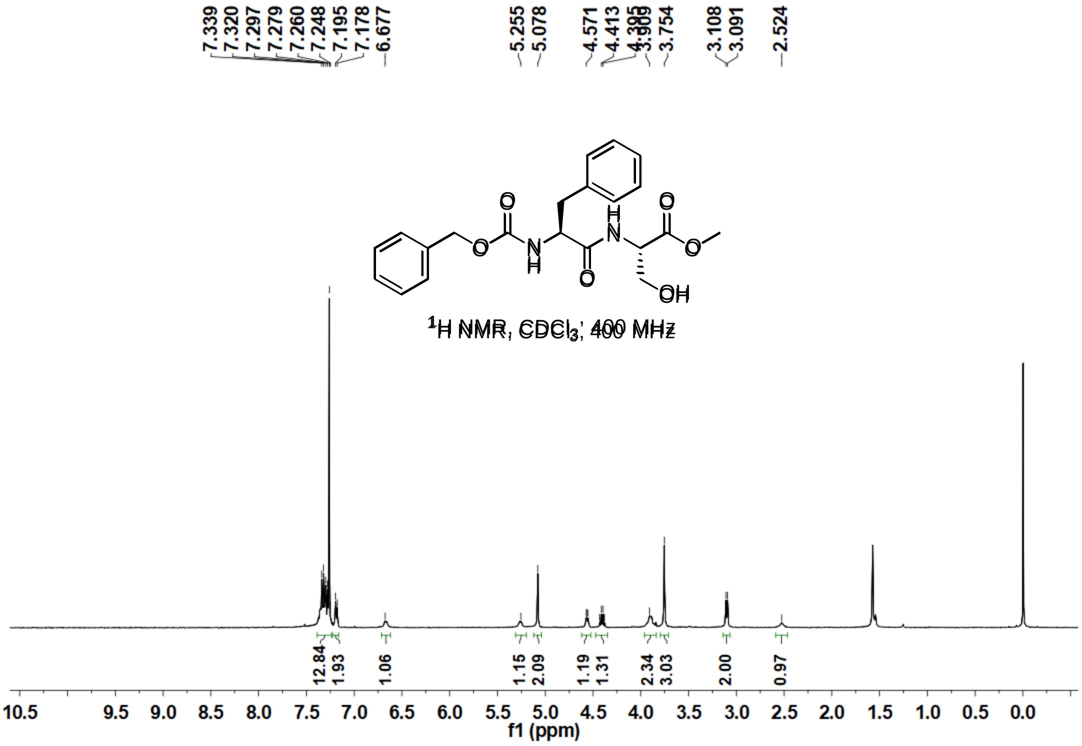

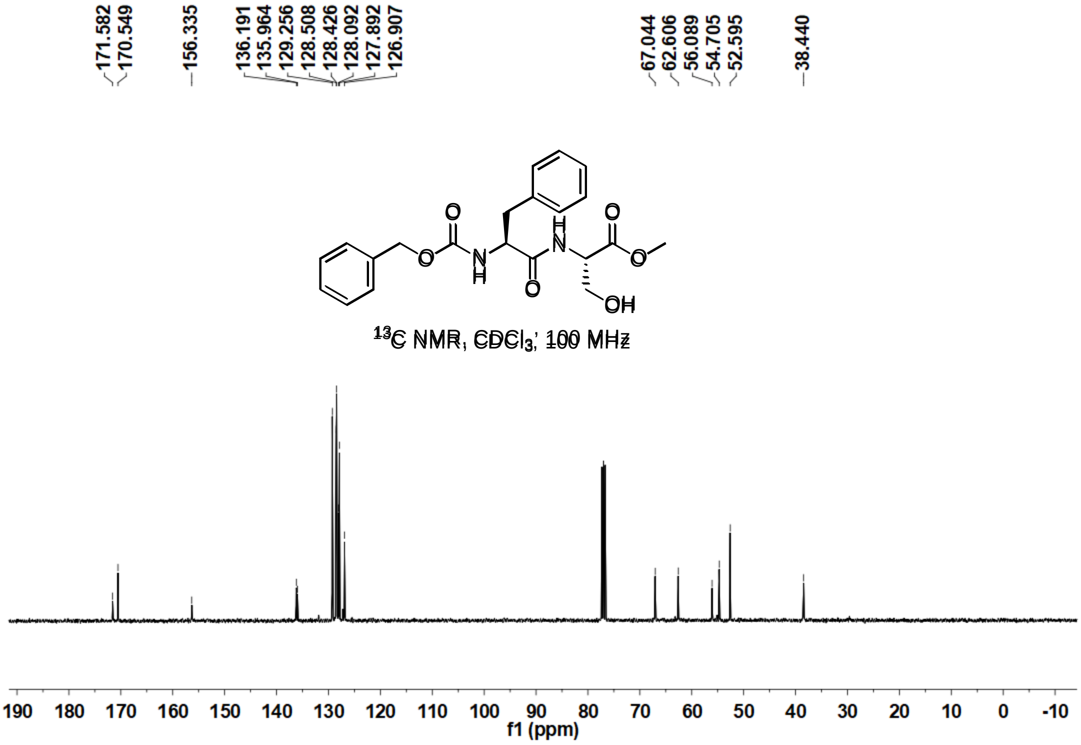

Cbz-L-Phe-L-Thr-OMe (3-18)

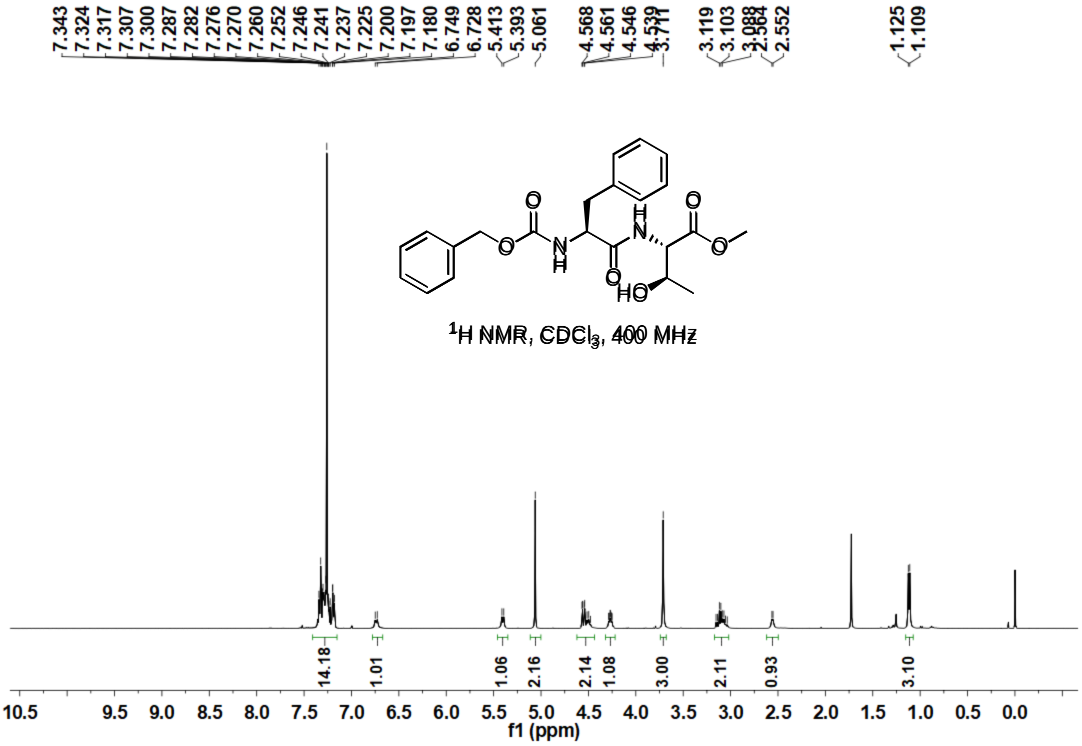

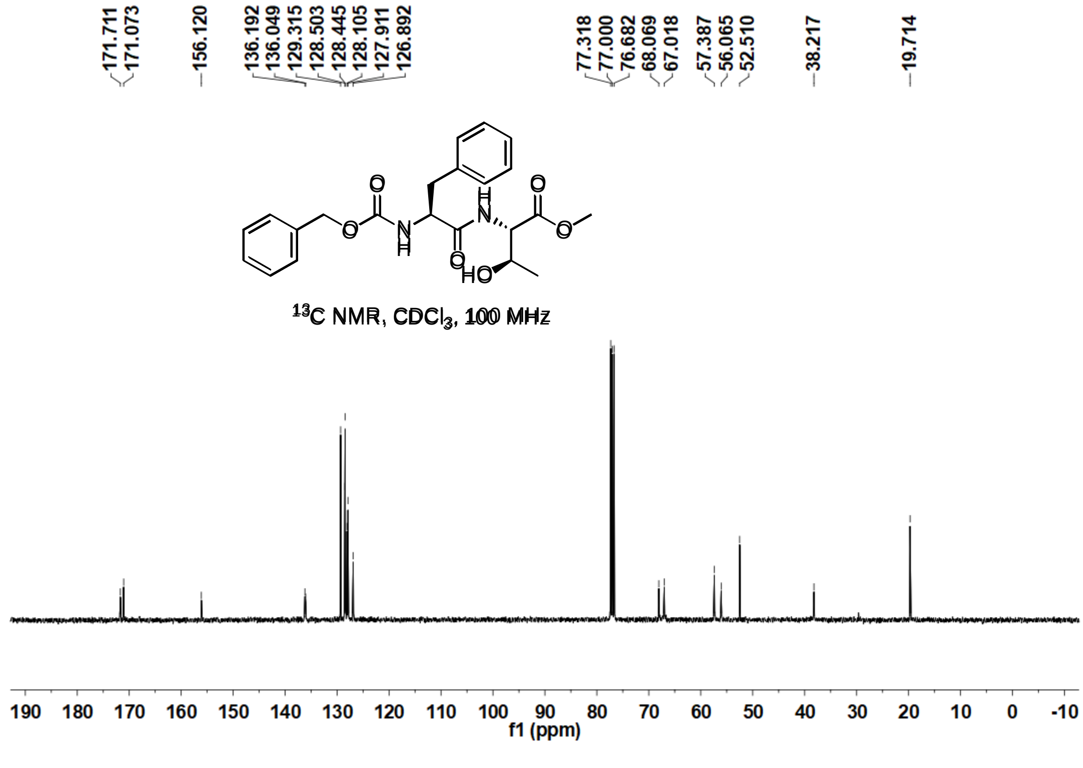

Cbz-L-Phe-L-Tyr-OMe (3-19)

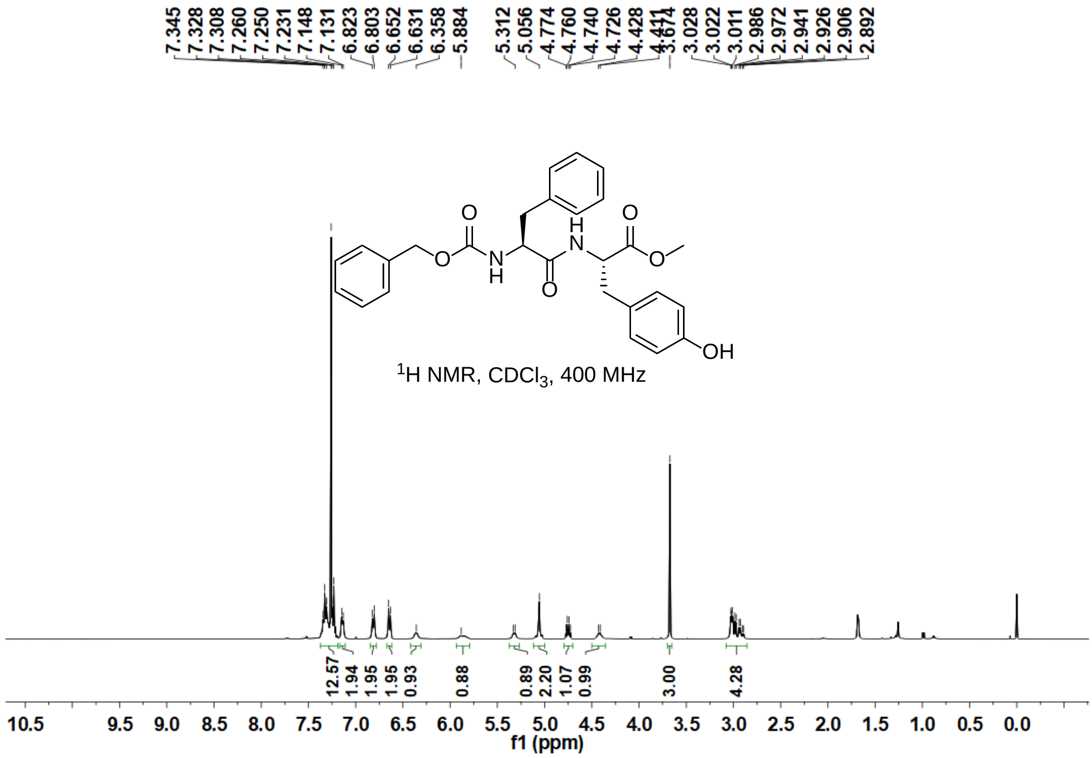
7.345 7.328 7.308 7.260 7.250 7.231 7.148 7.131 6.823 6.803 6.652 6.631 6.358 5.884 5.312 5.056 4.774 4.760 4.740 4.726 4.428 4.411 3.674 3.028 3.022 3.011 2.986 2.972 2.941 2.926 2.906 2.892
O
NH
H
N
O
O
O
OH
^{1}H NMR, CDCl$_3$, 400 MHz
12.57 1.94 1.95 1.95 0.93 0.88 0.89 2.20 1.07 0.99 3.00 4.28
10.5 9.5 9.0 8.5 8.0 7.5 7.0 6.5 6.0 5.5 5.0 4.5 4.0 3.5 3.0 2.5 2.0 1.5 1.0 0.5 0.0
f1 (ppm)

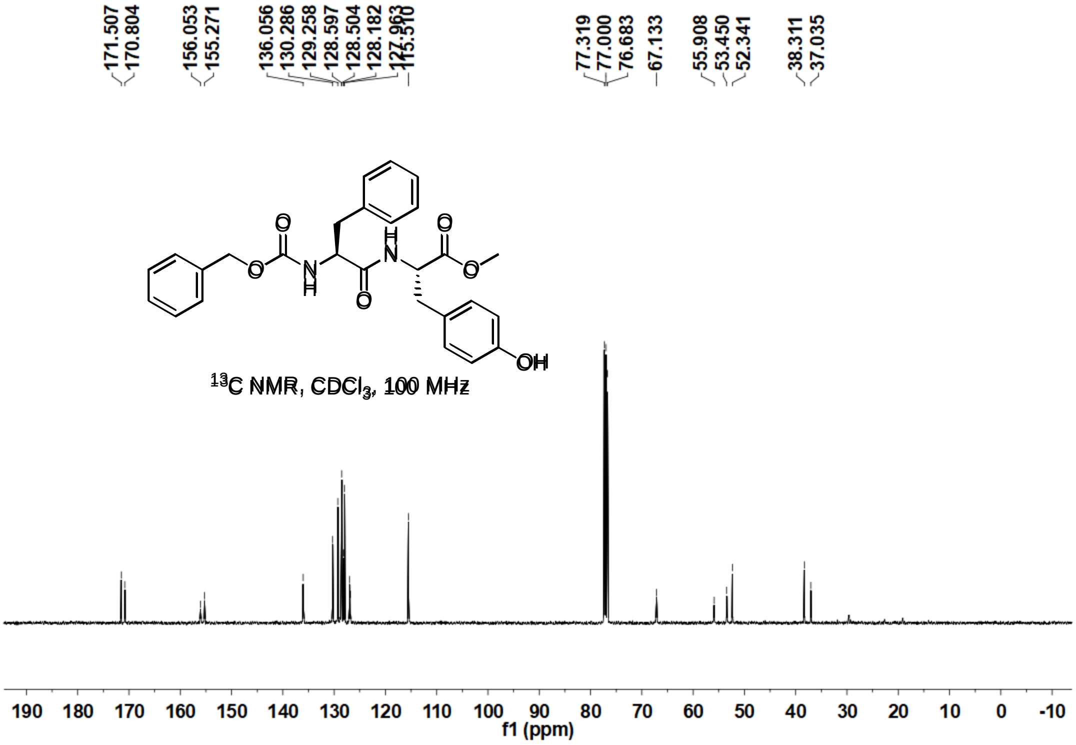
171.507 170.804 156.053 155.271 136.056 130.286 129.258 128.597 128.504 128.182 127.963 115.510 77.319 77.000 76.683 67.133 55.908 53.450 52.341 38.311 37.035
O
NH
H
N
O
O
O
OH
^{13}C NMR, CDCl$_3$, 100 MHz
190 180 170 160 150 140 130 120 110 100 90 80 70 60 50 40 30 20 10 0 -10
f1 (ppm)

Boc-L-Val-L-Val-OMe (3-20)

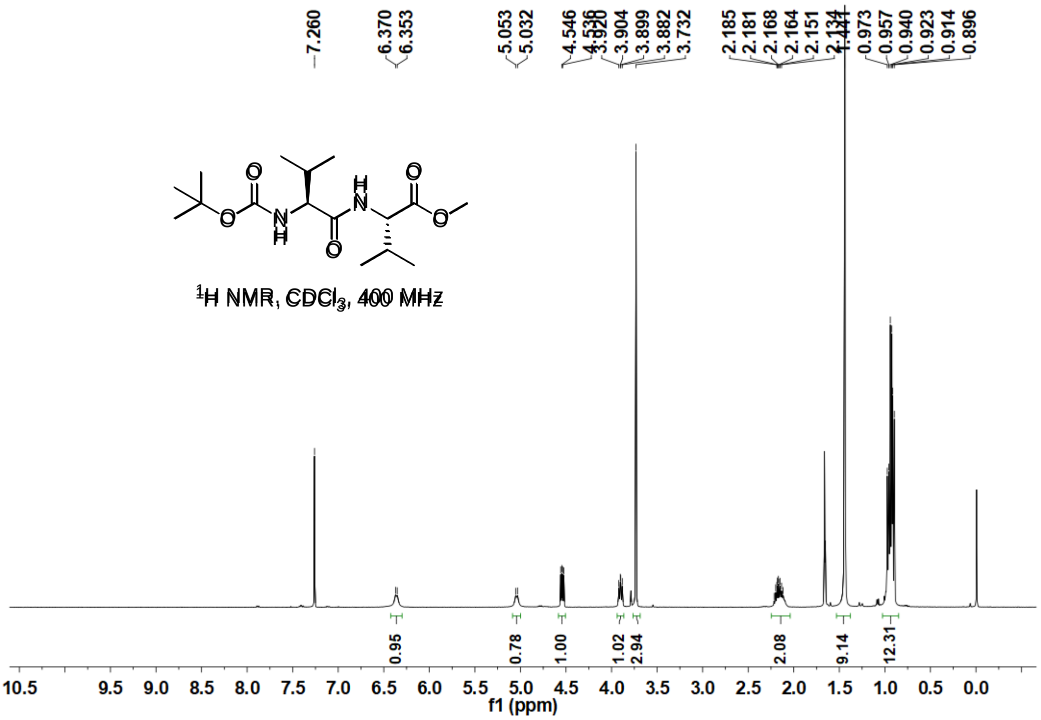

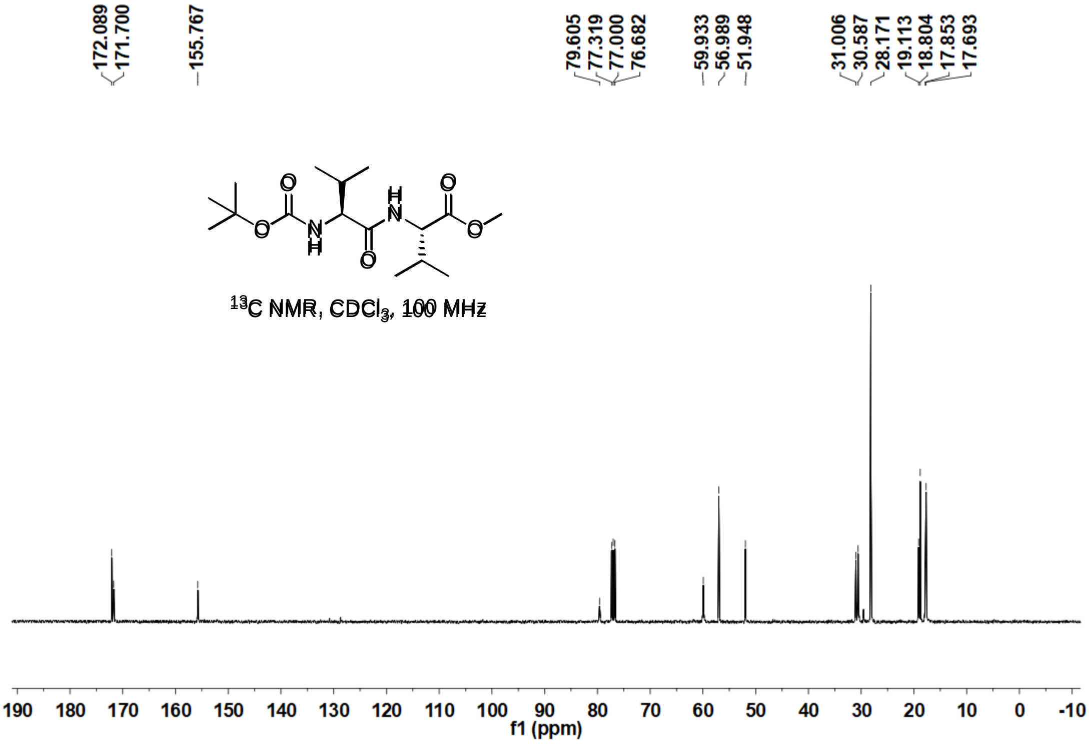

Cbz-L-Val-L-Glu(OEt)-OEt (3-21)

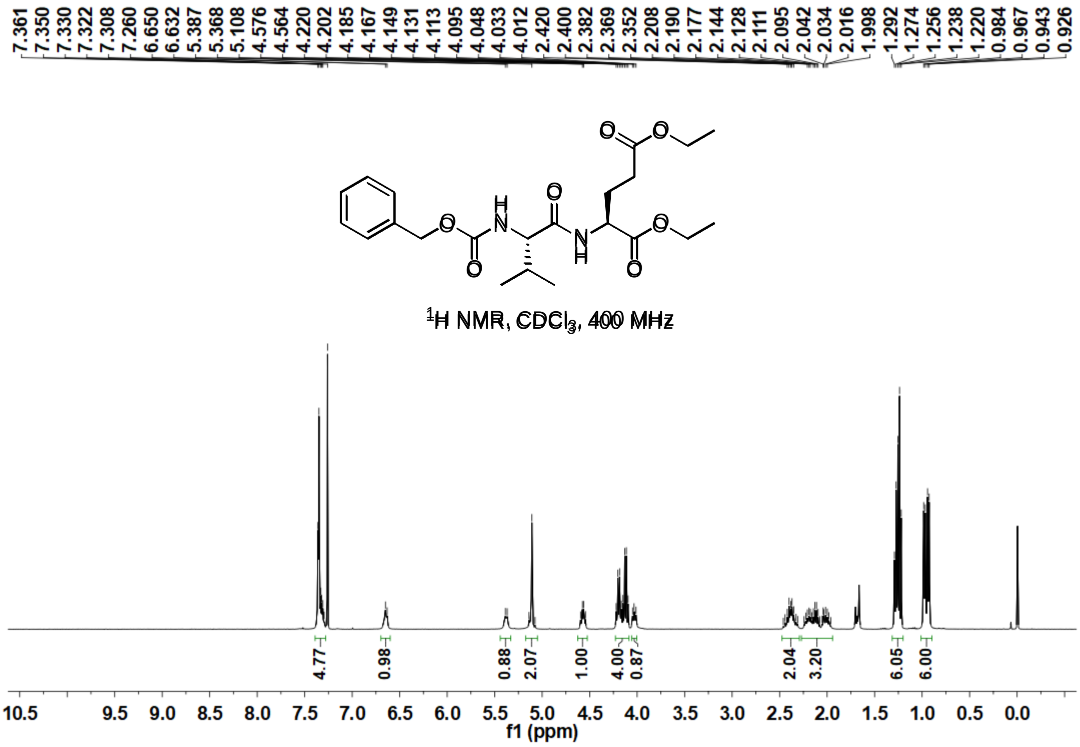

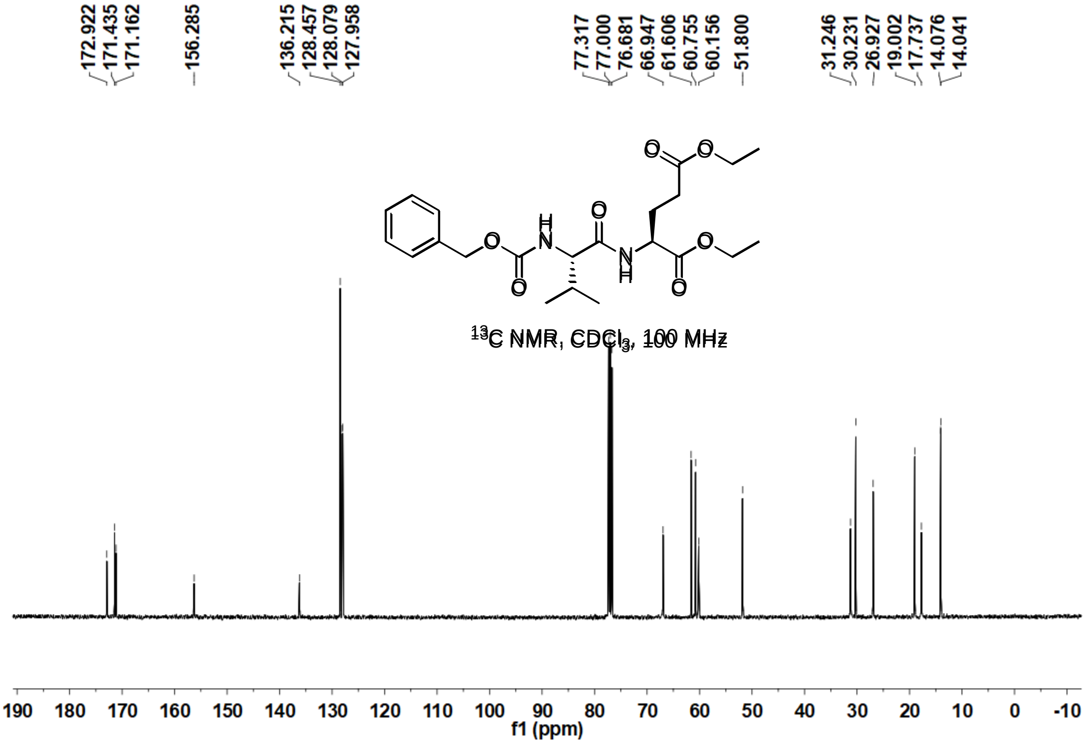

Boc-L-Val-L-Pro-OMe (3-22)

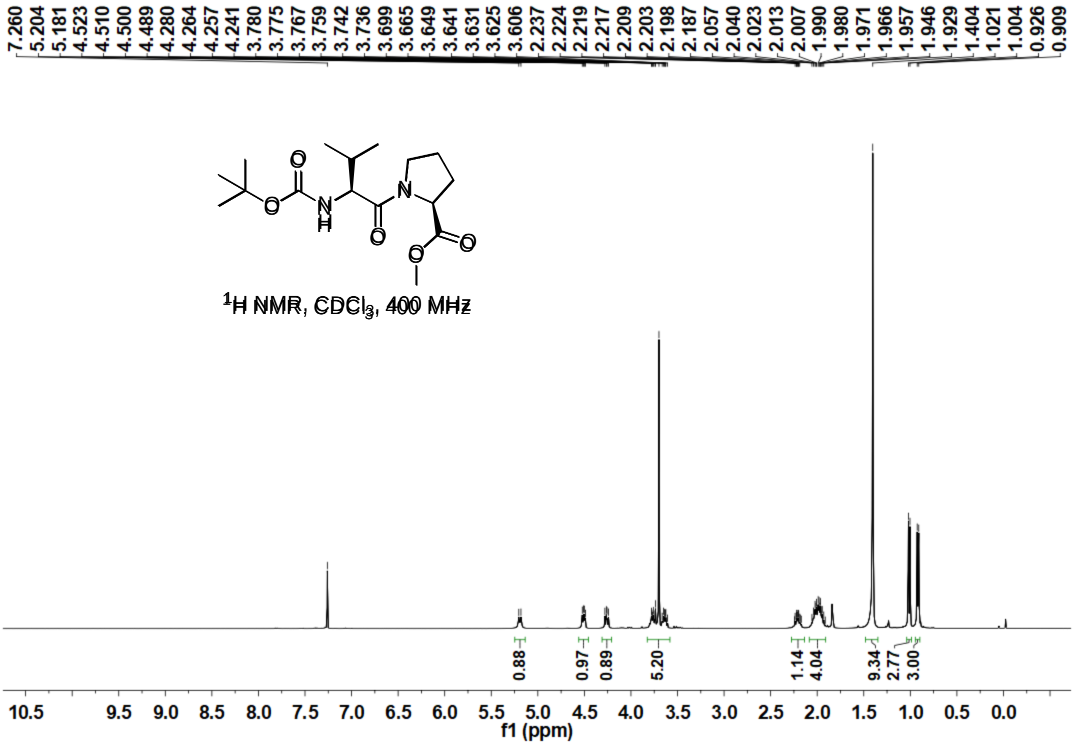

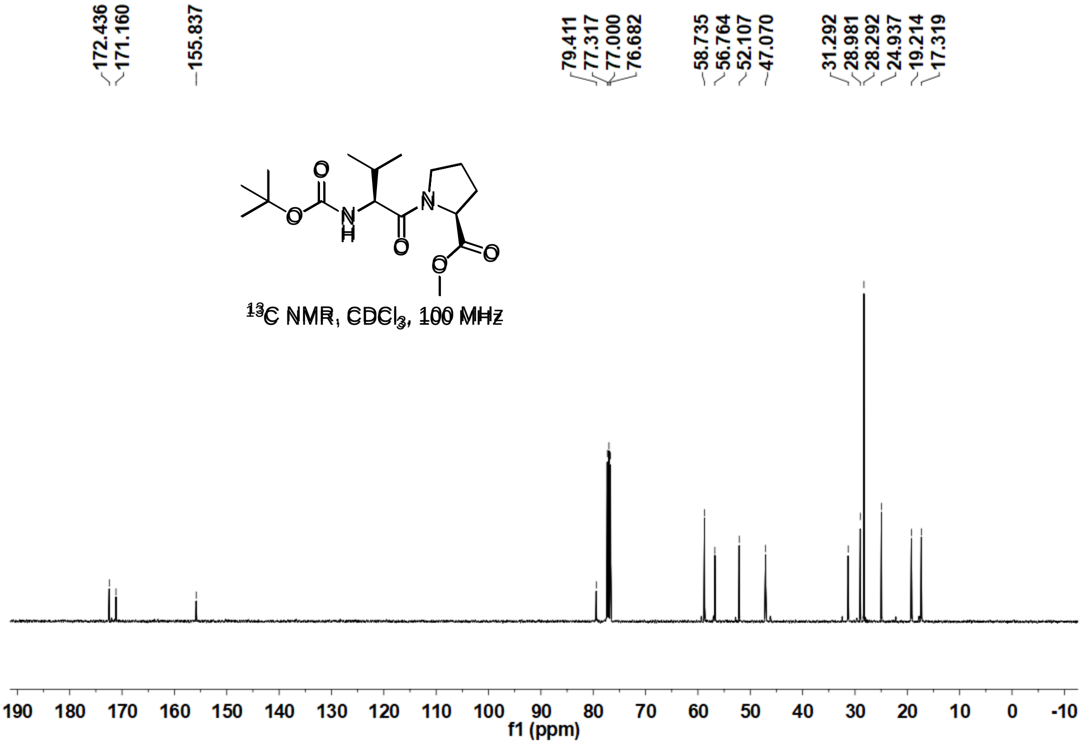

Boc-L- Pro-L-Ala-OMe (3-23)

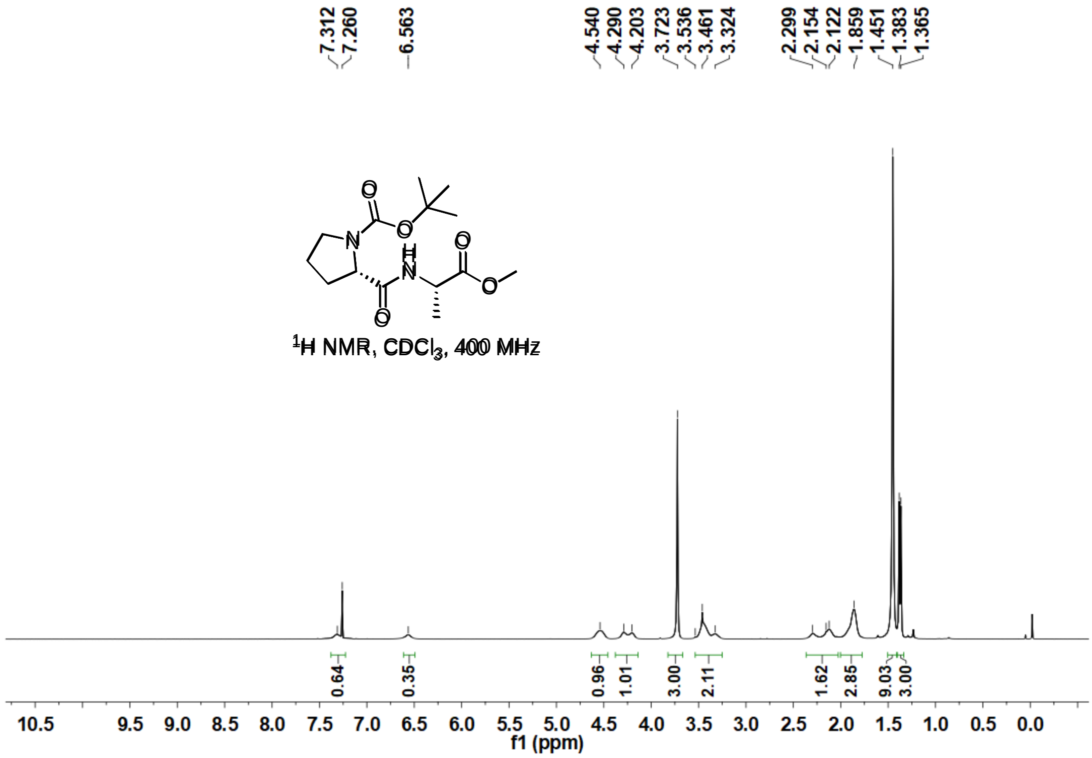

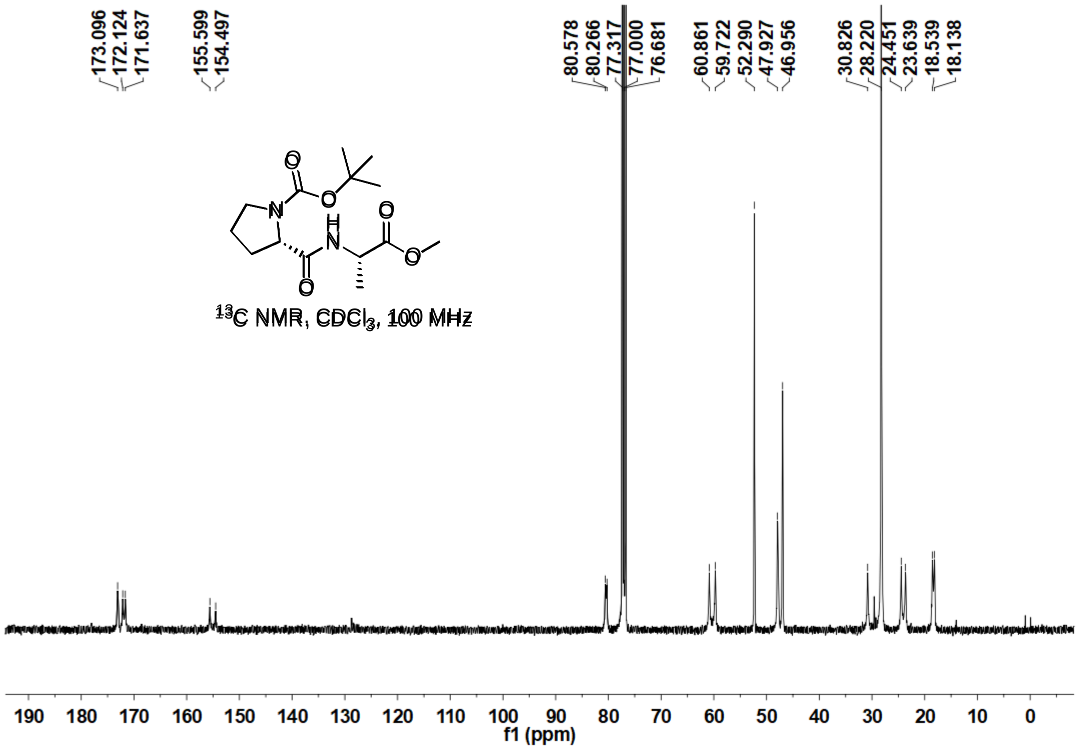

Boc-L- Pro-L-Leu-OMe (3-24)

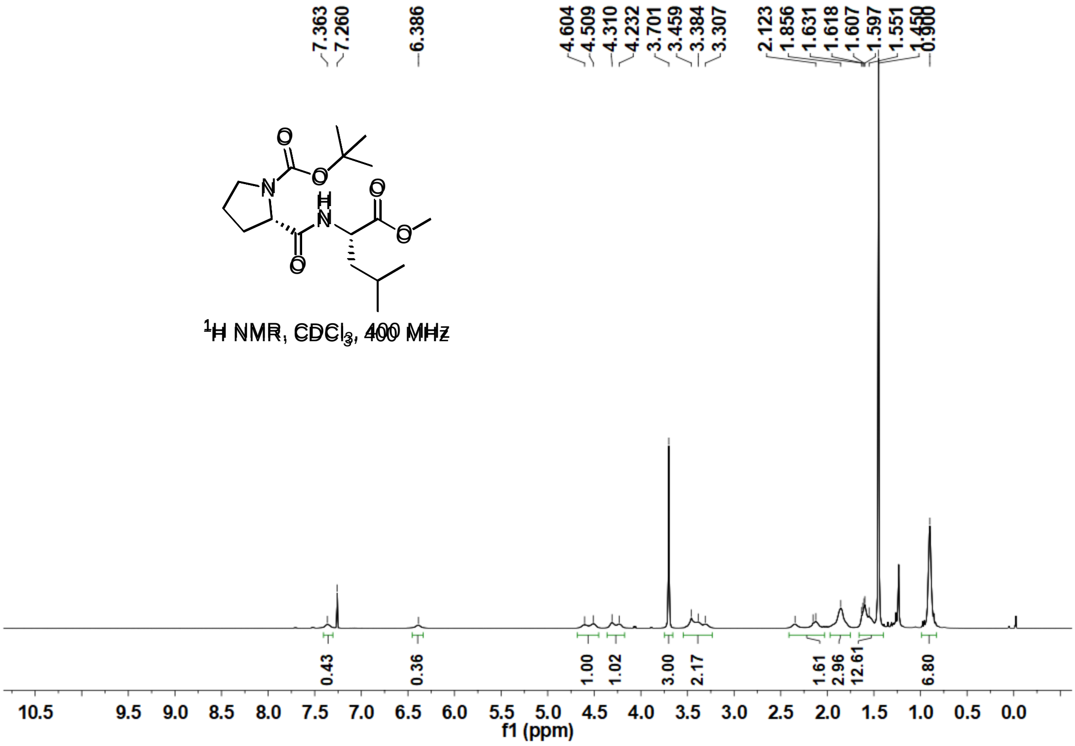

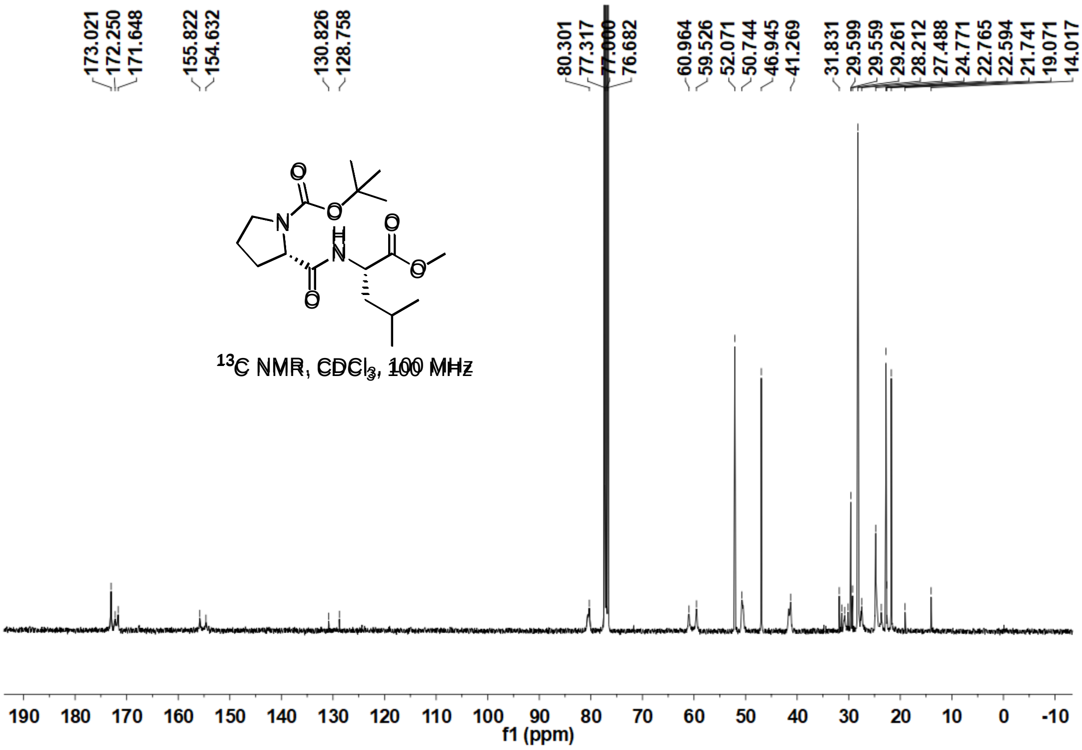

Boc-L- Leu-L-Pro-OMe (3-25)

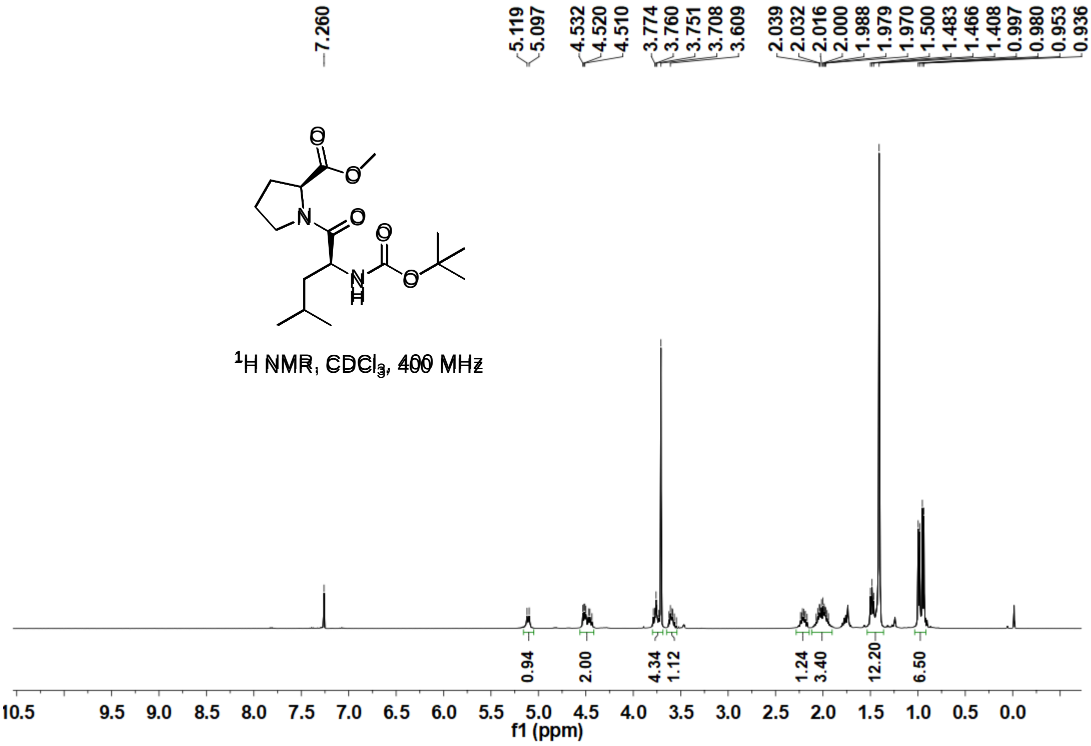

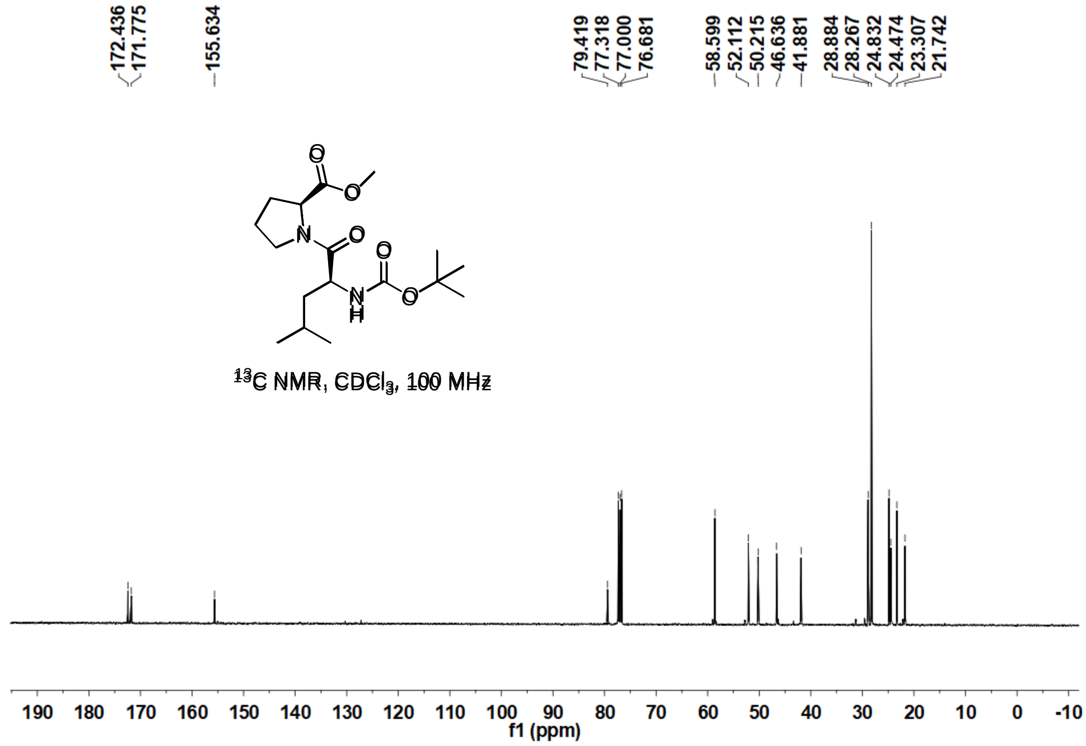

Cbz-Gly-L-Pro-OMe (3-26)

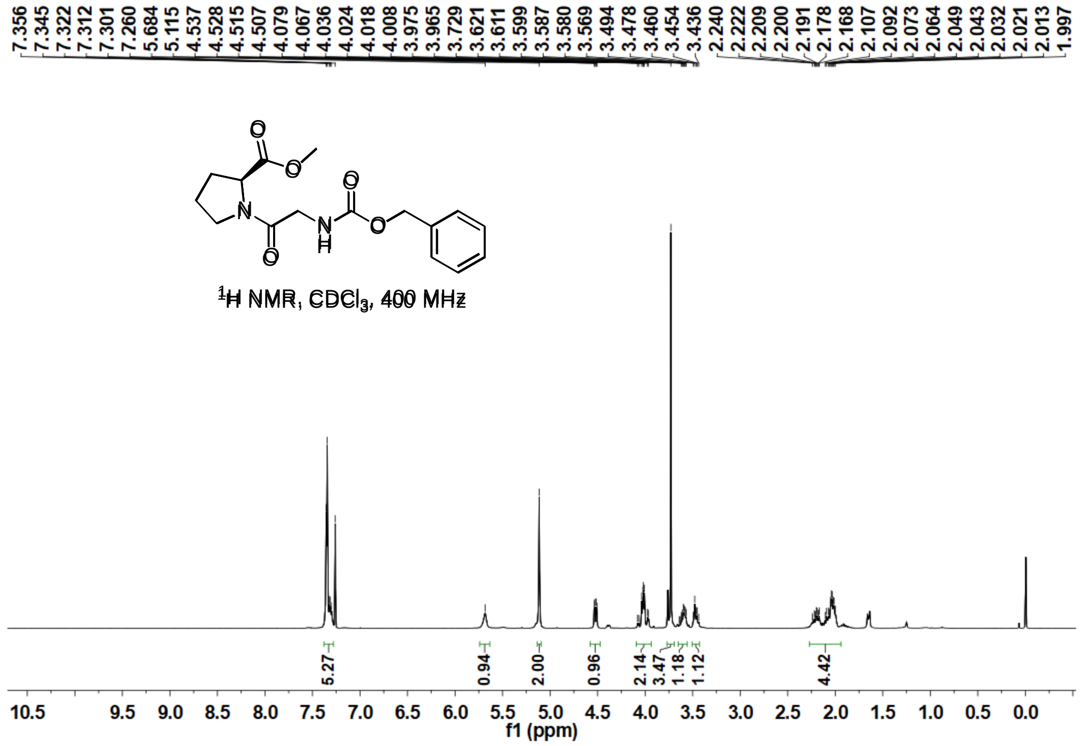

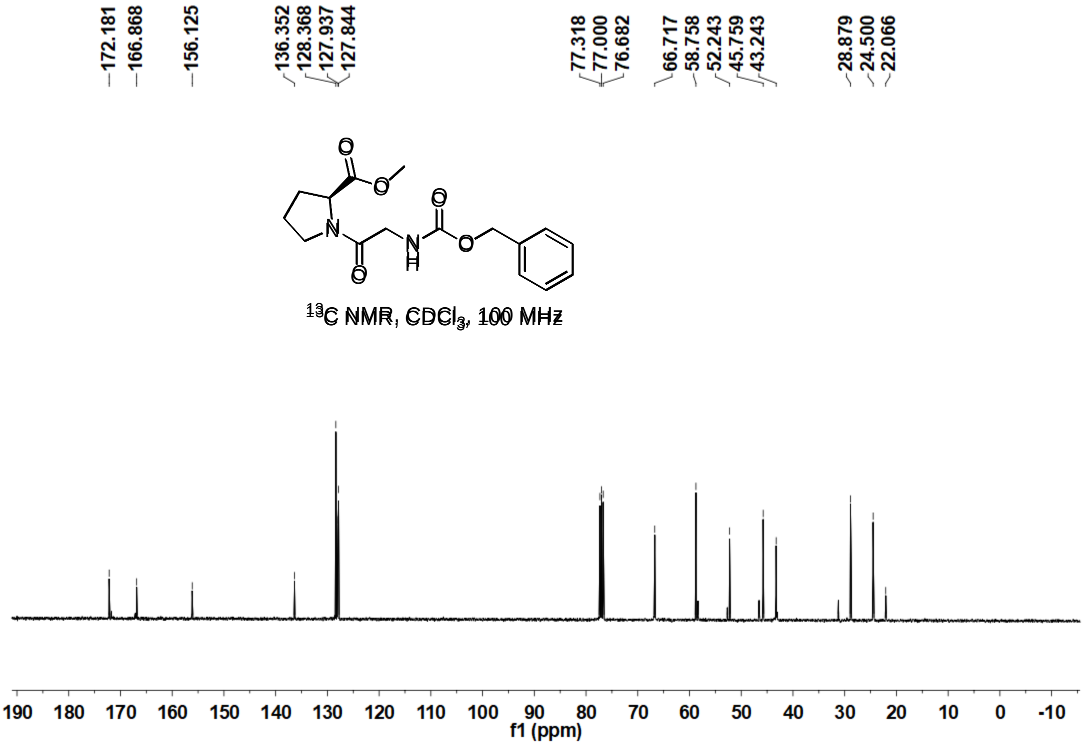

Cbz-L-Ala-Aib-OMe (6-1)

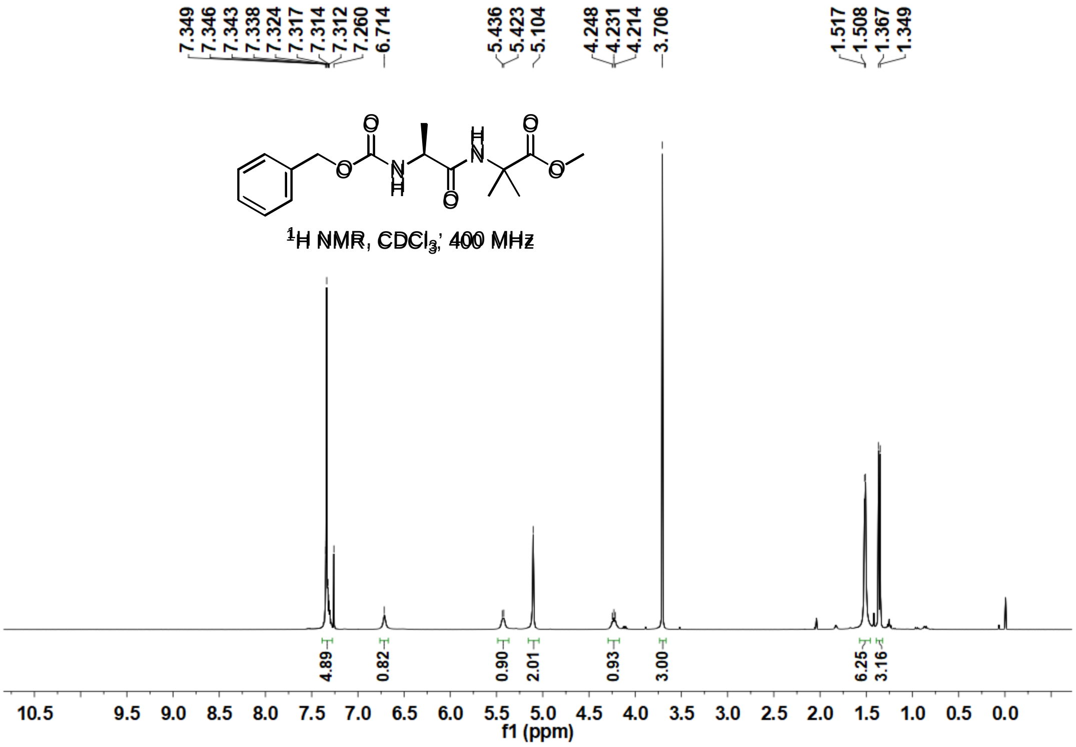
7.349
7.346
7.343
7.338
7.324
7.317
7.314
7.312
7.260
6.714
5.436
5.423
5.104
4.248
4.231
4.214
3.706
1.517
1.508
1.367
1.349
1H NMR, CDCl3, 400 MHz
4.89
0.82
0.90
2.01
0.93
3.00
6.25
3.16
10.5
9.5
9.0
8.5
8.0
7.5
7.0
6.5
6.0
5.5
5.0
4.5
4.0
3.5
3.0
2.5
2.0
1.5
1.0
0.5
0.0
f1 (ppm)

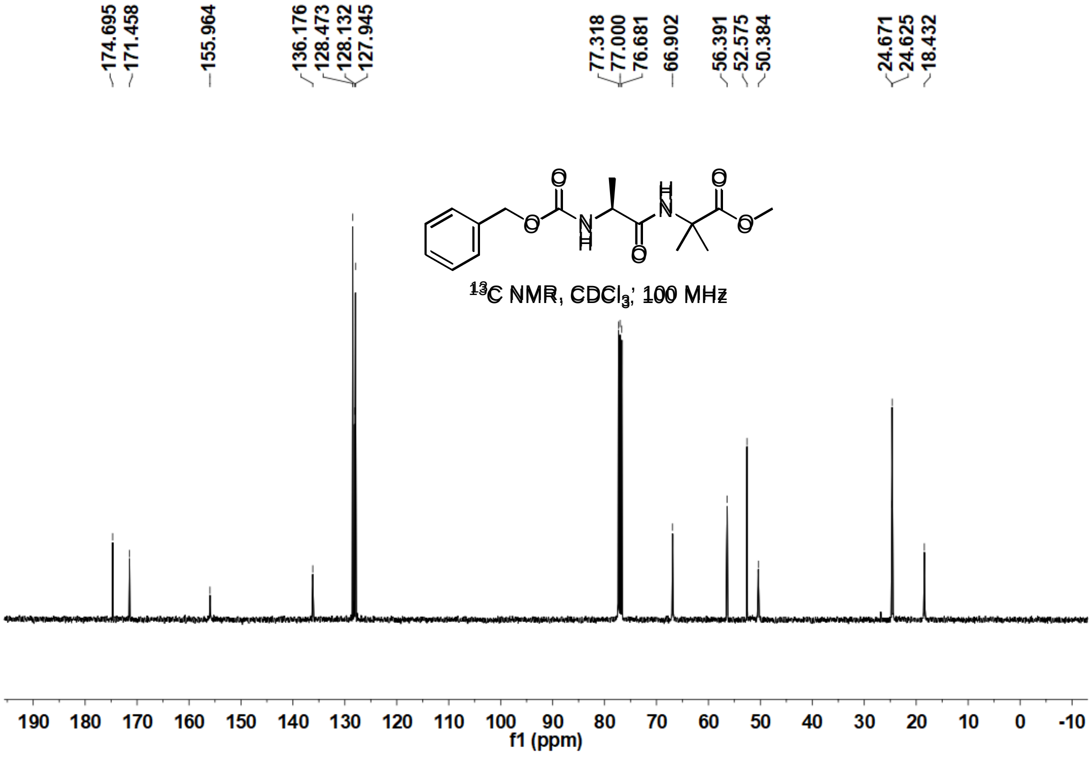
174.695
171.458
155.964
136.176
128.473
128.132
127.945
77.318
77.000
76.681
66.902
56.391
52.575
50.384
24.671
24.625
18.432
13C NMR, CDCl3, 100 MHz
190
180
170
160
150
140
130
120
110
100
90
80
70
60
50
40
30
20
10
0
-10
f1 (ppm)

Boc-Gly-Aib-OMe (6-2)

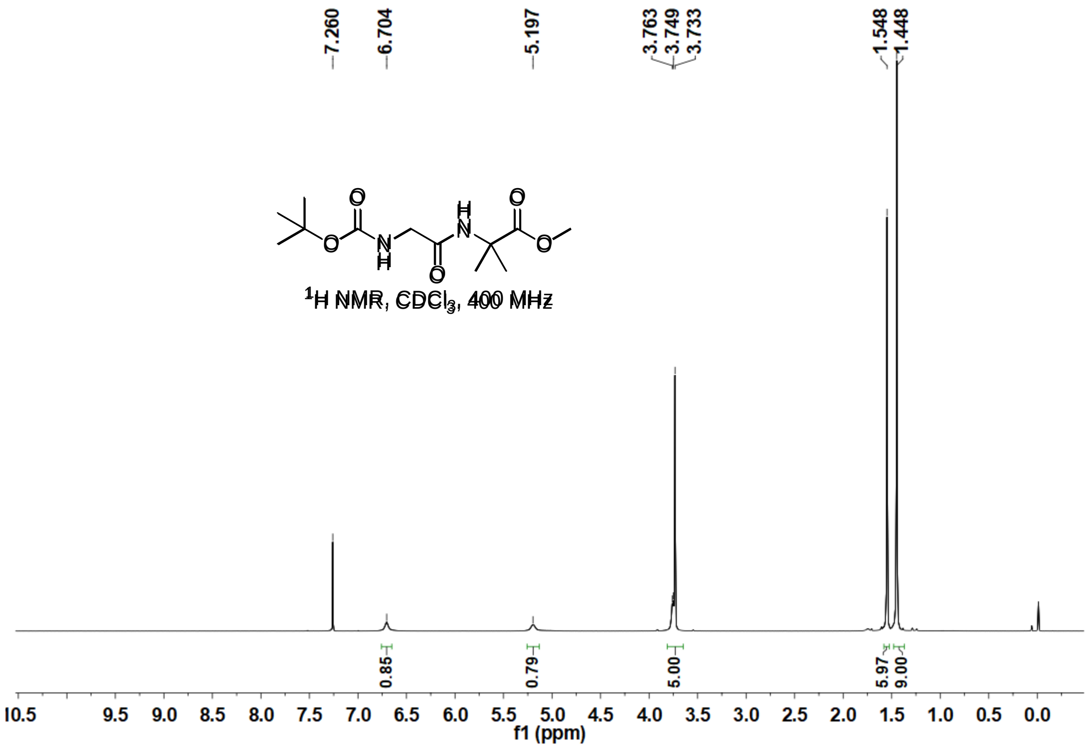

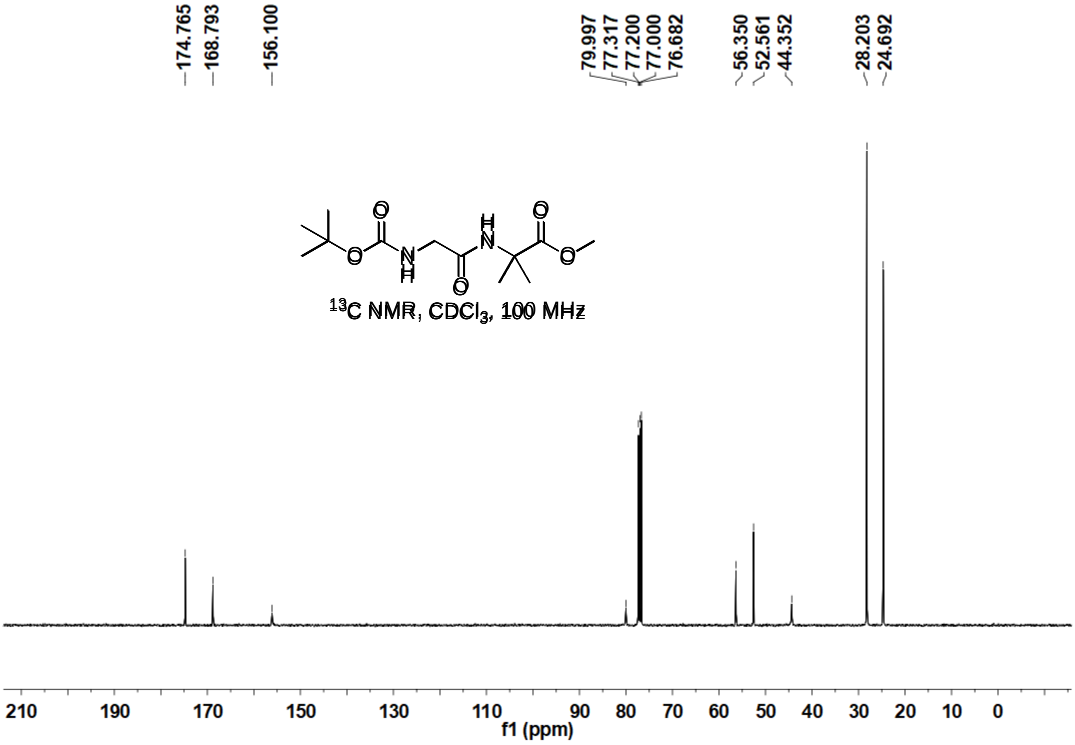

Boc-L-Leu-Aib-OMe (6-3)

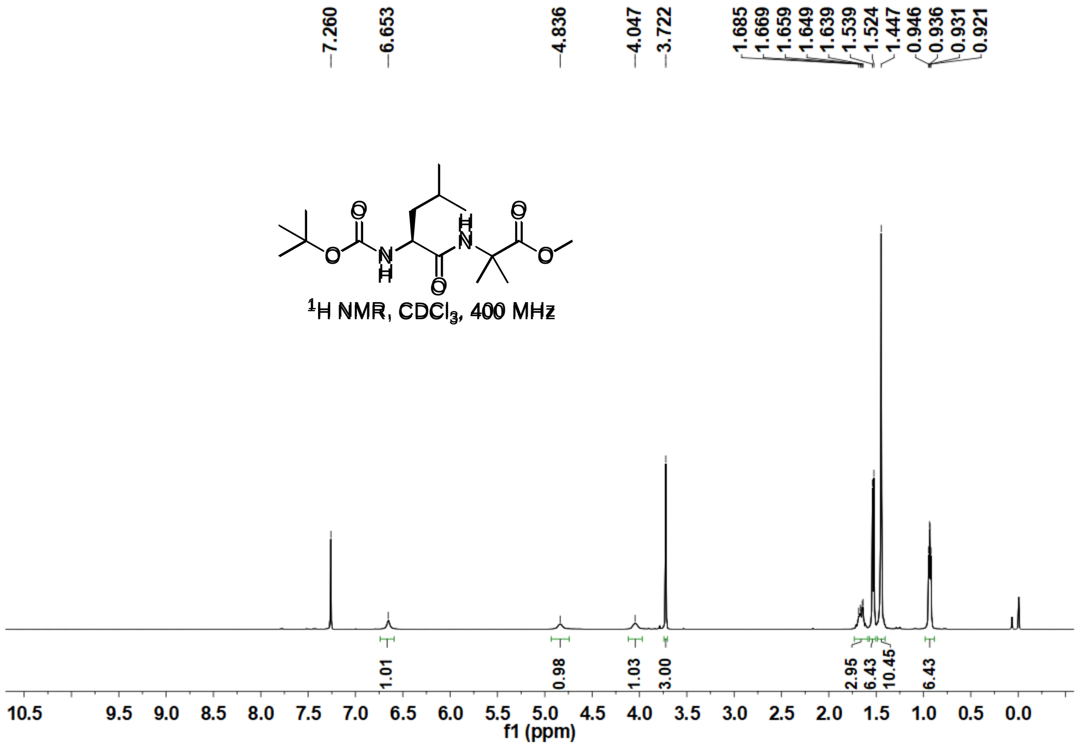

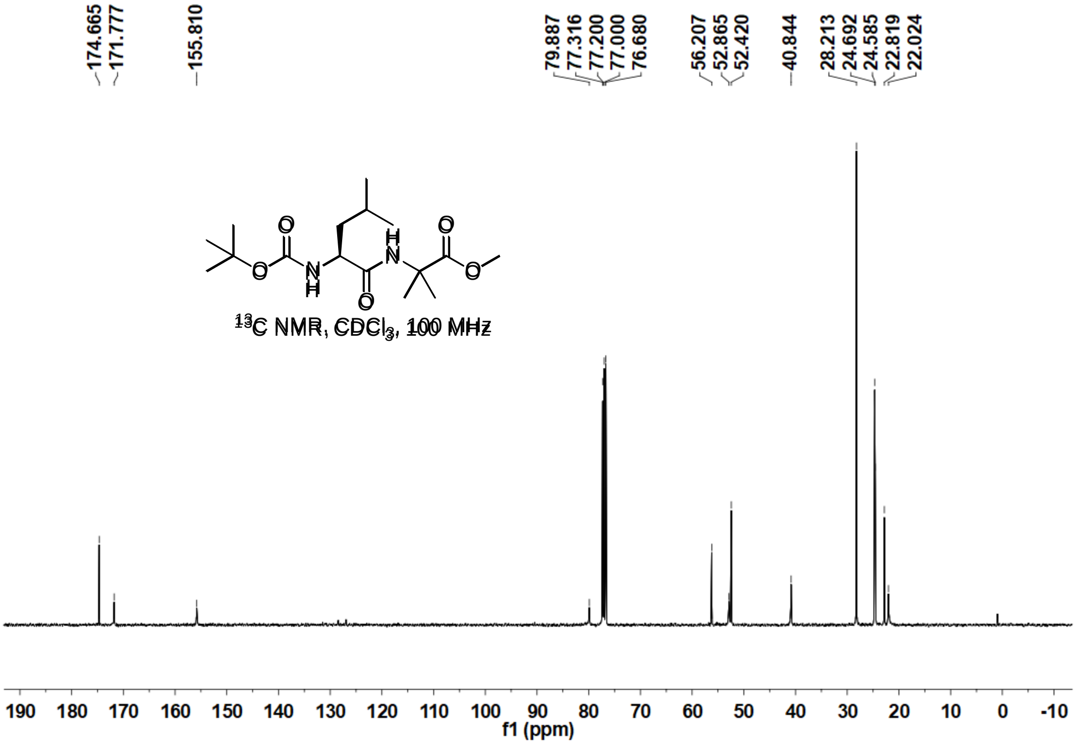

Boc-L-Tyr-Aib-OMe (6-4)

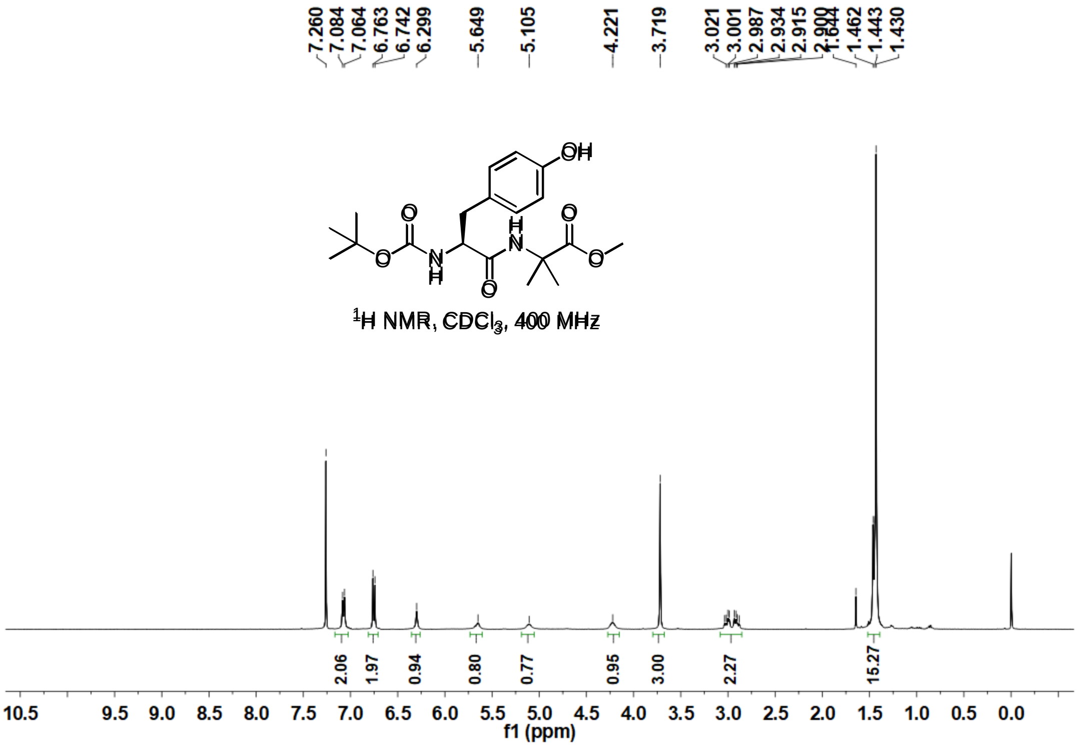

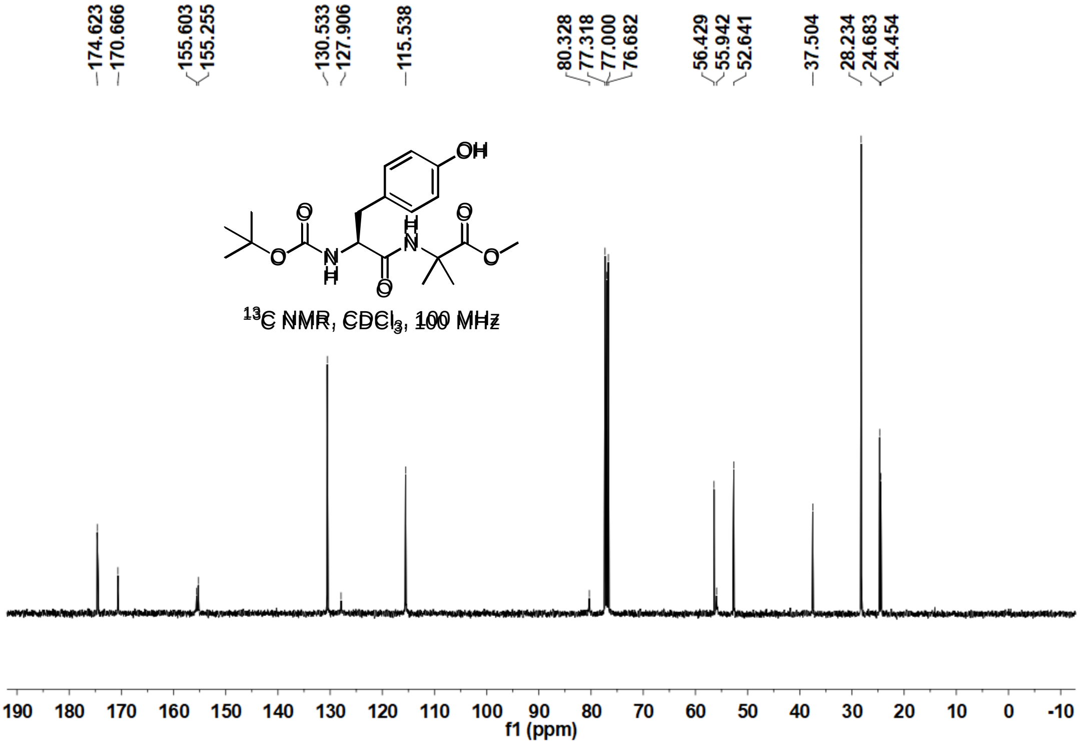

Boc-L-Pro-Aib-OMe (6-5)

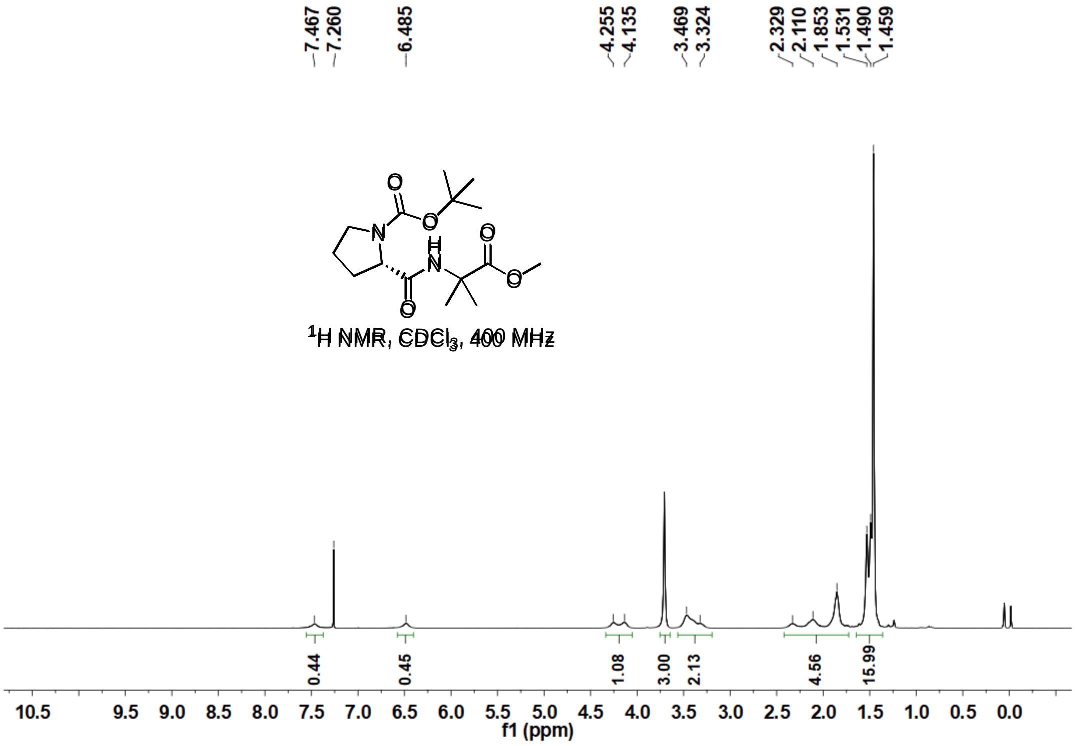

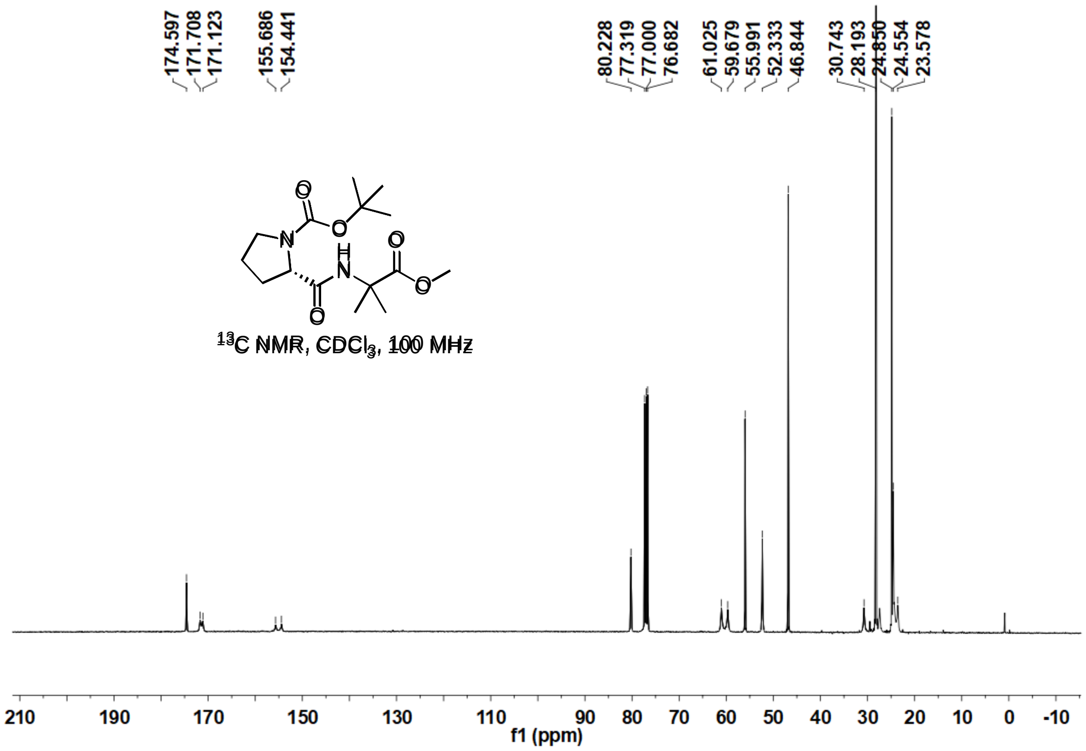

Cbz-L-Trp-Aib-OMe (6-6)

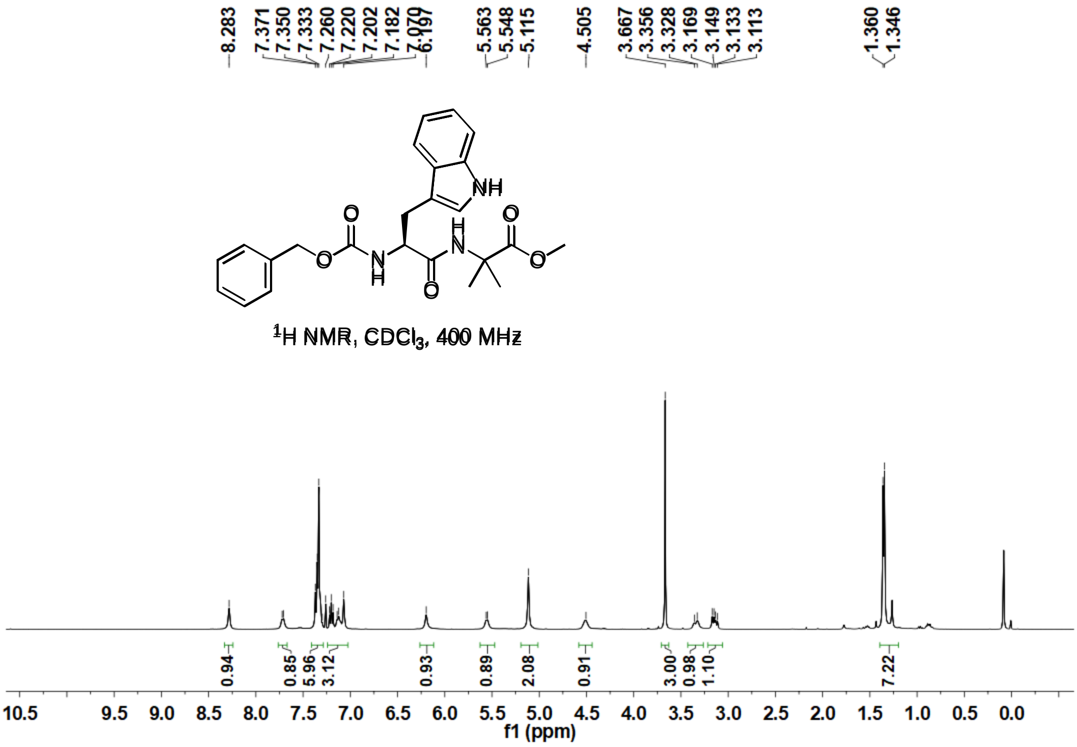

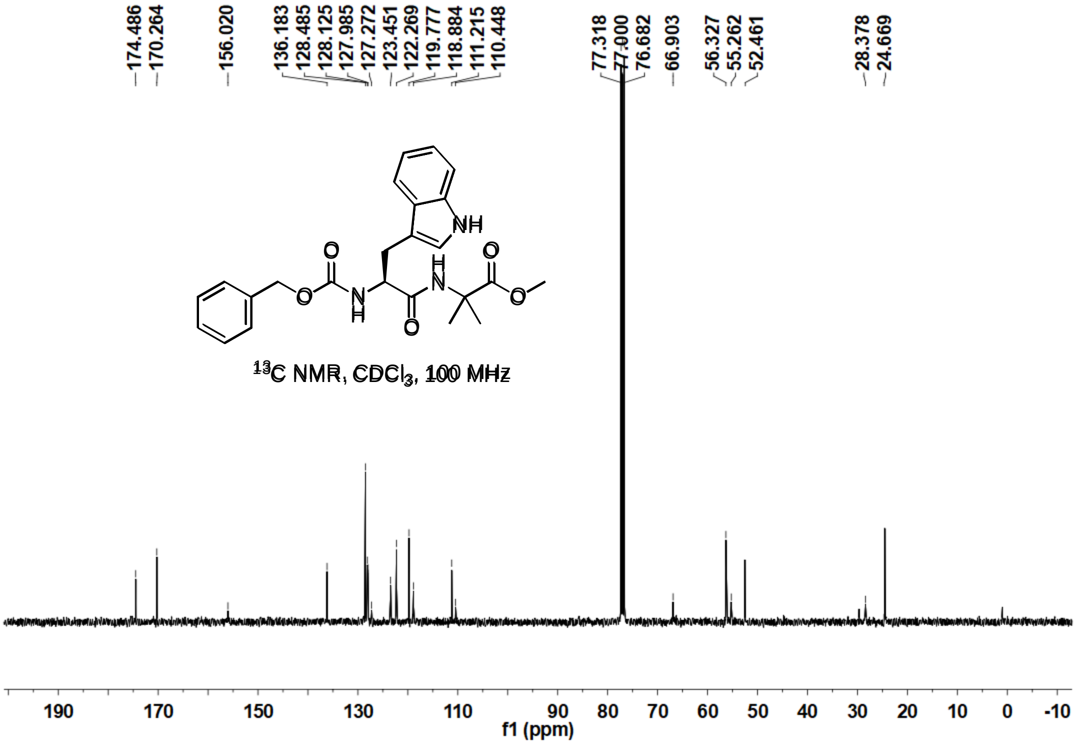

Cbz-L-Phe-Aib-OMe (6-7)

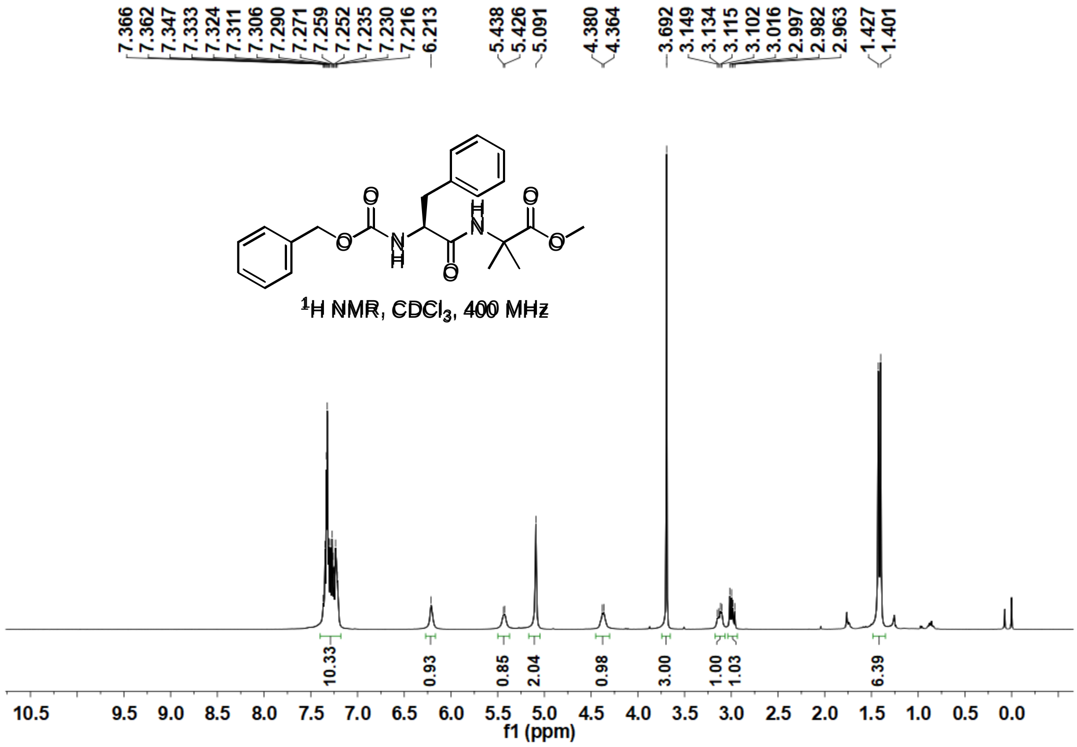

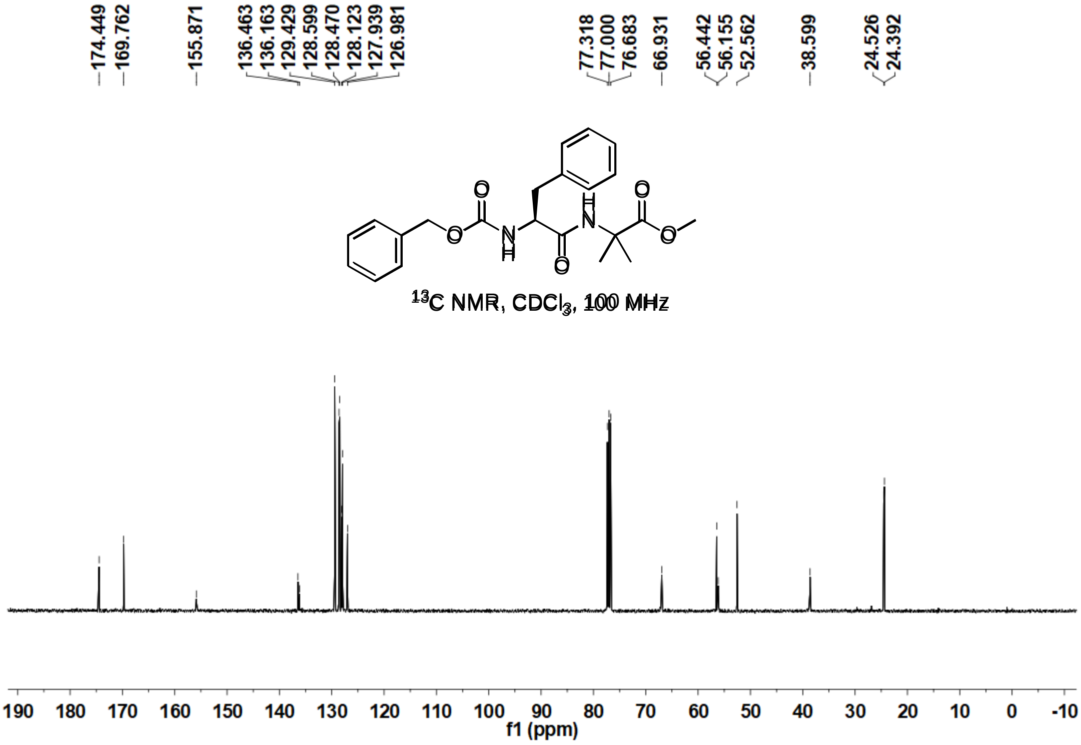

Boc-L-Ala-Aib-OMe (6-8)

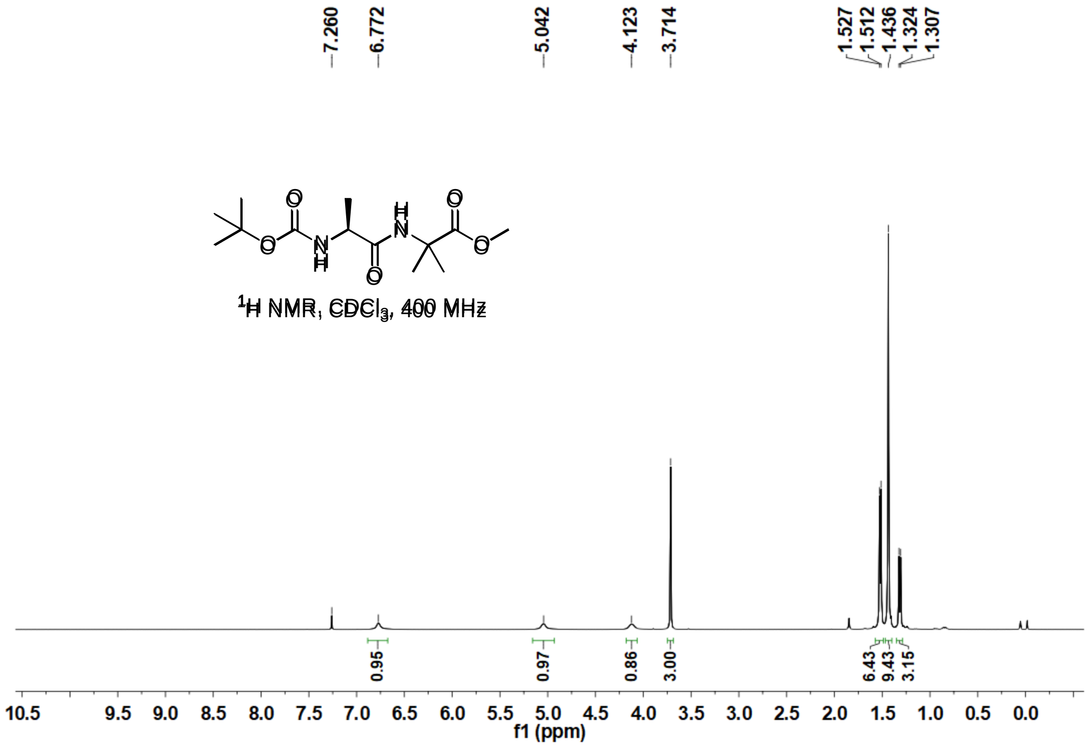

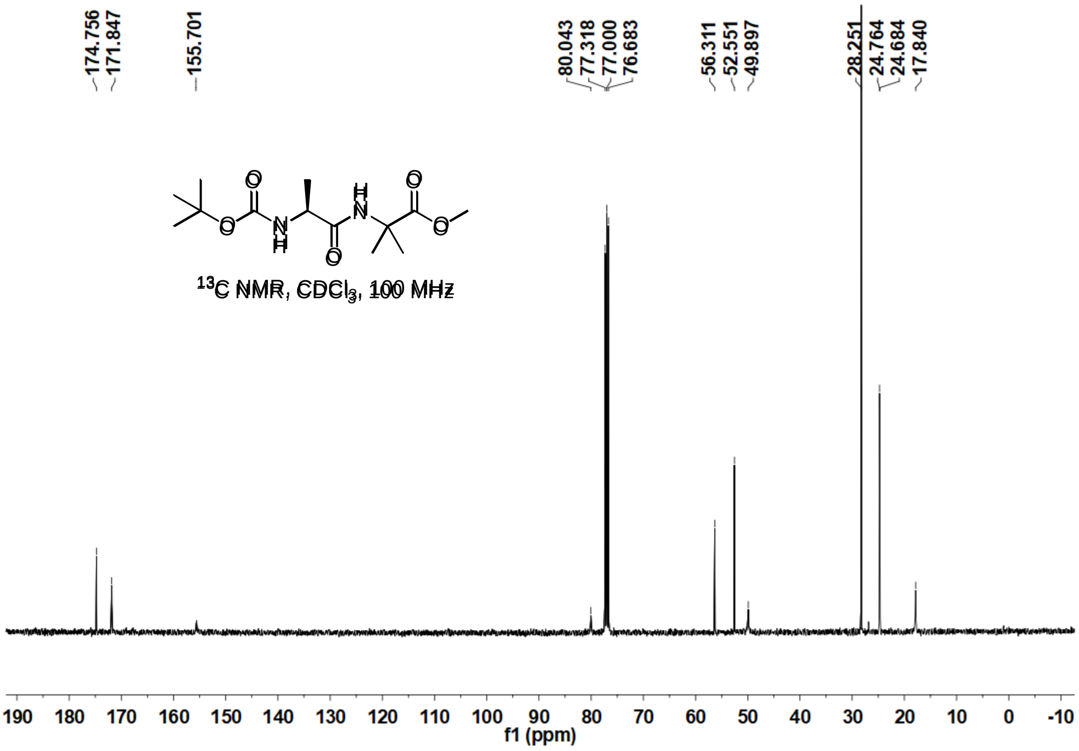

Cbz-L-Met-Aib-OMe (6-9)

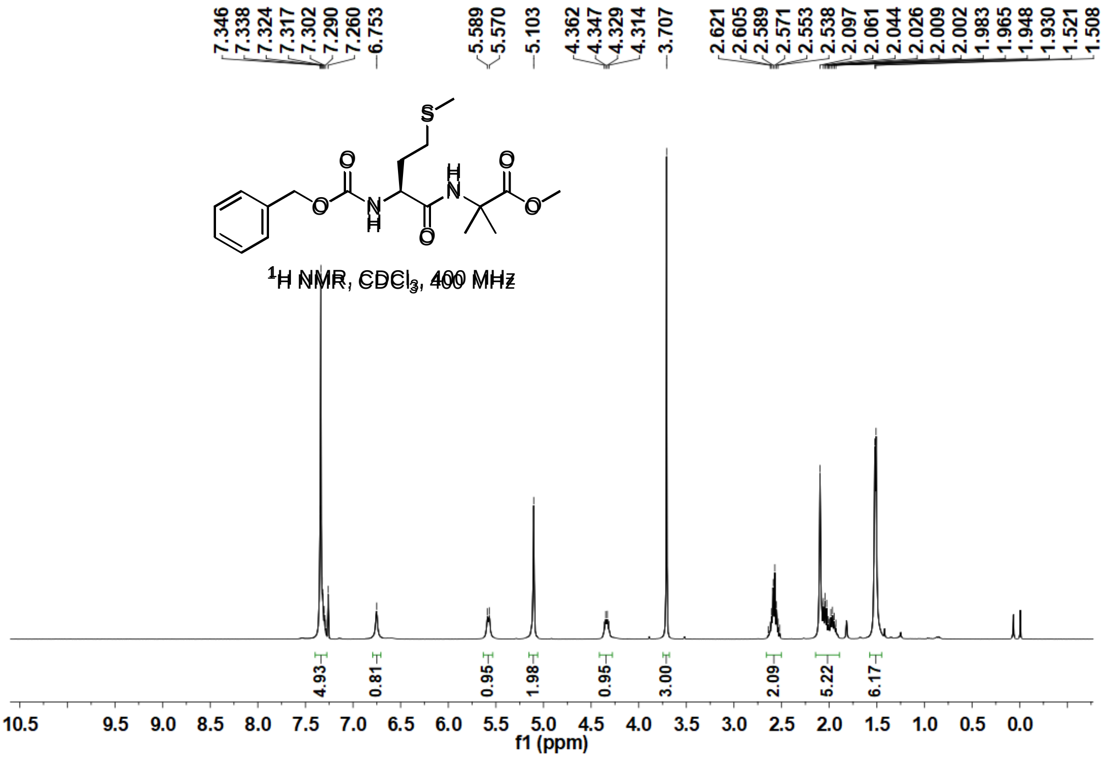

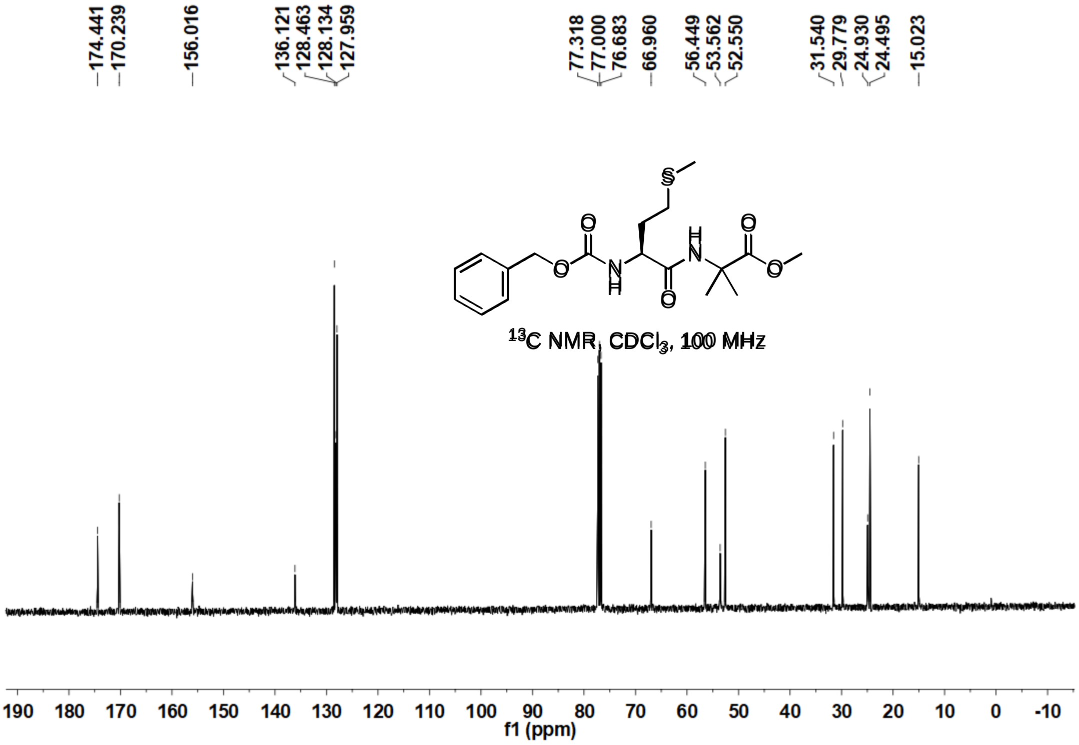

Cbz-L-Ser(*t*Bu)-Aib-OMe (6-10)

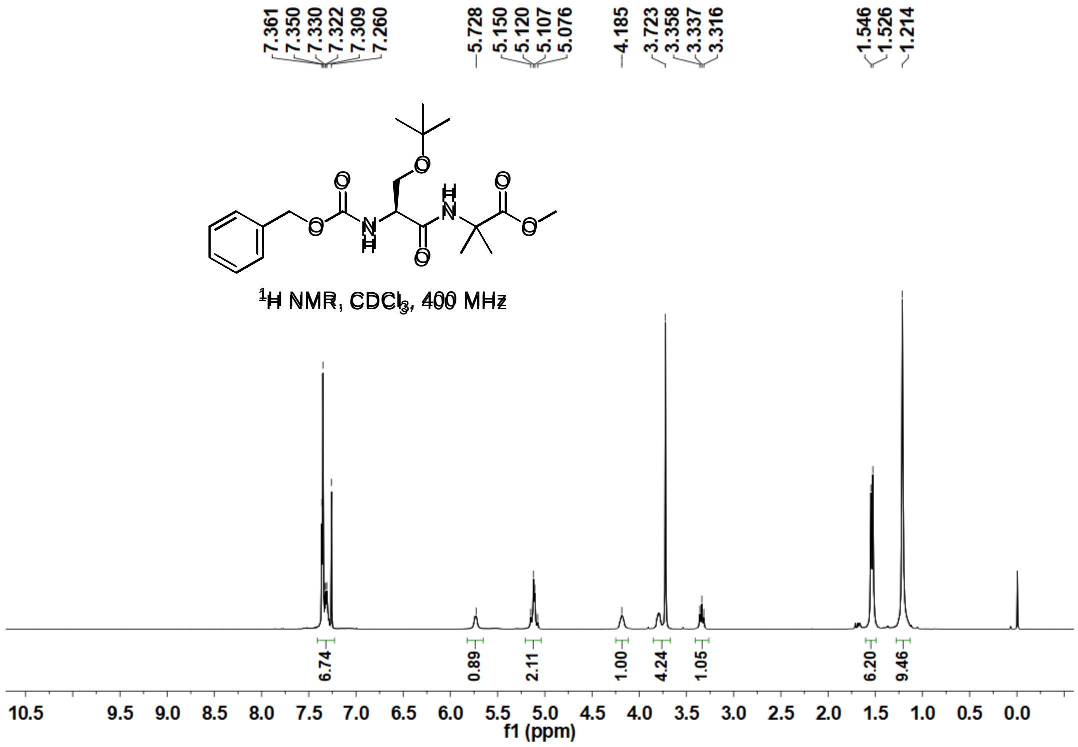

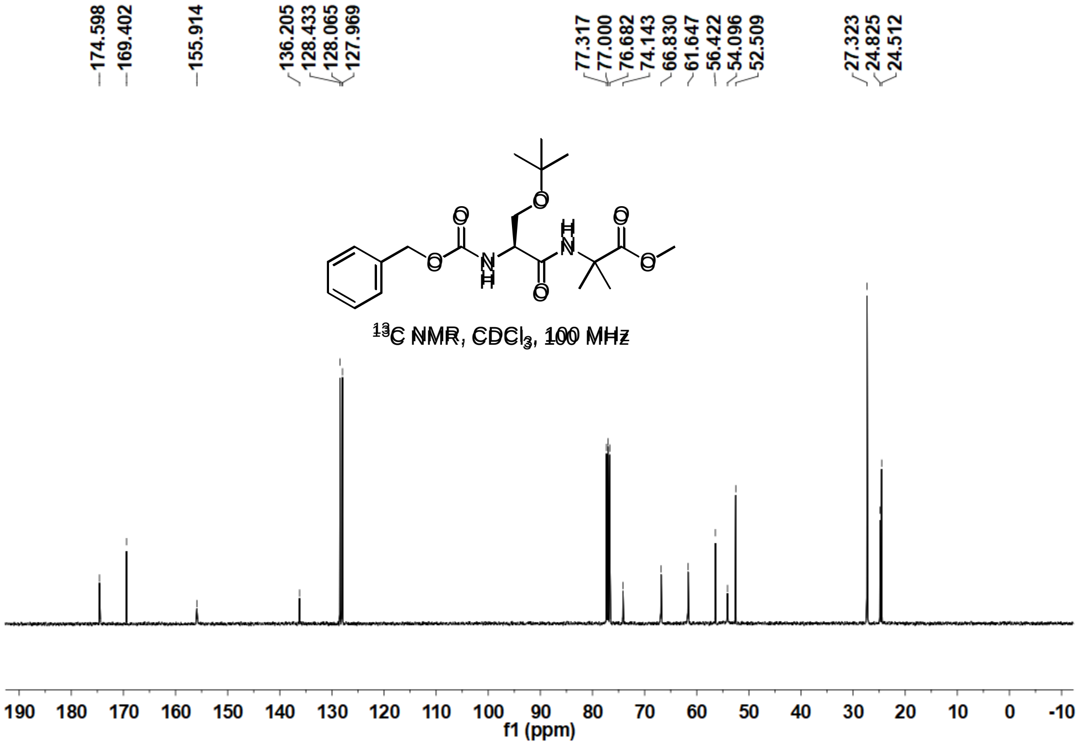

Cbz-L-Val-Aib-OMe (6-11)

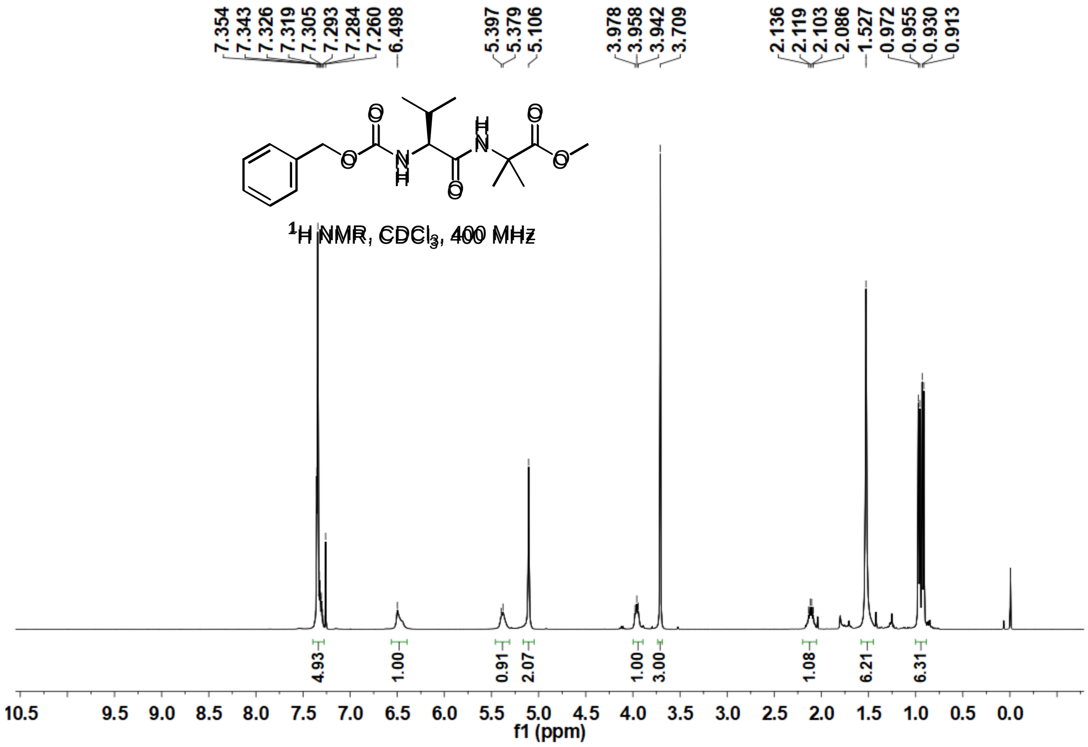

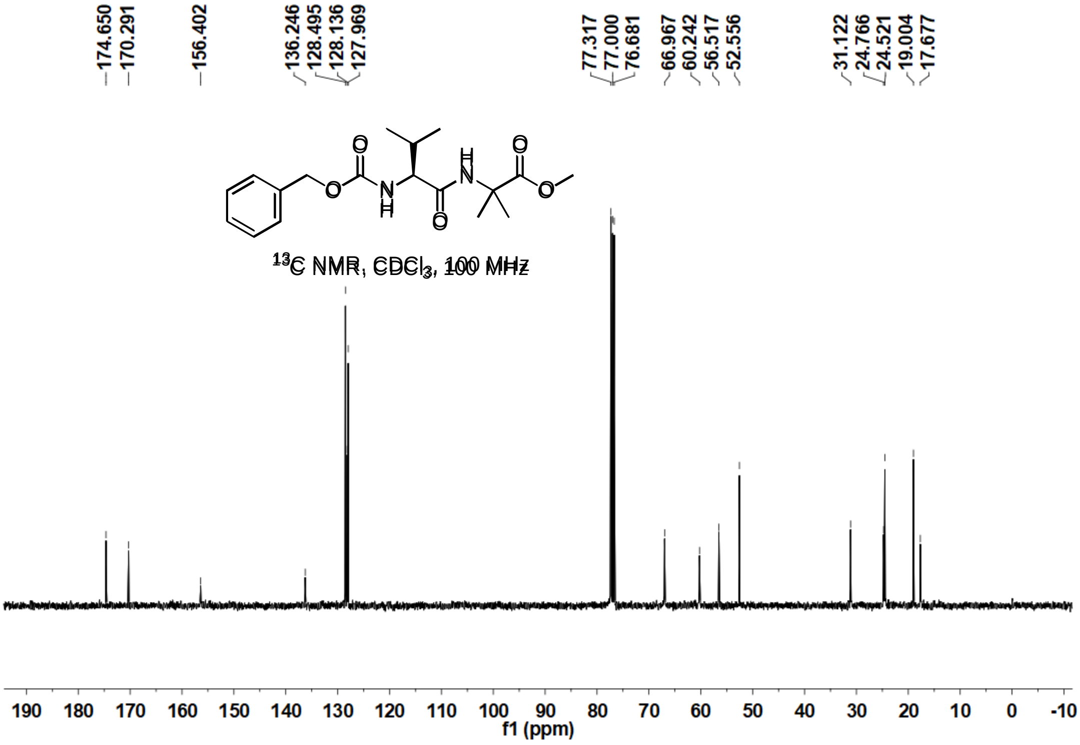

Cbz-L-NMePhe-Aib-OMe (6-12)

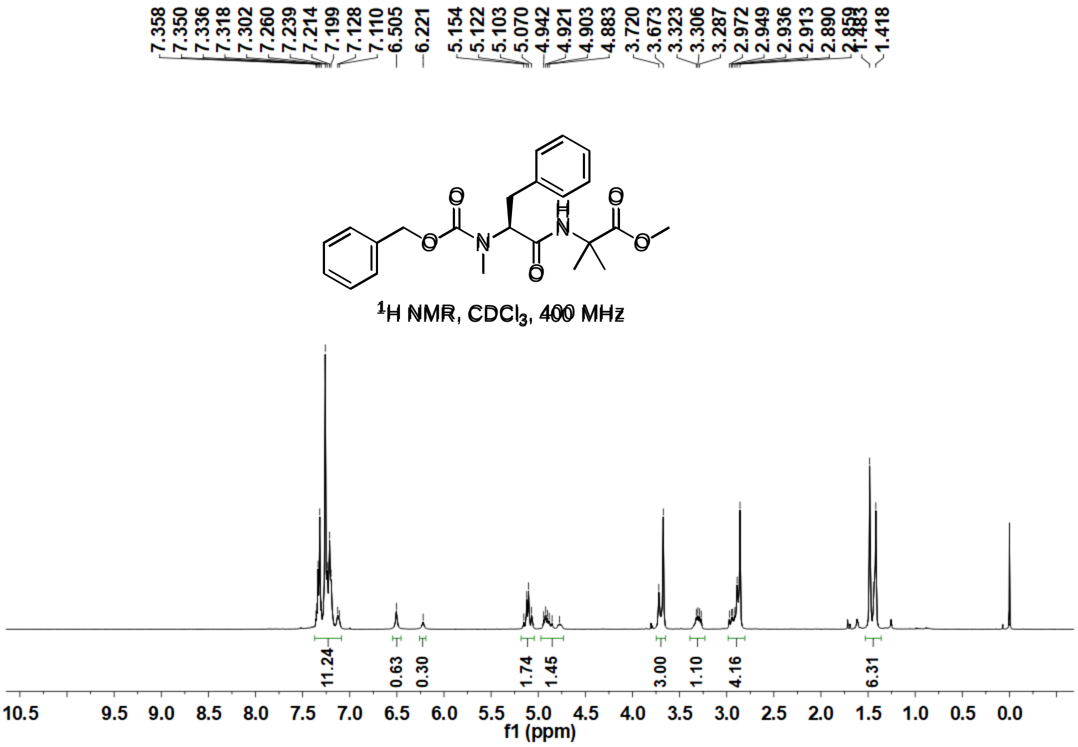

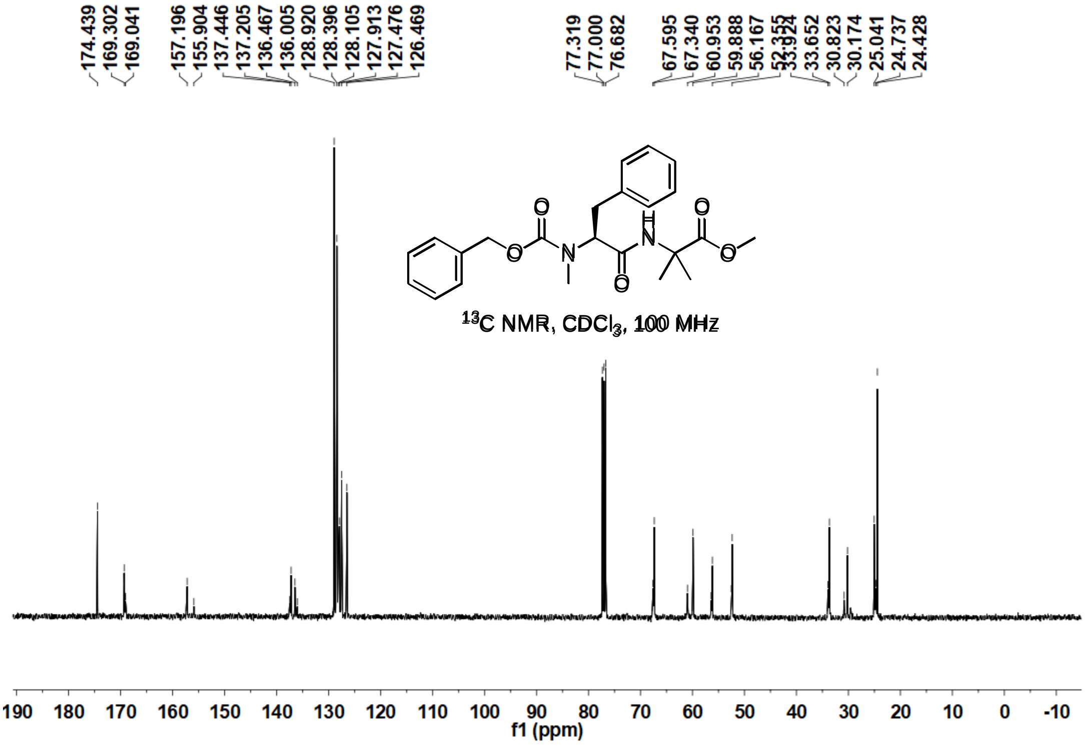

Cbz-L-NMePhe-L-Val-OMe (6-13)

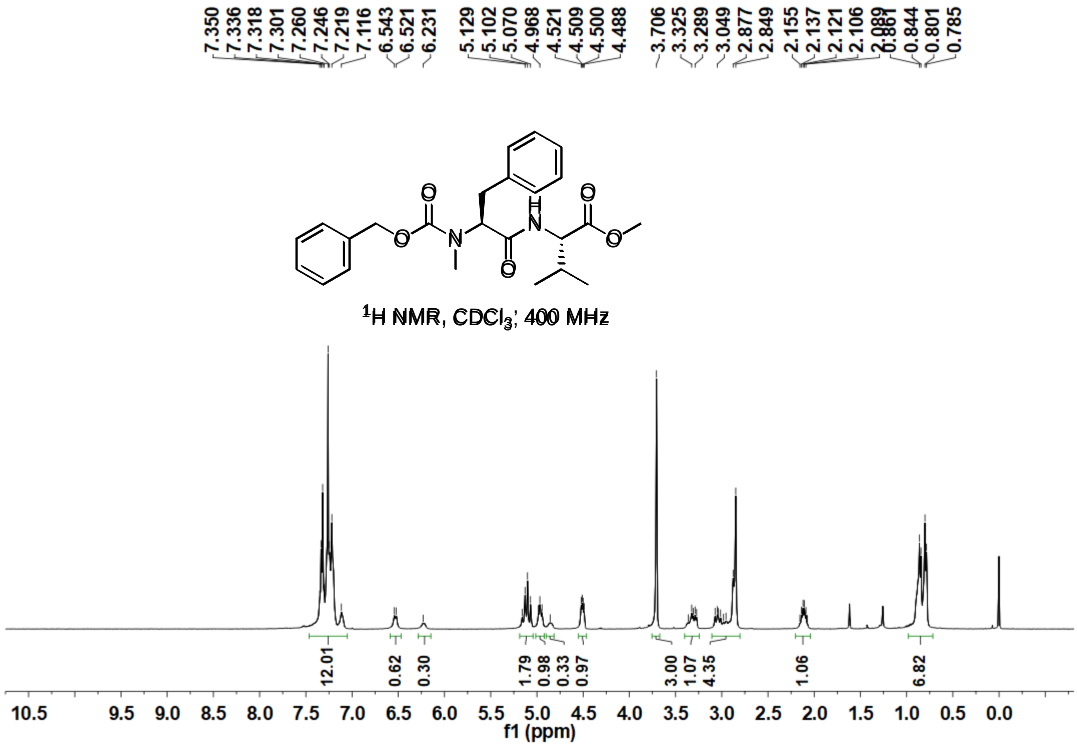

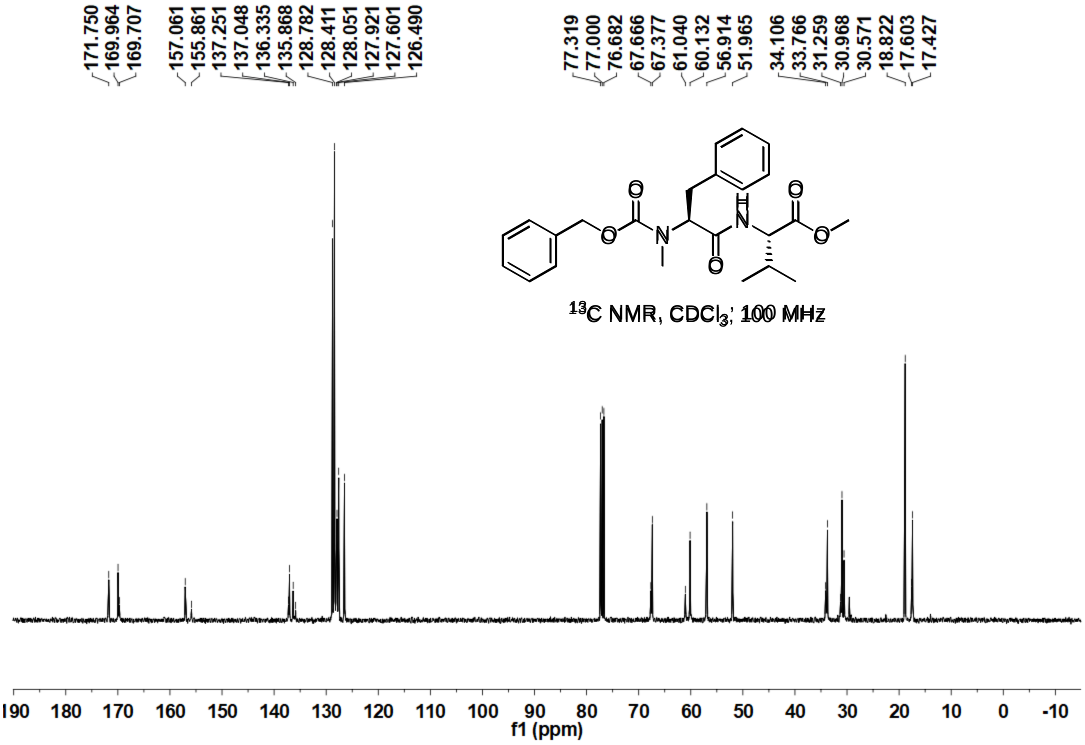

Cbz-L-NMePhe-Gly-OMe (6-14)

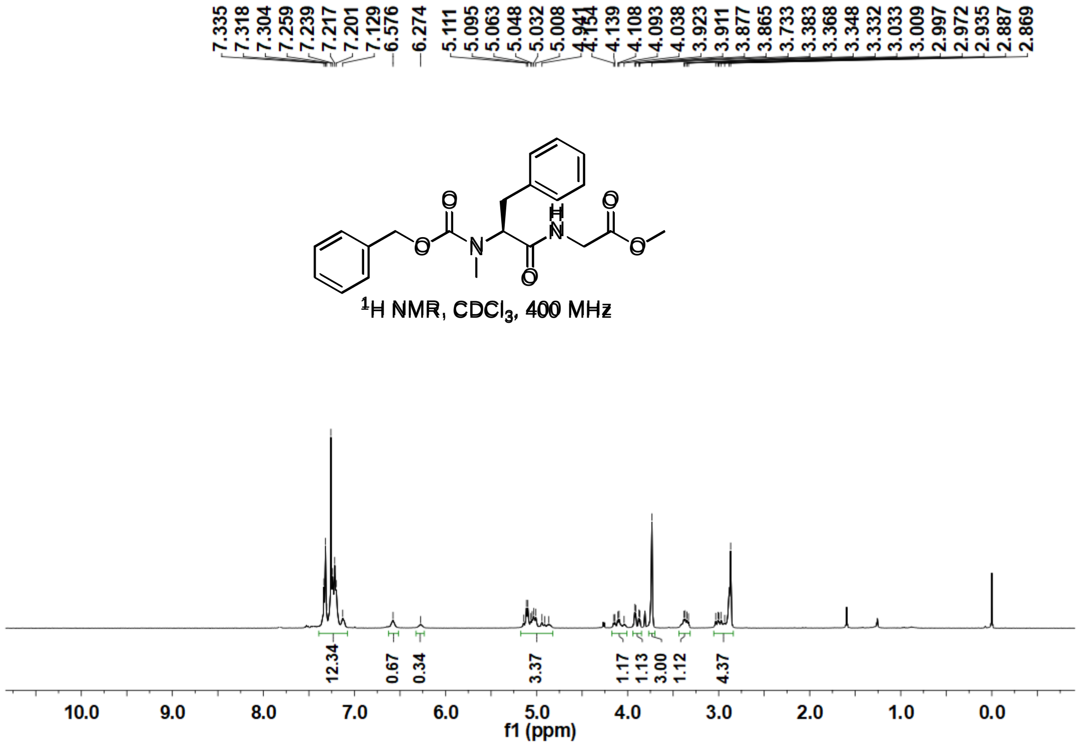

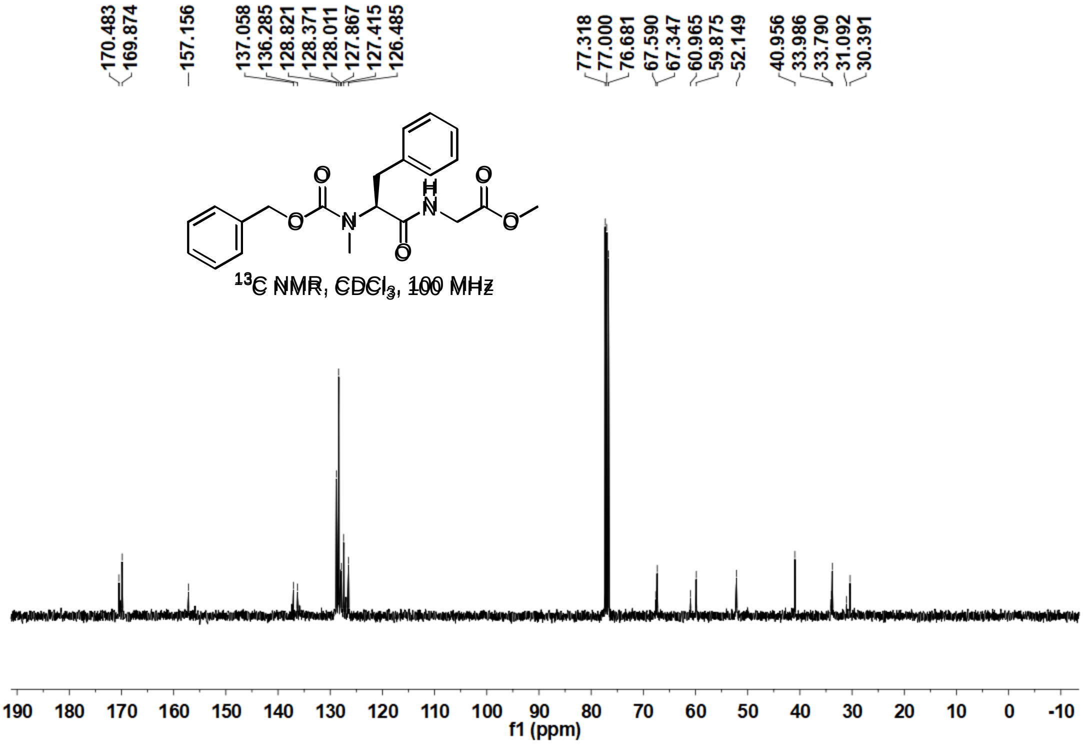

Cbz-L-NMePhe-L-Ala-OMe (6-15)

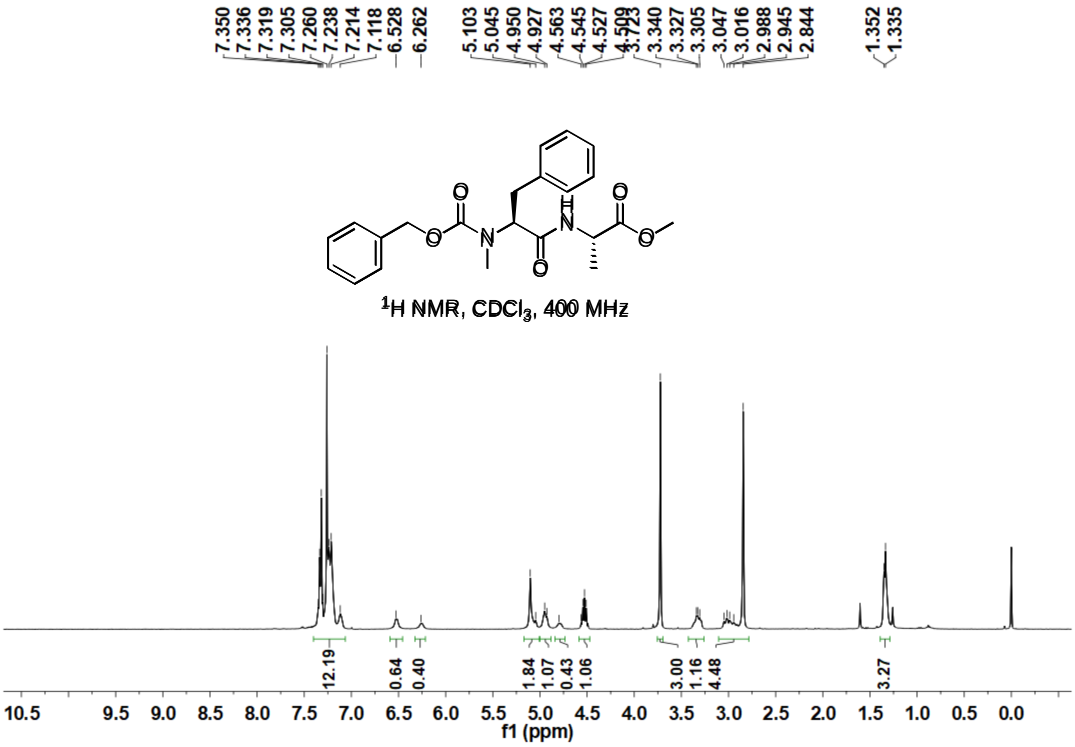

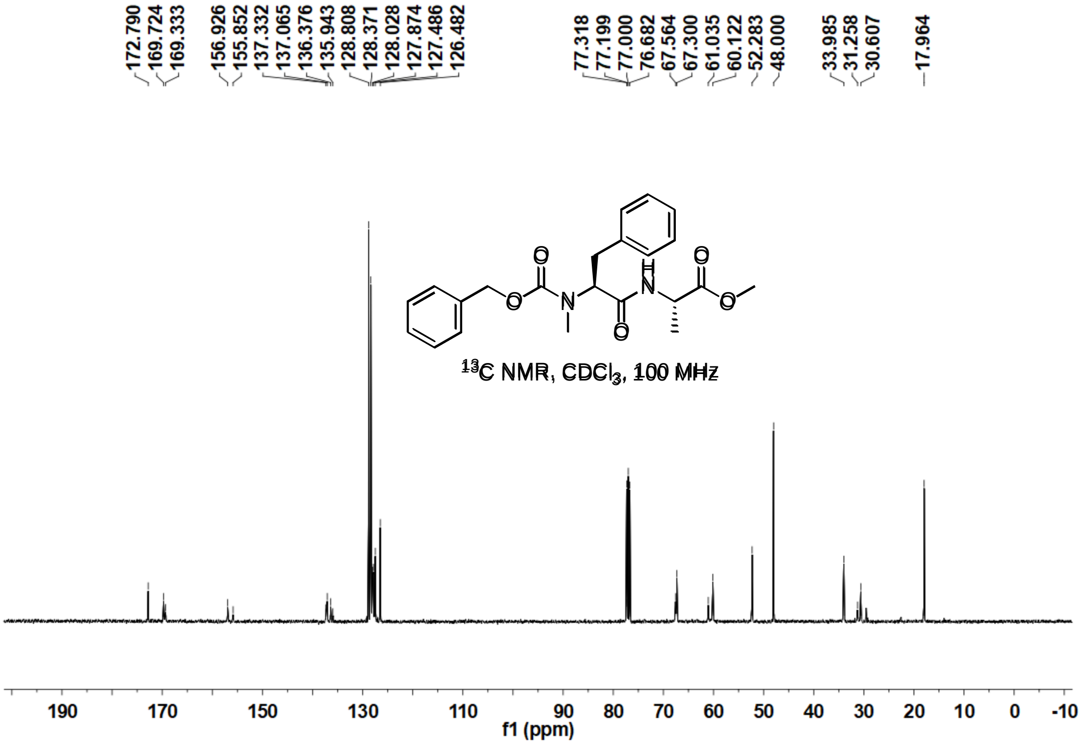

Cbz-L-NMePhe-L-Ile-OMe (6-16)

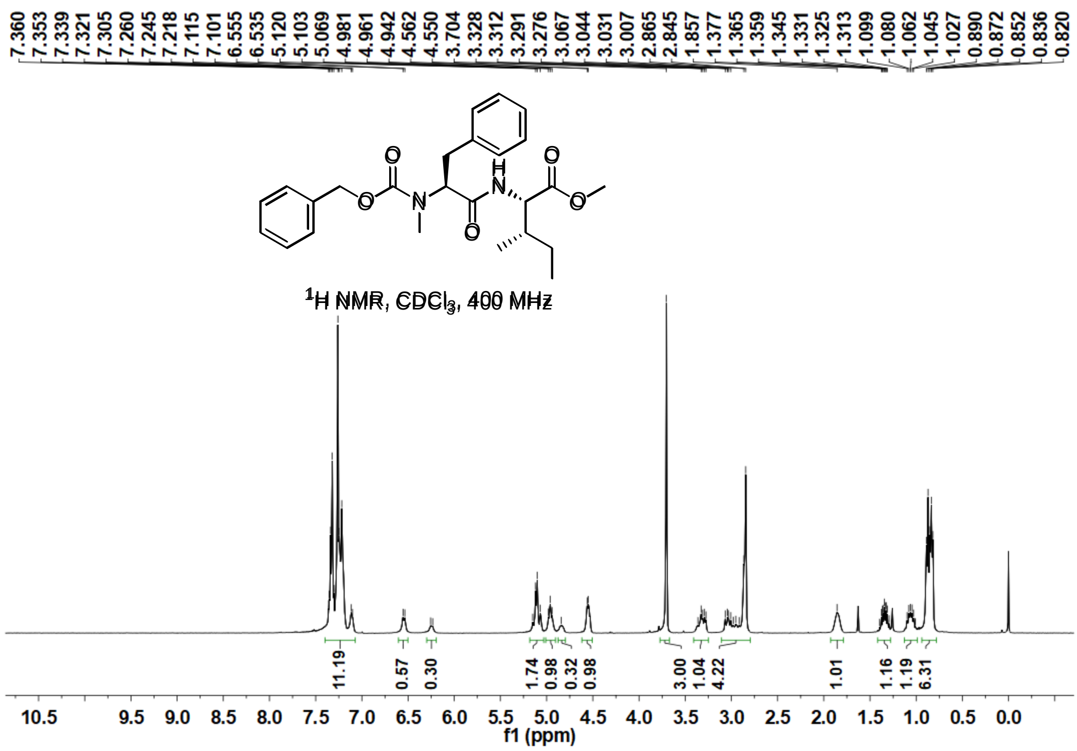

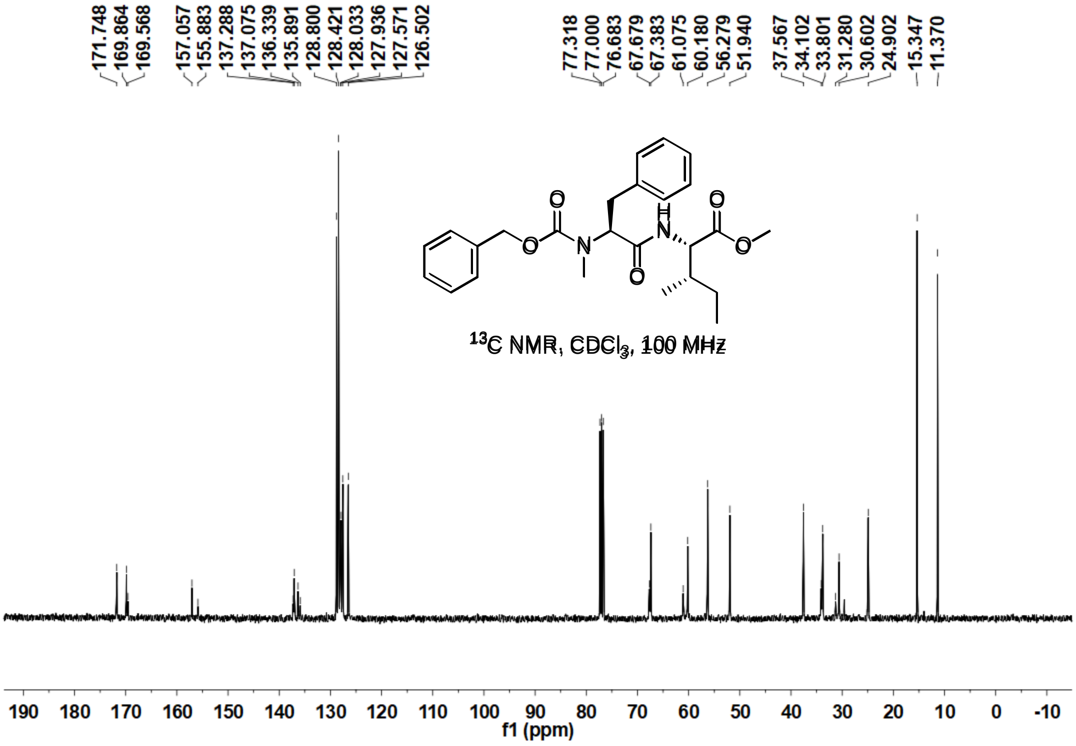

Cbz-L-NMePhe-L-His(Trt)-OMe (6-17)

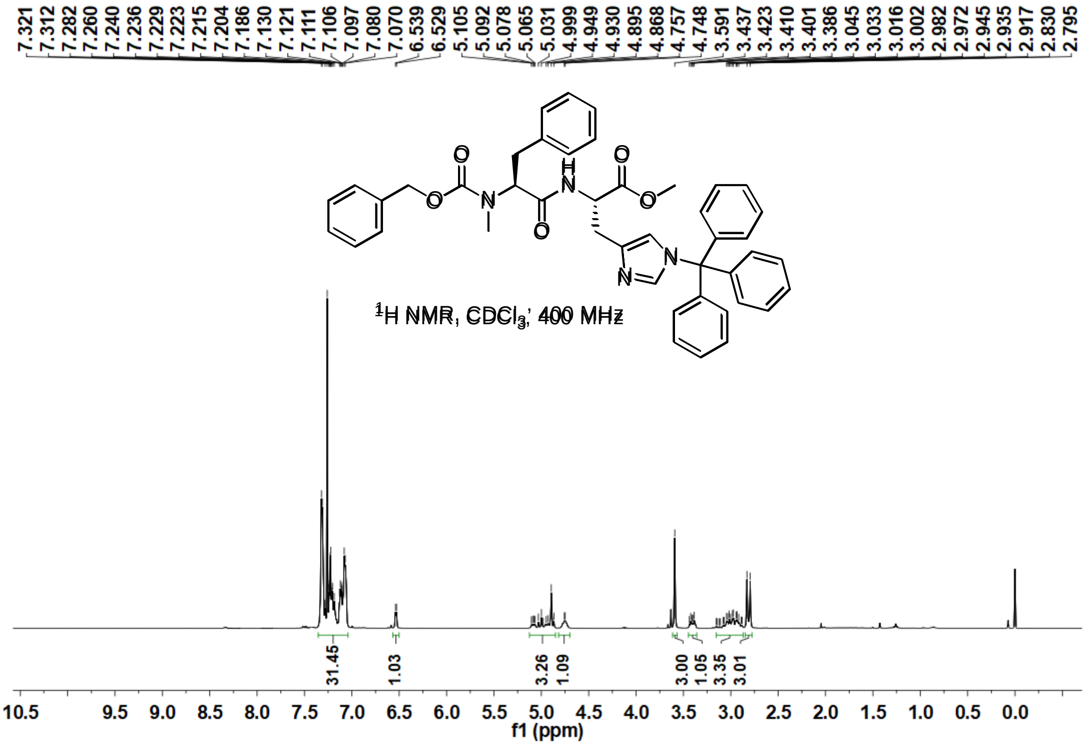

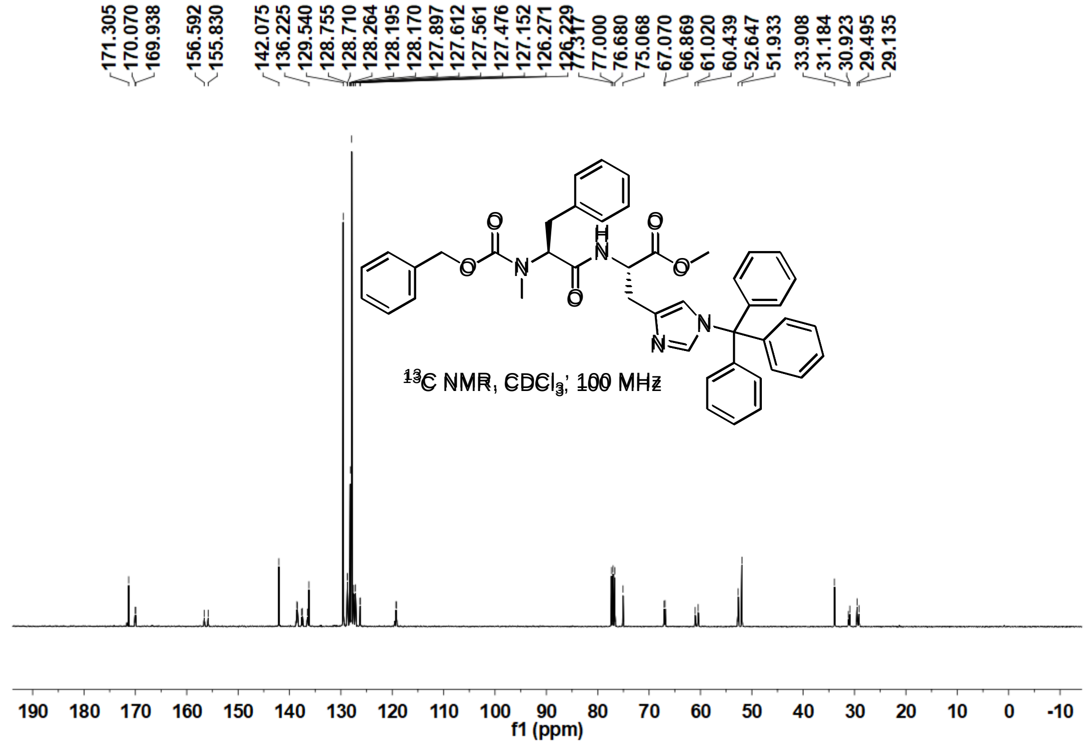

Cbz-L-NMePhe-L-Tyr(Bzl)-OMe (6-18)

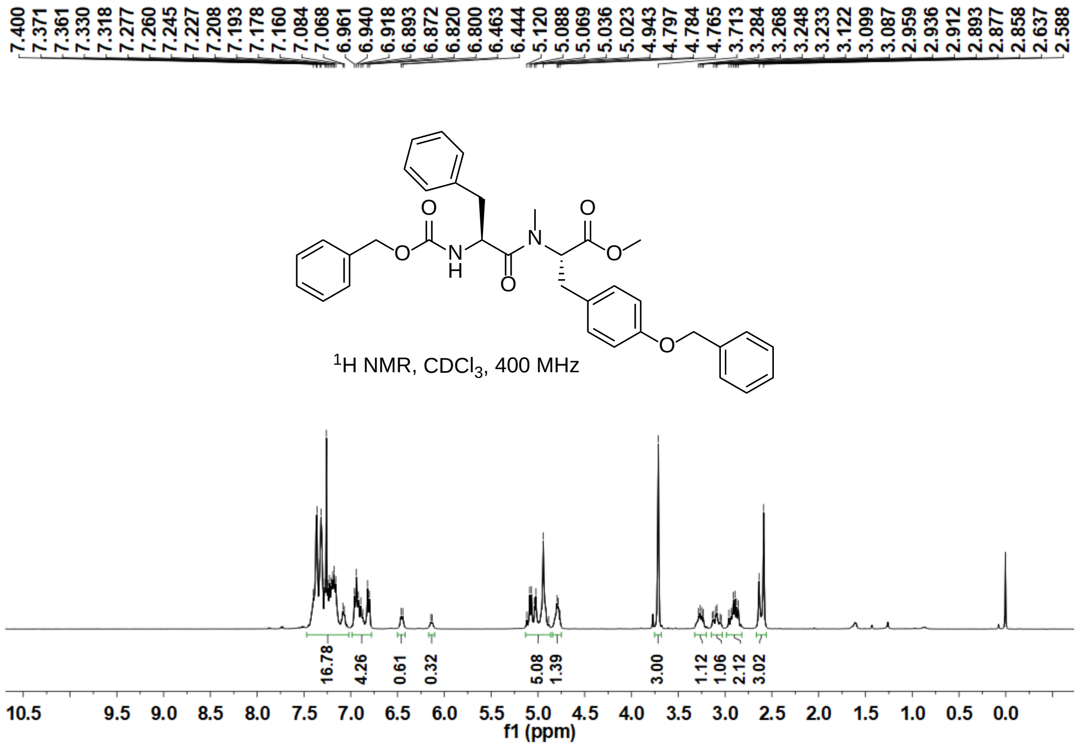

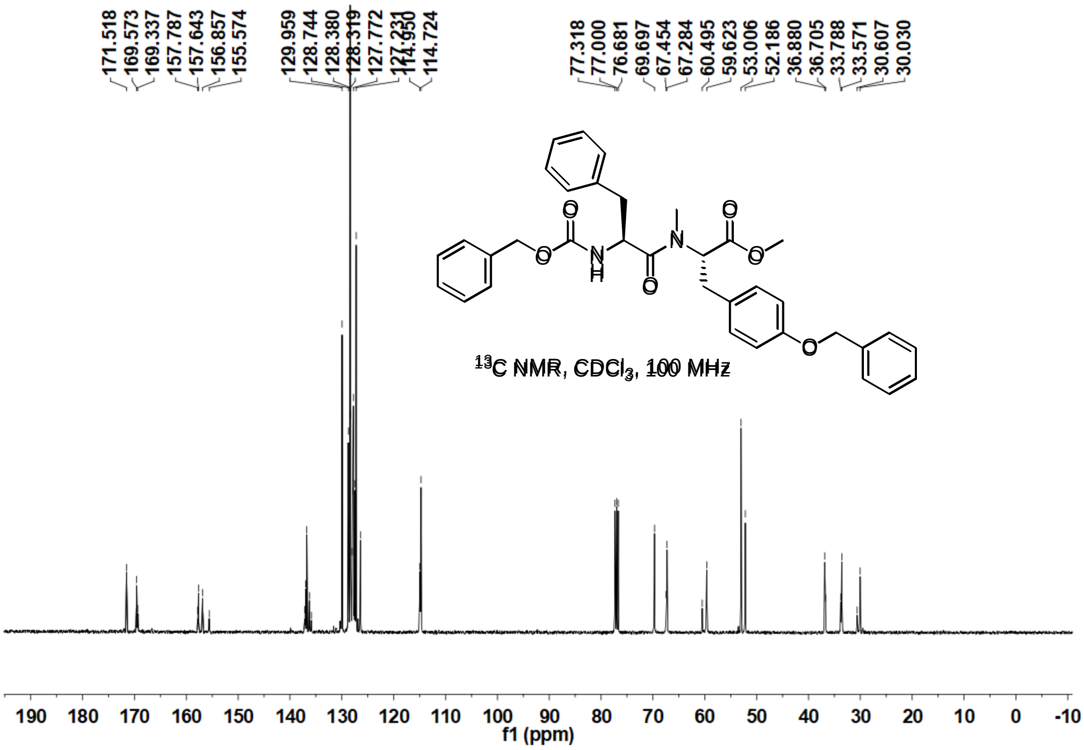

Cbz-L-NMePhe-L-Cys(Trt)-OMe (6-19)

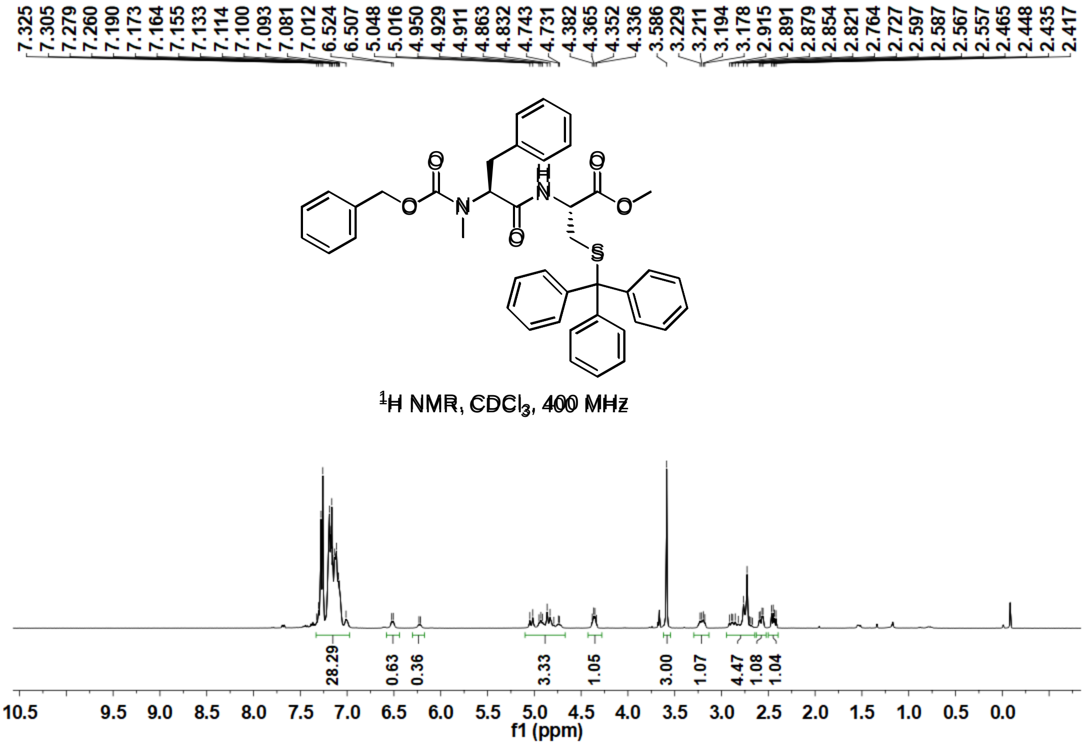

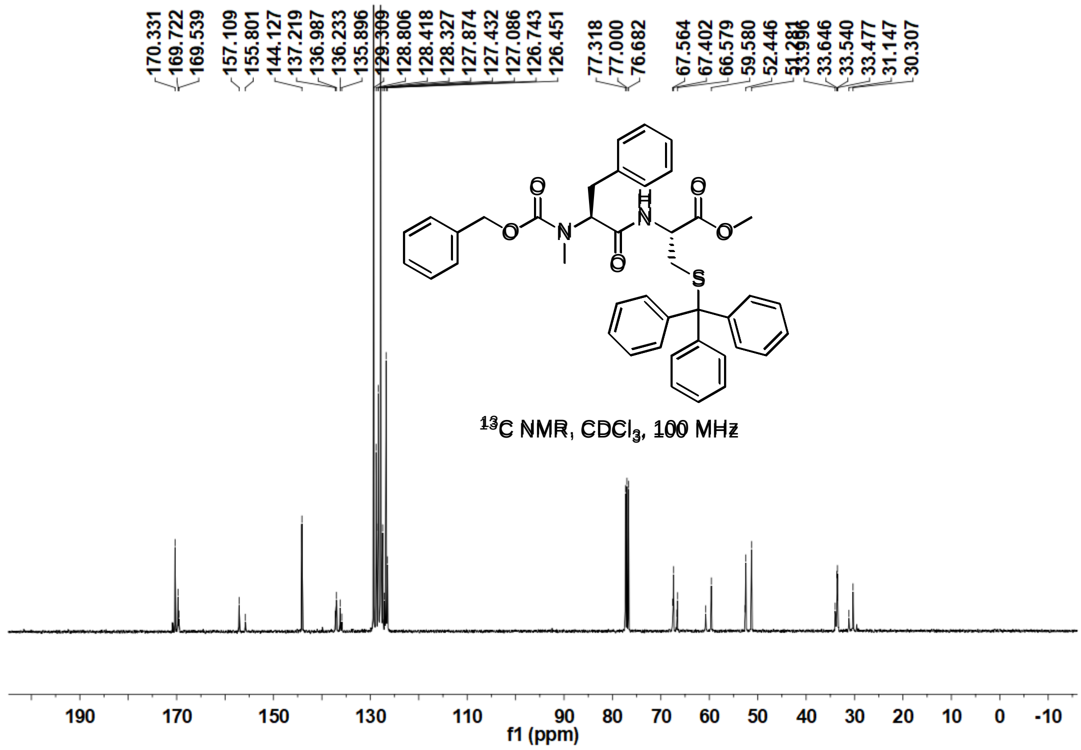

Cbz-L-NMePhe-L-Lys(Z)-OMe (6-20)

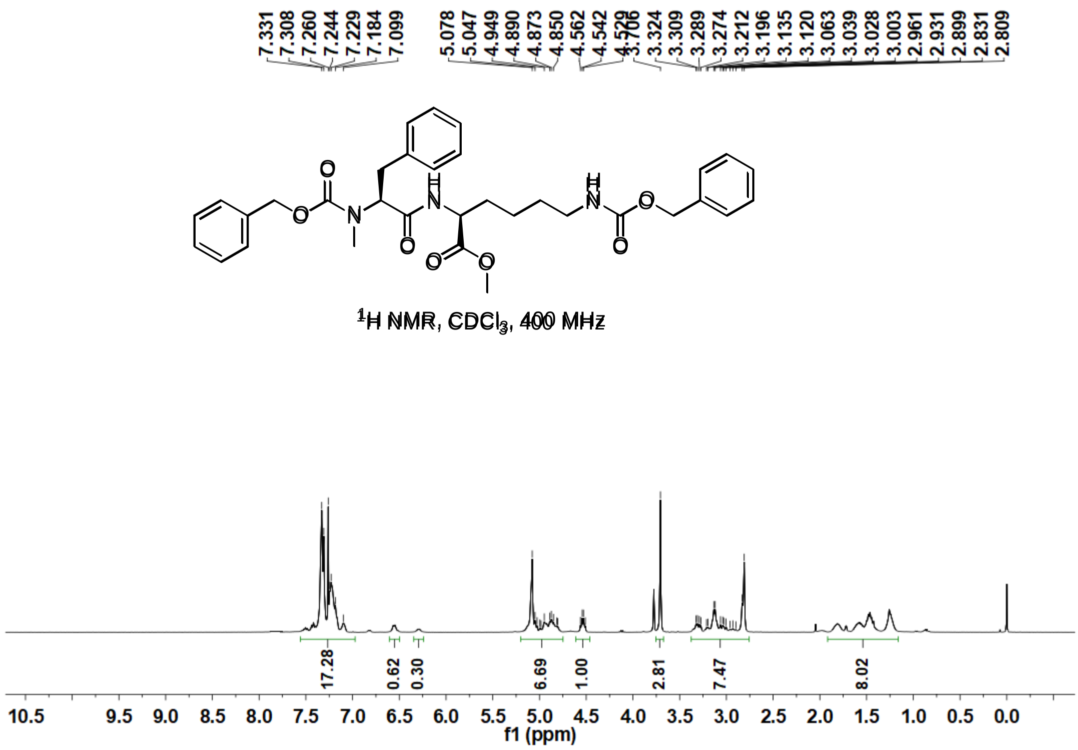

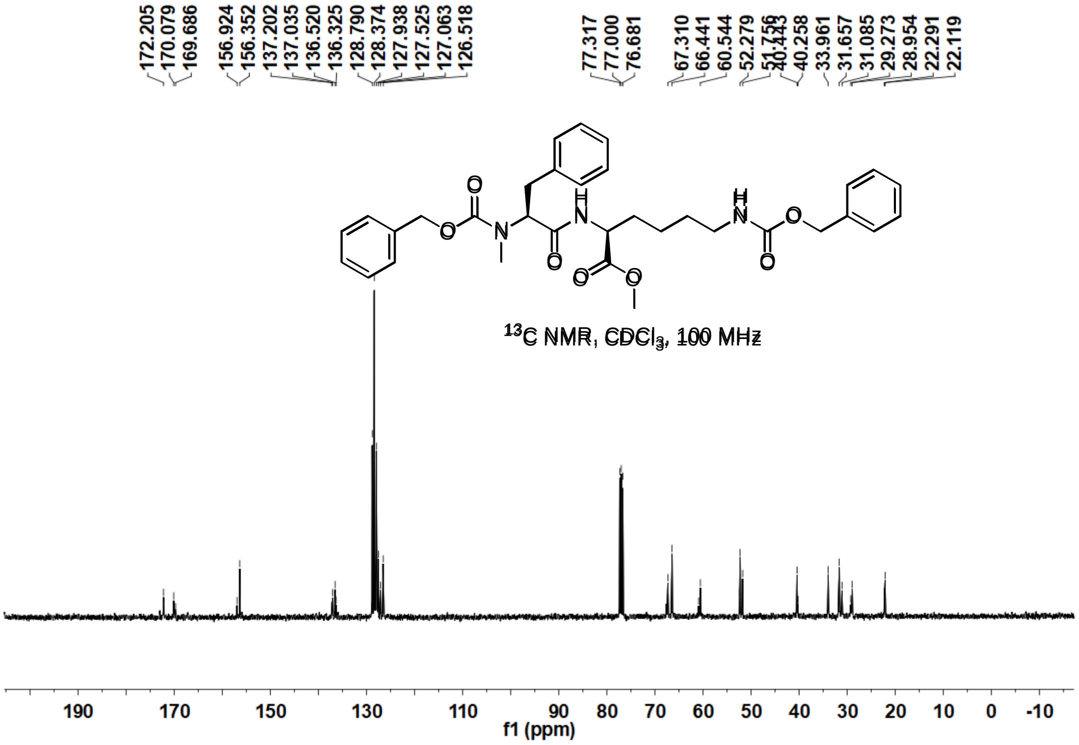

Cbz-L-NMePhe-L-Glu(OEt)-OEt (6-21)

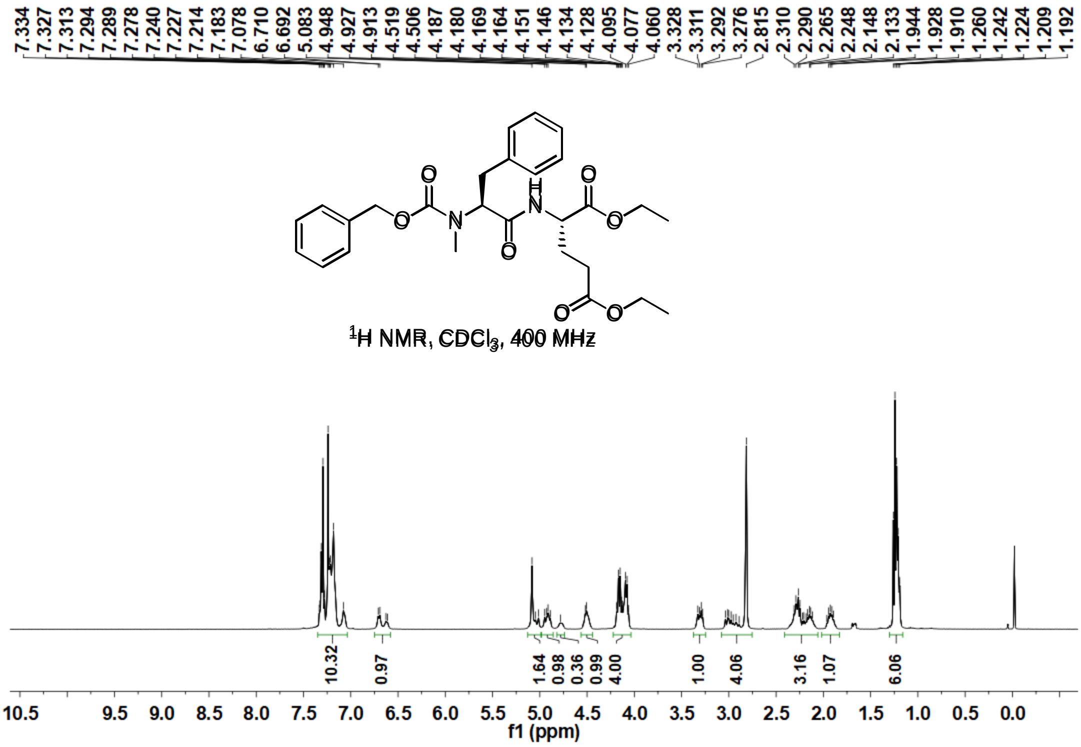

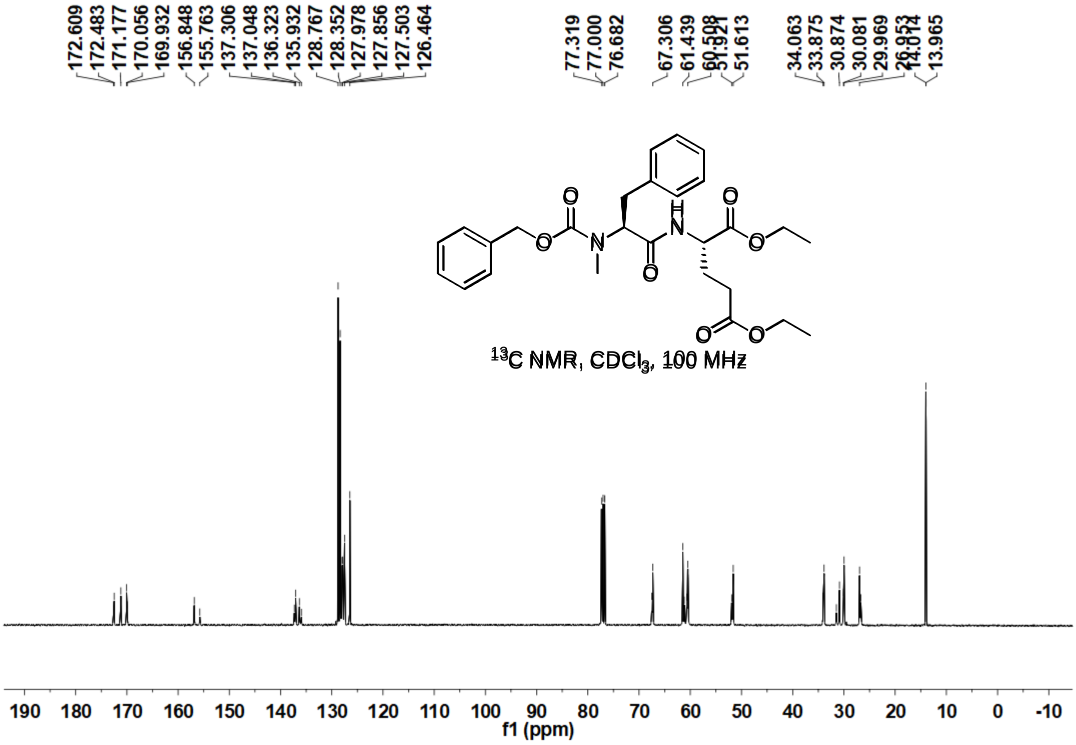

Cbz-L-NMePhe-L-Tyr-OMe (6-22)

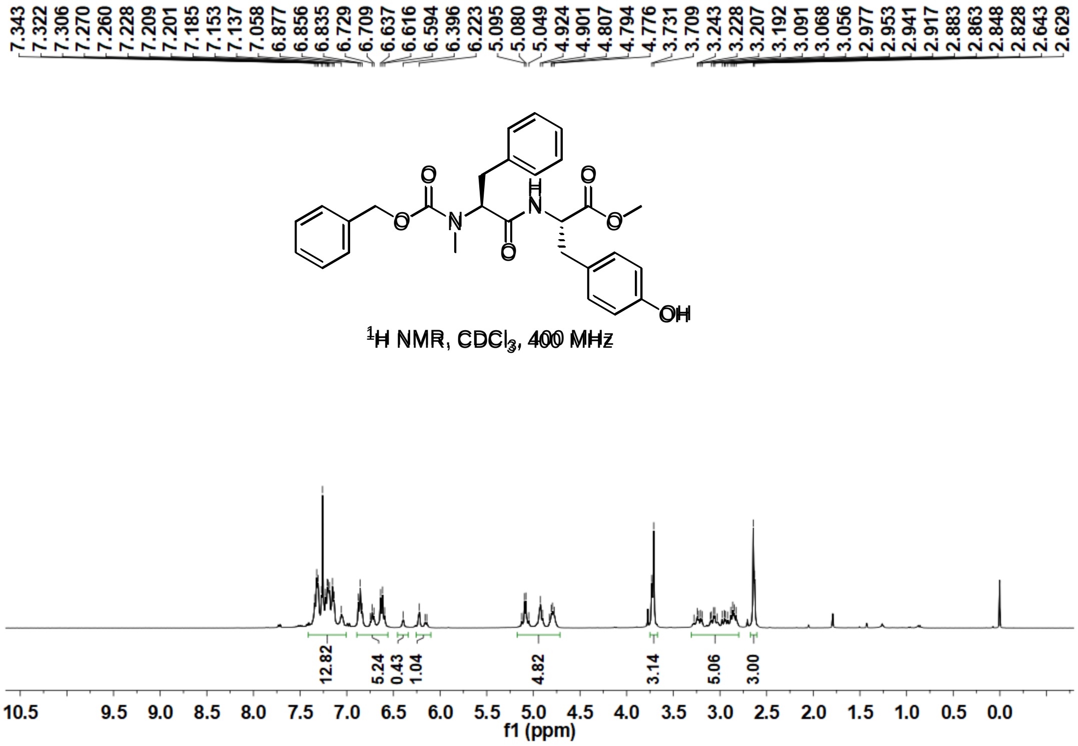

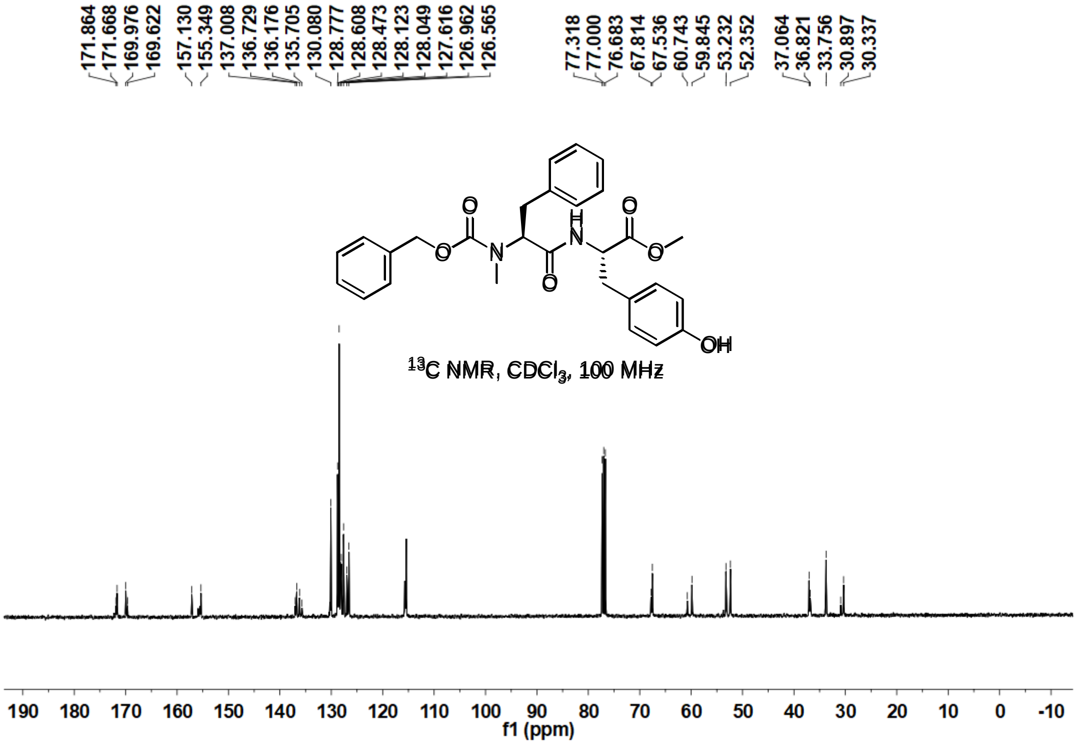

HO-Leu-Phe-Gly-Gly-Tyr-NH_2

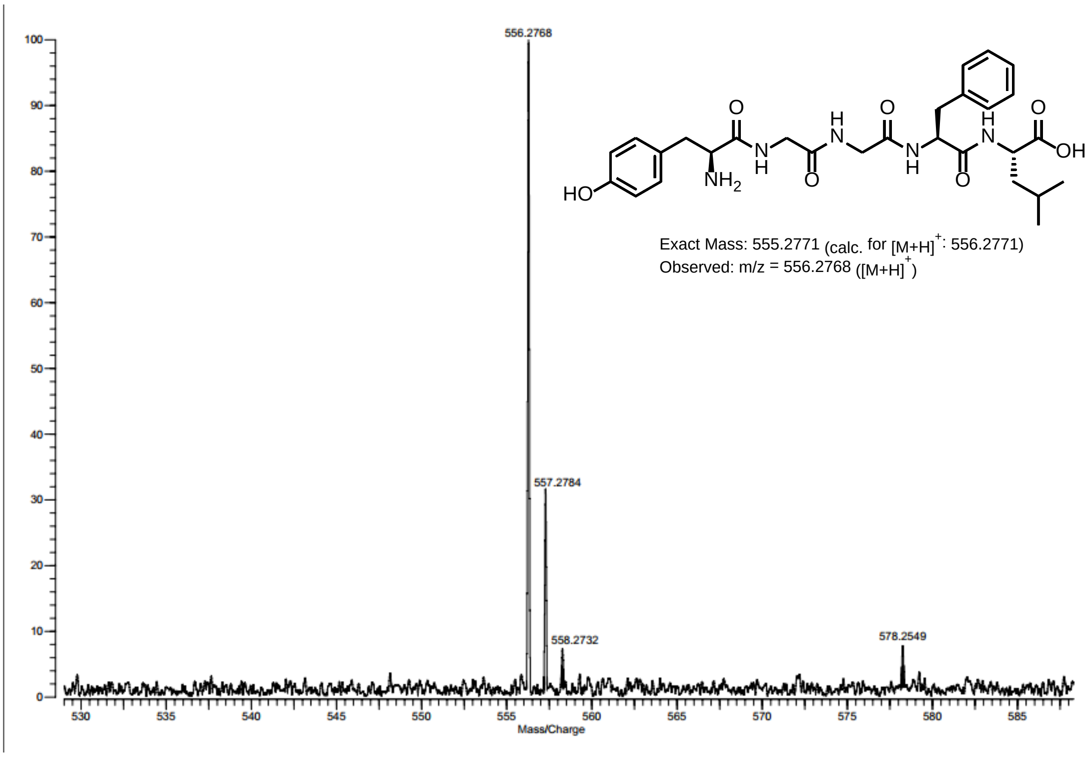
556.2768
557.2784
558.2732
578.2549
Mass/Charge
HO
NH_2
OH
Exact Mass: 555.2771 (calc. for $[M+H]^+$: 556.2771)
Observed: m/z = 556.2768 ($[M+H]^+$)
